# CRACD loss drives hypoxia-induced immune escape in gastric cancer

**DOI:** 10.1038/s41467-026-77348-9

**Published:** 2026-09-01

**Authors:** Yoojeong Seo, Jinho Jang, Kyung-Pil Ko, Gengyi Zou, Sang-Min Kim, Yuanjian Huang, Shengzhe Zhang, Jie Zhang, Sohee Jun, Wonhong Chu, Vishwa Venkatesan, Sanjana Dhakshinamoorthy, Hyunki Kim, Jae-Il Park

**Affiliations:** 1 Department of Experimental Radiation Oncology, Division of Radiation Oncology, The University of Texas MD Anderson Cancer Center, Houston, TX USA; 2 Department of Gastrointestinal Medical Oncology, The University of Texas MD Anderson Cancer Center, Houston, TX USA; 3 Department of Pharmacology, Toxicology & Neuroscience, Louisiana State University Health Sciences Center-Shreveport, Shreveport, LA USA; 4 Department of Pathology, Yonsei University College of Medicine, Seoul, Republic of Korea; 5 Graduate School of Biomedical Sciences, The University of Texas MD Anderson Cancer Center, Houston, TX USA; 6 Program in Genetics and Epigenetics, The University of Texas MD Anderson Cancer Center, Houston, TX USA

**Keywords:** Gastric cancer, Tumour biomarkers

## Abstract

Mucinous gastric adenocarcinoma (MGC) is a distinct histologic subtype characterized by poor prognosis and limited responsiveness to chemotherapy and immunotherapy. CRACD, an actin polymerization regulator, is frequently inactivated in gastric cancer (GC), with enrichment in mucinous subtypes. We found that genetic engineering of murine gastric organoids with *Cracd* ablation combined with *Kras* mutation and *Trp53* loss induced mucinous cell plasticity, disrupted epithelial integrity, and promoted immune evasion. Mechanistically, CRACD loss triggered actin dysregulation, which activated the NF-kB/COX2 signaling and NADPH oxidase-dependent ROS generation. The resulting oxidative stress stabilized HIF1α and transactivated *PD-L1*, thereby disrupting anti-tumor immunity. Pharmacologic inhibition of HIF1α or PD-L1 restored immune surveillance and suppressed tumorigenesis. Clinically, analysis of patient-derived organoids and tissue microarrays demonstrated the inverse correlation between CRACD and PD-L1 expression. These findings identify CRACD inactivation as a crucial event in mucinous plasticity and immune escape in GC, further proposing that the HIF1α-PD-L1 axis is a therapeutic vulnerability in CRACD-inactivated GC.

## Abstract (web summary)

Mucinous gastric adenocarcinoma is an aggressive subtype of gastric cancer with limited therapeutic options and poorly understood mechanisms of immune evasion. Here, the authors show that loss of the actin polymerization regulator CRACD promotes mucinous plasticity and immune escape by driving hypoxia-dependent upregulation of PD-L1.

## Introduction

Gastric cancer (GC) is the fifth most common malignancy and the fourth leading cause of cancer-related mortality worldwide, accounting for over 1 million new cases and approximately 769,000 deaths in 2020^1^. Although incidence rates have declined in some regions, GC remains a critical health burden in high-risk areas such as East Asia, South America, and Eastern Europe^2^. Its aggressive nature, driven by high rates of metastasis and resistance to conventional chemotherapy, results in a poor prognosis^3^. Tumor angiogenesis is crucial in GC progression and is associated with unfavorable survival outcomes^4^.

Mucinous gastric carcinoma (MGC) is a histological subtype of GC, characterized by abundant extracellular mucin comprising more than 50% of the tumor volume^5^. It accounts for approximately 2–9% of all GCs and displays clinicopathological features^6,7^, including larger tumor size, deeper invasion, and a high frequency of lymph node and peritoneal metastases^8^. MGC patients often show limited and heterogeneous responses to immune checkpoint blockade, underscoring the need for improved biomarkers and therapeutic strategies^9^.

Early detection remains a significant challenge owing to the lack of symptoms in early-stage GC. Traditional biomarkers, including CEA and CA19-9, are limited in sensitivity and specificity. Recent research highlights the potential of novel biomarkers such as FGFR2 and E-cadherin, and emerging noninvasive markers, including circulating tumor cells and microRNAs, to enhance early diagnosis and treatment strategies^10–12^. GC often metastasizes to lymph nodes, peritoneum, and, in women, ovaries, and has unique genetic mutations influencing metastatic patterns. Management of advanced GC typically involves a multidisciplinary approach, including chemotherapy, targeted therapies, and immunotherapy^13–17^. Despite such advances, challenges such as therapy resistance and relapse persist^13–19^. Moreover, patient stratification for specific therapies is another hurdle to be overcome.

Hypoxia, a hallmark of the tumor microenvironment, induces significant cellular stress and promotes the production of reactive oxygen species (ROS)^20^. As tumors grow, their rapid proliferation outpaces the development of new blood vessels, leading to regions within the tumor that are poorly oxygenated^21^. This hypoxic microenvironment is not just a consequence of rapid growth but a key driver of GC aggressiveness and therapeutic resistance. Hypoxia induces various adaptive responses in cancer cells that promote their survival, growth, and metastasis^22^. These responses are mediated by hypoxia-inducible factors (HIFs), particularly HIF1α, which regulates gene expression involved in angiogenesis, metabolism, cell survival, and invasion. ROS are key regulators of various cellular processes, including the dynamics of the actin cytoskeleton^23,24^. Actin dynamics, regulated by several actin-binding proteins, control the processes of polymerization and depolymerization. This enables cancer cells to remodel their cytoskeletons in response to external signals like hypoxia and migrate through the extracellular matrix. Actin cytoskeleton remodeling is crucial for cell motility, invasion, and metastasis^25,26^. Dysregulation of actin polymerization pathways is often observed in many types of cancer and is associated with enhanced metastatic potential and poor prognosis^27^. Further, under hypoxic conditions, disruption of the actin pathway allows cancer cells to evade immune surveillance through several mechanisms, including remodeling of the actin cytoskeleton at the immunological synapse, regulation of vasculogenic mimicry, and modulation of actin-binding proteins^28^. Emerging evidence suggests that ROS can modulate actin-binding proteins, leading to changes in their activity and affinity for actin filaments^24^. This process can disrupt the balance between actin polymerization and depolymerization, resulting in cytoskeletal reorganization that affects cell shape, motility, and invasion^29^. However, the impact of actin dysregulation on ROS and HIF pathways is unknown.

CRACD (Capping protein inhibiting Regulator of Actin Dynamics; also referred to as CRAD/KIAA1211) is a tumor suppressor that participates in maintaining epithelial cell integrity^30^. CRACD promotes actin polymerization by inhibiting capping proteins (CAPZA and CAPZB), which are negative regulators of actin polymerization^30^. This mechanism is vital for preventing uncontrolled cellular proliferation and tumorigenesis. CRACD is often inactivated in several types of cancer. In colorectal cancer, the loss of CRACD function destabilizes the cadherin-catenin-actin cytoskeleton complex, thereby releasing β-catenin and hyperactivating WNT signaling. This mechanism is linked to the development of intestinal mucinous adenomas^30^. In lung adenocarcinoma, CRACD loss is associated with the emergence of neuroendocrine cell plasticity^31,32^.

Here, we investigate how CRACD inactivation shapes mucinous gastric tumorigenesis and antitumor immunity. Using genetically engineered gastric organoids, syngeneic and orthotopic transplantation models, and single-cell transcriptomics, we show that CRACD loss promotes mucinous cell plasticity and immune escape. Mechanistically, CRACD deficiency disrupts actin organization and activates NF-κB/COX-2 signaling and NOX1-dependent ROS production, thereby stabilizing HIF1α and inducing PD-L1 expression. Analyses of patient-derived organoids and gastric cancer tissue microarrays further reveal an inverse association between CRACD and PD-L1 expression. Pharmacologic inhibition of HIF1α or blockade of PD-L1 restores antitumor immune activity and suppresses CRACD-deficient tumor growth, identifying the HIF1α-PD-L1 axis as a potential therapeutic vulnerability in CRACD-inactivated gastric cancer.

## Results

### *Cracd* KO-induced hyperplasia of gastric epithelial cells

To define the gene alteration status of CRACD in cancer, we analyzed the Cancer Genome Atlas (TCGA) datasets from cBioPortal. The *CRACD* gene is commonly mutated in GC, including mucinous stomach adenocarcinoma, signet ring cell carcinoma of the stomach, diffuse gastric adenocarcinoma, tubular adenocarcinoma, and stomach adenocarcinoma (Supplementary Fig. 1A). Immunostaining of GC tissue microarrays revealed downregulation of CRACD protein expression in GC samples but ample expression of CRACD in the gastric epithelial cells of normal stomach tissue (Supplementary Fig. 1B, C). These results indicate that CRACD is often inactivated in GC.

Having determined that CRACD is downregulated in GC (Supplementary Fig. 1), we hypothesized that CRACD loss-of-function contributes to GC tumorigenesis. To test this, we analyzed the stomach tissues of *Cracd* knockout (KO) mice (Supplementary Fig. 2A). Compared with the control (*Cracd* wild-type [WT] mouse stomach), the *Cracd* KO mouse stomach displayed hyperplasia, cell hyperproliferation, and increased mucin deposition (Supplementary Fig. 2B–E).

**Fig. 1:**
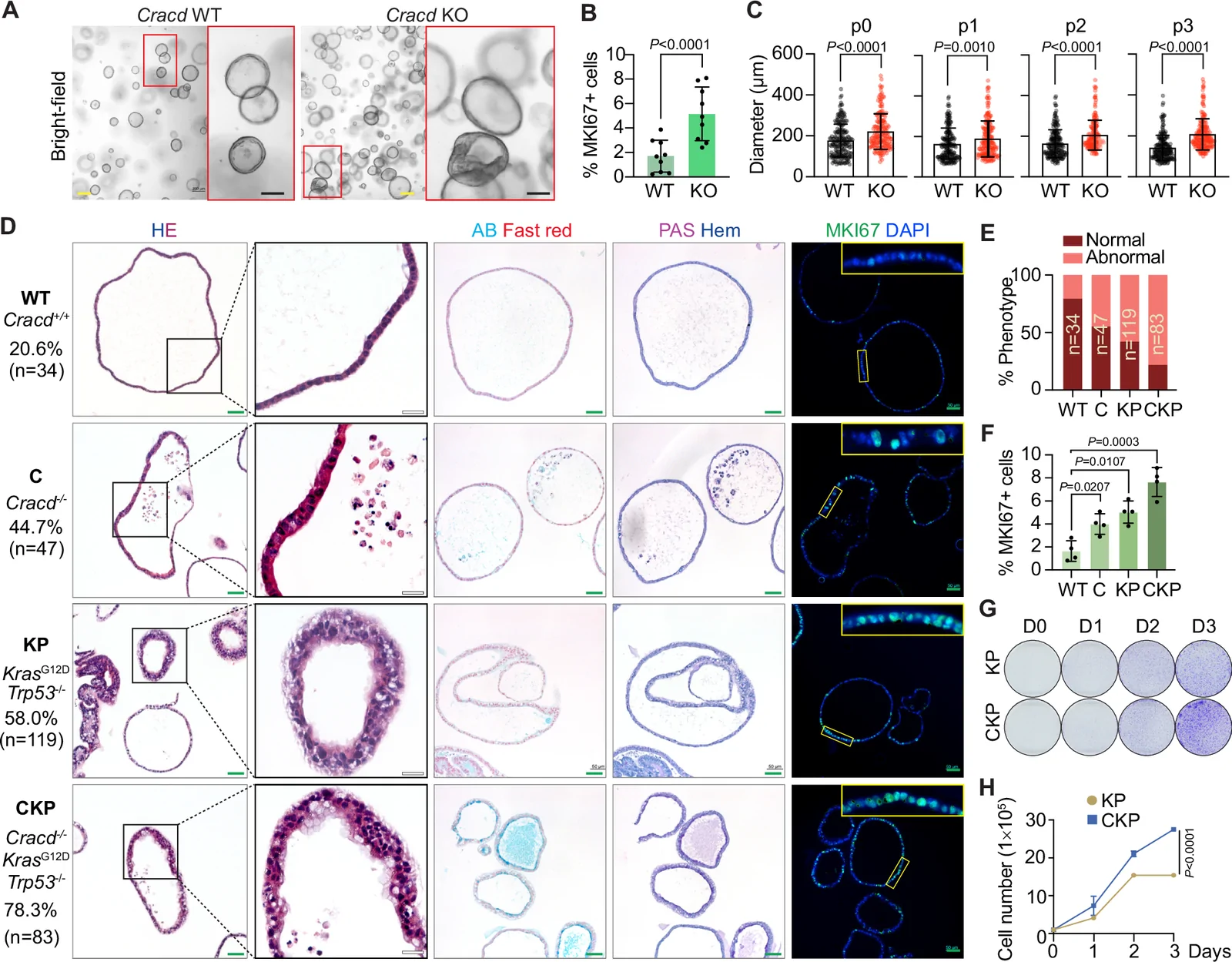
*Cracd* KO induces hyperplasia of gastric epithelial cells. **A** Representative bright-field images of *Cracd* wild-type (WT) and knockout (KO) murine gastric organoids (GOs). Scale bars, 200 μm (yellow) and 100 μm (black). **B** Quantification of MKI67-positive cells in WT and KO GOs. MKI67-positive cells were quantified in multiple microscopic fields; measurements were averaged for each of nine biologically independent experiments (*n* = 9), and each dot represents one independent experiment. **C** Organoid diameter at passages 0-3. Each dot represents one organoid; organoids were analyzed across three biologically independent experiments (*n* = 3). **D** Histologic analysis of *Cracd* WT, *Cracd*^-/-^ (C), *Kras*^*G12D*^ *Trp53*^-/-^ (KP), and *Cracd*^-/-^ *Kras*^*G12D*^ *Trp53*^-/-^ (CKP) GOs by hematoxylin and eosin (HE), Alcian blue (AB), periodic acid-Schiff (PAS), and MKI67 staining. Scale bars: green, 50 μm; black, 100 μm. Percentiles indicate the ratio of abnormal morphology, such as vacuolization and cell adhesion loss, mucinous features, and cell hyperplasia. **E** Quantification of normal and abnormal organoid morphology, including vacuolization, loss of cell-cell adhesion, mucinous features, and epithelial hyperplasia. n denotes individual organoids scored (WT, *n* = 34; C, *n* = 47; KP, *n* = 119; CKP, *n *= 83). **F** Quantification of MKI67-positive cells in WT, C, KP, and CKP GOs. Measurements from multiple microscopic fields were averaged for each of four biologically independent experiments (*n* = 4); each dot represents one independent experiment. **G** Crystal violet staining of KP and CKP cells in 2D culture (Day 0, 1, 2, and 3). **H** Cell proliferation of KP and CKP cells. Experiments were performed independently three times, each with triplicate wells (*n* = 3 biologically independent experiments). Data in (**B**, **C**, **F**, and **H**) are presented as mean ± SD. Statistical significance was assessed using two-tailed unpaired Student’s *t* tests.

We also prepared gastric organoids (GOs) by isolating epithelial cells from the stomach tissues of *Cracd* WT and KO mice and culturing them into GOs. Compared with WT, the *Cracd* KO GOs exhibited cell hyperproliferation (Supplementary Fig. 2F–H). Further, in a differentiation culture condition (withdrawal of Wnt3A, R-Spondin, and Noggin, key factors in maintaining the stemness of GOs), unlike WT GOs, the *Cracd* KO GOs exhibited mucin deposition in the lumen and loss of cell polarity and adhesion (Supplementary Fig. 2I–L). *Cracd* KO GOs also displayed accelerated GO growth over multiple passages (Fig. 1A–C). In addition to CRACD, the receptor tyrosine kinase (RTK)-RAS and TP53 pathways are often altered in GC^33,34^. We recently reported that RAS activation (*Kras*^G12D^) combined with TP53 inactivation (*Trp53* KO) was sufficient to induce the development of intestinal-type gastric tumors^35^. To define the genetic interaction of *CRACD* loss with RTK-RAS and TP53 signaling, we used genetically engineered GOs^35^. By using the Cre-loxP recombination and CRISPR-based genetic manipulation, we established GOs carrying *Kras*^G12D^ activation and *Trp53* deletion in combination with *Cracd* KO to create *Cracd*^*-*/*-*^ (C) cells; *Kras*^*G12D*^ *Trp53*^*-*/*-*^ (KP) cells; and *Cracd*^*-*/*-*^ *Kras*^*G12D*^ *Trp53*^*-*/*-*^ (CKP) cells (Fig. 1D and Supplementary Fig. 2K–N). Compared with *Trp53*^*-*/*-*^, KP and CKP GOs grew faster and expressed carcinoma embryonic antigen (CEA5), a clinical marker of gastrointestinal tract tumors (Supplementary Fig. 2O–Q).

*Cracd* WT GOs develop a single layer of epithelial cells. However, C, KP, and CKP GOs displayed aberrant epithelial layer formation (Fig. 1D), with CKP GOs showing the most significant abnormal morphology, including vacuolization and cell adhesion loss (78.3 %, *n* = 83), mucinous features, and cell hyperplasia (Fig. 1D–F). We also established GO-derived cell lines in 2D culture by using minimum growth factors^35–37^. Unlike WT and C cells, KP and CKP cells grew and maintained well at multiple passages. Consistent with the GO findings, the CKP cells showed increased colony formation and cell proliferation relative to KP cells (Fig. 1G, H). These results suggest that CRACD loss is sufficient to induce mucinous hyperplasia of the gastric epithelium.

### Cracd depletion induces cell plasticity that mirrors the transcriptional signature of gastric neoplasia

Having observed the gastric hyperplasia induced by *Cracd* KO (Fig. 1), we next investigated the cellular mechanisms provoked by *Cracd* loss. We conducted single-cell RNA sequencing (scRNA-seq) of *Cracd* WT, C, KP, and CKP GOs (Supplementary Fig. 3A). Cell clusters were identified based on expression of the top 5000 genes. All datasets were integrated with the Harmony algorithm to remove the batch effect. Unsupervised cell clustering generated seven cell clusters (Supplementary Fig. 3B, C). Compared with *Cracd* WT GOs (WT and KP), *Cracd* KO GOs (C [non-tumor] and CKP [tumor]) showed relatively smaller numbers of Aqp5^high^ cells (Aqp5 + ) and showed increased numbers of Mki67^+^ cells (proliferative) and G2/M phase cells (Supplementary Fig. 3B–F and Supplementary Fig. 4A, B), implying that *Cracd* KO affects gastric epithelial cell lineages. Analyses of cell lineage trajectories using CytoTRACE UMAPs and pseudotime approaches showed marked alterations in the cell trajectory of *Cracd* KO (WT vs. C; KP vs. CKP) (Supplementary Fig. 3G, H). Whereas the Aqp5+ cell cluster served as a cellular origin in *Cracd* WT GOs (WT and KP), the Proliferative cell cluster (Mki67 + ) was the primary cellular origin of *Cracd* KO GOs (C and CKP) (Supplementary Figs. 3G, H, 4B). Likewise, consistent with the results of cell proportion analysis, the expression of *Mki67*, *Sox4*, and *Lgr5*, all key markers of cell proliferation and stem cell activity, was increased in CKP (Supplementary Fig. 4A, B). In contrast, the gastric stem cell markers *Hopx*, *Lrig1*, *Prom1*, and *Aqp5* were decreased in the *Cracd* KO GOs (C and CKP) compared with WT GOs (Supplementary Fig. 4A, B). Mucinous markers, such as *Muc1*, were also increased in the *Cracd* KO organoids (C and CKP GOs) (Supplementary Fig. 4C), consistent with Alcian blue/periodic acid-Schiff staining results (Fig. 1D).

Next, we assessed the influence of CRACD depletion on cell plasticity by comparative analysis of cell plasticity potential (CPP) calculated by cell entropy, i.e., the degree of heterogeneity of gene expression and spatial information of each cell cluster visualized on PHATE maps^38^ as reconstructed by using Waddington landscape^31,39^ (Supplementary Fig. 3I). In the *Cracd* WT GOs (WT and KP), the CPP was highest in the Aqp5+ cell cluster, but in *Cracd* KO GOs (C and CKP), the CPP was higher in Proliferative cell clusters than in the Aqp5+ cell cluster (Supplementary Fig. 3D, E). We also determined the pathological relevance of our models to human GC by analyzing the expression of clinical markers: *Krt7*, *Krt20*, and *Cdx2* for gastric adenocarcinoma (GAC); *Muc1*, *Muc2*, and *Muc5ac* for differentiation diagnosis; and *Sox2* and *Sox4* for undifferentiation (or stemness) markers. Dot and feature plots revealed marked increases in GAC markers in *Cracd* KO GOs (C and CKP) (Supplementary Fig. 4D, E). These results suggest that CRACD depletion induces cell plasticity that drives the transcriptional signature of gastric neoplasia.

### *Cracd* KO with *Kras*^G12D^*Trp53* KO promotes gastric tumorigenesis with immune evasion

**Fig. 2:**
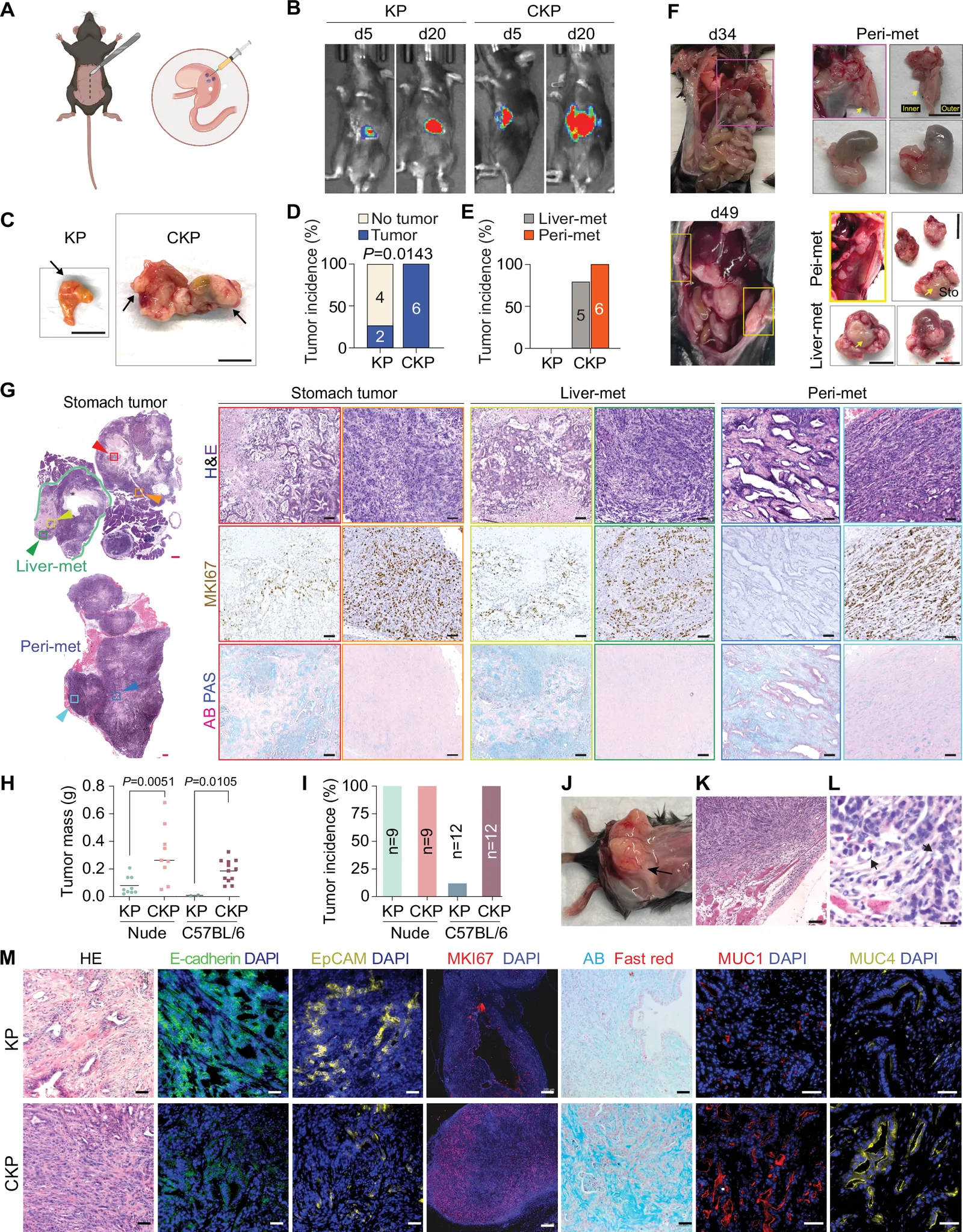
CRACD depletion drives mucinous differentiation and metastasis. **A** Schematic of the orthotopic gastric cancer model. KP or CKP cells were surgically injected into the gastric wall of C57BL/6. The schematic was created with BioRender.com. **B** Representative bioluminescence images showing tumor growth in KP and CKP orthotopic models. **C** Gross images of representative gastric tumors from KP and CKP mice (arrows indicate tumor masses). **D** Tumor incidence in the KP and CKP groups (*n* = 6 mice per group). Tumor incidence was compared using a two-sided chi-square test; *P* = 0.0143. **E** Incidence of liver and peritoneal metastases in the KP and CKP groups. Numbers within the bars indicate the number of affected mice. **F** Representative images of peritoneal and liver metastases in CKP-bearing mice (arrows). **G** Histological analysis of primary gastric tumors, peritoneal and liver metastases by H&E, AB-PAS, and MKI67 immunohistochemistry staining. Scale bars: 1 cm (gross images), 500 μm (low magnification), 50 μm (high magnification). **H** Quantification of tumor mass in nude and C57BL/6 mice bearing KP or CKP tumors. Each dot represents an individual mouse. Data are presented as mean ± SD. Statistical significance was determined using two-tailed unpaired Student’s *t* tests; nude mice, *P* = 0.0051; C57BL/6 mice, *P* = 0.0105. **I** Tumor incidence in nude and C57BL/6 mice bearing KP or CKP tumors (*n* = 9 mice per group for nude mice and *n* = 12 mice per group for C57BL/6 mice). **J** Representative photograph of CKP allograft tumors. **K**,** L** Histologic images of CKP tumors isolated from C57BL/6 mice, showing tumor invasiveness. Scale bars: 50 μm. Images are representative of three biologically independent mice. **M** Representative immunofluorescence images of KP and CKP tumors from three biologically independent mice. Scale bars, 200 μm (low magnification) and 50 μm (high magnification).

Based on the tumorigenic phenotypes in *Cracd* KO organoids, we investigated the effect of CRACD loss on gastric tumorigenesis in vivo by performing orthotopic gastric injection of KP and CKP cells derived from C57BL/6 mice (Fig. 2A). Tumor formation was monitored by in vivo imaging system (IVIS) imaging, which revealed that while only 2 out of 6 KP-injected mice developed primary gastric tumors, all 6 CKP-injected mice developed gastric tumors (Fig. 2B–D). Moreover, CKP tumors exhibited aggressive dissemination, with both liver and peritoneal metastases observed (Fig. 2C–E). Over time, metastatic spread progressively worsened, especially to the peritoneum and liver (Fig. 2F and Supplementary Fig. 5A, B). Histological analysis of primary and metastatic tumors showed abundant mucinous features (AB-PAS), and Mki67 staining was generally weaker in the tumor core, indicating poor differentiation. In contrast, peripheral tumor regions displayed reduced mucin accumulation but stronger Ki67 expression, consistent with proliferative activity (Fig. 2G). In addition, consistent with the observations in organoid results (Supplementary Fig. 2), immunofluorescence analysis revealed that actin and E-cadherin co-localized in *Cracd* WT gastric tissues, reflecting preserved epithelial integrity, whereas Cracd loss (KO or CKP orthotopic tumors) led to markedly diminished actin staining and aberrant E-cadherin expression (Supplementary Fig. 5C), further supporting disrupted epithelial organization.

Given the higher tumor incidence of CKP cells than that of KP cells (Fig. 2D, I), we determined whether *Cracd* KO CKP cells evade immune surveillance. In immunocompromised (Nude) recipient mice subcutaneously transplanted with KP or CKP cells, the tumors derived from CKP cells were larger than the tumors from KP cells (Fig. 2H, I). We also transplanted KP or CKP cells into immunocompetent mice (C57BL/6). Unlike in Nude mice (100% tumor incidence), KP cells rarely developed tumors in C57BL/6 mice (16.7% tumor incidence). In contrast, CKP cells generated tumors with 100% incidence in both Nude and C57BL/6 mice (Fig. 2I). These data indicate that CKP cells can evade immune surveillance, a feature not observed in KP cells. Moreover, unlike KP tumors, CKP tumors displayed pronounced invasiveness, penetrating the muscularis propria, increased angiogenesis, and higher vascular density, as well as exhibited DGAC hallmarks, including signet-ring cell carcinoma morphology (Fig. 2J–L). Histologic analysis revealed that CKP tumors were more proliferative and mucinous than KP tumors (Fig. 2M and Supplementary Fig. 6A, B). These findings demonstrate that CKP cells possess enhanced tumorigenic capacity, driven by increased proliferative activity and an ability to circumvent host immune defenses.

**Fig. 3:**
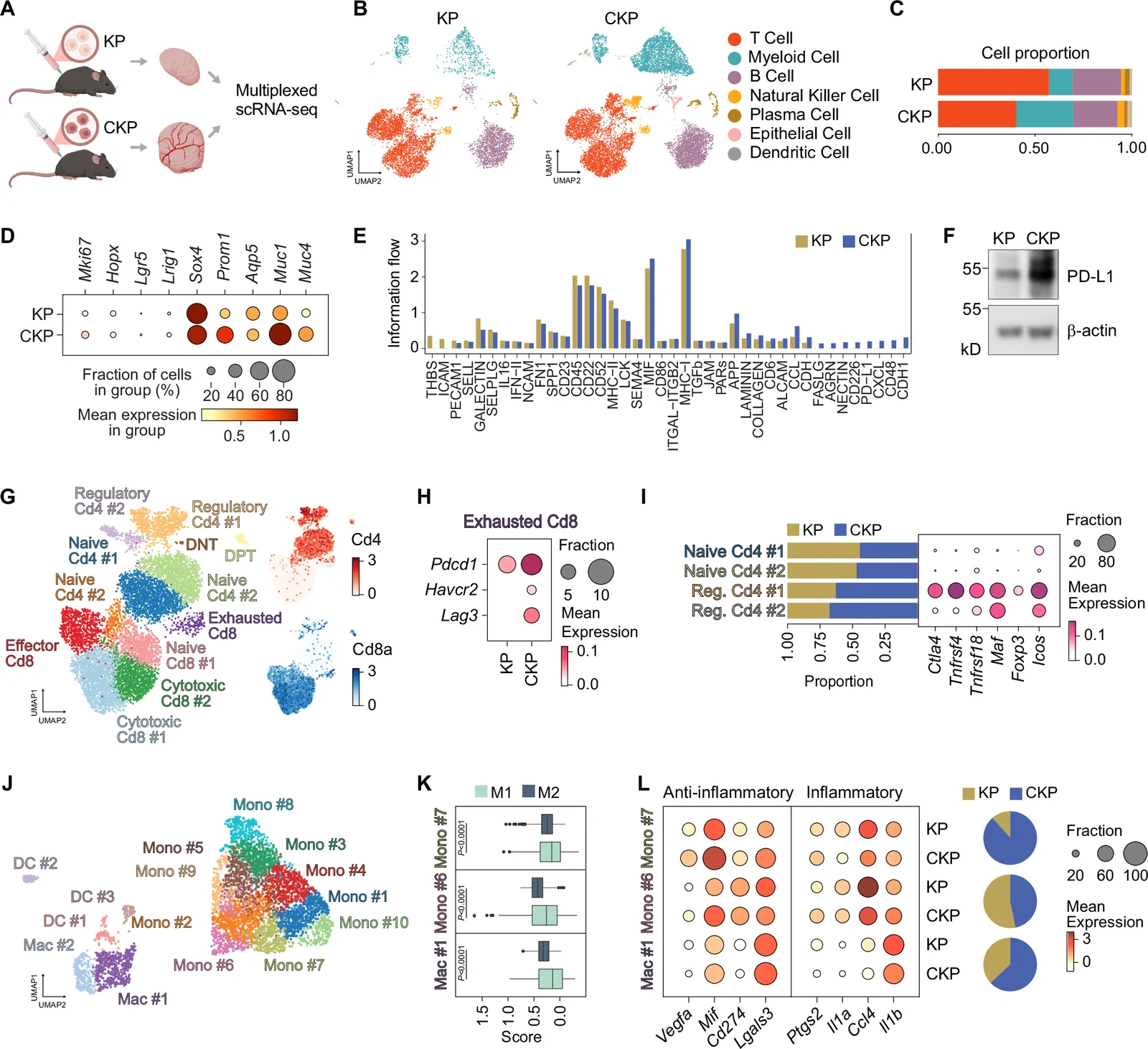
Single-cell transcriptomic analysis of subcutaneous tumors reveals mucinous differentiation upon CRACD loss. **A** Schematic representation of the workflow for scRNA-seq of allograft KP and CKP tumors. The schematic was created with BioRender.com. **B** UMAP plots of KP and CKP tumors isolated from C57BL/6 mice. **C** Bar plot showing the enrichment patterns of clusters in each KP and CKP tumor. **D** Dot plot displaying the expression profiles of mucinous markers in KP and CKP tumor cells. **E** Overall information flow of the PD-L1 signaling pathway in KP and CKP tumors; CellChat analysis. **F** Immunoblots of KP and CKP cells. Scale bars: black or white, 50 μm; yellow, 200 μm. **G** UMAP plot illustrating the clustering of T cells from KP and CKP tumors. **H** Dot plot displaying the expression profiles of immune checkpoint genes in the exhausted CD8^+^ T-cell subcluster (Subcluster 8) from KP and CKP tumors. **I** Bar plot showing the enrichment patterns of T cells from KP and CKP tumors in each subcluster, accompanied by a dot plot depicting the expression profiles of Treg marker genes in CD4^+^ T-cell subclusters. **J** UMAP plot illustrating the clustering of myeloid cells from KP and CKP tumors. **K** Box plots showing the M1 and M2 polarization scores in Mac #1, Mono #6, and Mono #7 subclusters, highlighting their significantly higher M2 polarization scores. Cell numbers were as follows: Mac #1, KP (*n* = 162) and CKP (*n* = 275); Mono #6, KP (*n* = 186) and CKP (*n* = 163); and Mono #7, KP (*n *= 40) and CKP (*n* = 307). The solid horizontal line represents the median, the lower boundary represents the lower quartile, the upper border represents the upper quartile, and the whiskers represent outliers. Data are presented as mean ± SD. Statistical significance was determined using a two-tailed Student’s *t* test. **L** Dot plot depicting the expression profiles of anti-inflammatory and inflammatory genes in Mac #1, Mono #6, and Mono #7 subcultures from KP and CKP tumors, along with their enrichment patterns across subclusters.

To determine how CRACD depletion contributes to immune evasion, we used scRNA-seq to analyze KP and CKP tumors isolated from immunocompetent mice (Fig. 3A, B). We observed a significant enrichment of myeloid cells accompanied by a depletion of T cells in CKP compared with KP, highlighting distinct differences in the immune landscape between the two groups (Fig. 3B, C). In line with previous findings, CKP tumors expressed higher levels of MUC1 and MUC4 than KP tumor cells (Fig. 3D). Next, we analyzed cell-cell interactions using the CellChat package^40^. CKP tumors showed increased interactions mediated by PD-L1, an immune checkpoint molecule (Fig. 3E, Supplementary Fig. 6C). Because PD-L1 expression was significant in CKP tumors (Fig. 3F), PD-L1 may be a key factor driving immune evasion of CKP tumors.

To dissect the heterogeneous subpopulations of immune cells, we conducted a subclustering analysis of T cells and identified 12 distinct subclusters, each annotated based on the expression profiles of marker genes (Fig. 3G). Among these subclusters, the exhausted CD8^+^ T-cell subcluster in CKP showed marked upregulation of immune checkpoint genes, including *Pcdc1*, *Havcr2*, and *Lag3*, compared with KP (Fig. 3H). Analysis of four CD4^+^ T-cell subclusters also revealed enrichment of two regulatory subclusters in CKP, characterized by high expression of regulatory markers, including *Ctla4*, *Tnfrsf4*, *Tnfrsf18*, *Maf*, *Foxp3*, and *Icos* (Fig. 3I).

Subclustering analysis of myeloid cells identified 15 distinct subclusters (Fig. 3J). Among those subclusters, Mac #1, Mono #6, and Mono #7 were characterized by significantly higher M2 polarization scores than M1 scores, indicating their immunosuppressive phenotypes (Fig. 3K). Specifically, Mac #1 in CKP showed decreased expression of inflammatory genes, including *Ptgs2*, *Il1a*, *Ccl4*, and *Il1b*, compared with Mac#1 in KP (Fig. 3L). In CKP, Mono #6 and Mono #7 showed upregulation of anti-inflammatory genes, including *Vegfa*, *Mif*, *Cd274*, and *Lgals3*, along with downregulation of inflammatory genes (Fig. 3L). Notably, Mono #7, which was highly enriched in CKP cells, had the most pronounced anti-inflammatory profile, implicating the immunosuppressive nature of the CKP tumor microenvironment in this phenotype (Fig. 3L). These findings suggest that CRACD loss remodels the immune landscape, enabling immune evasion of tumor cells.

**Fig. 4:**
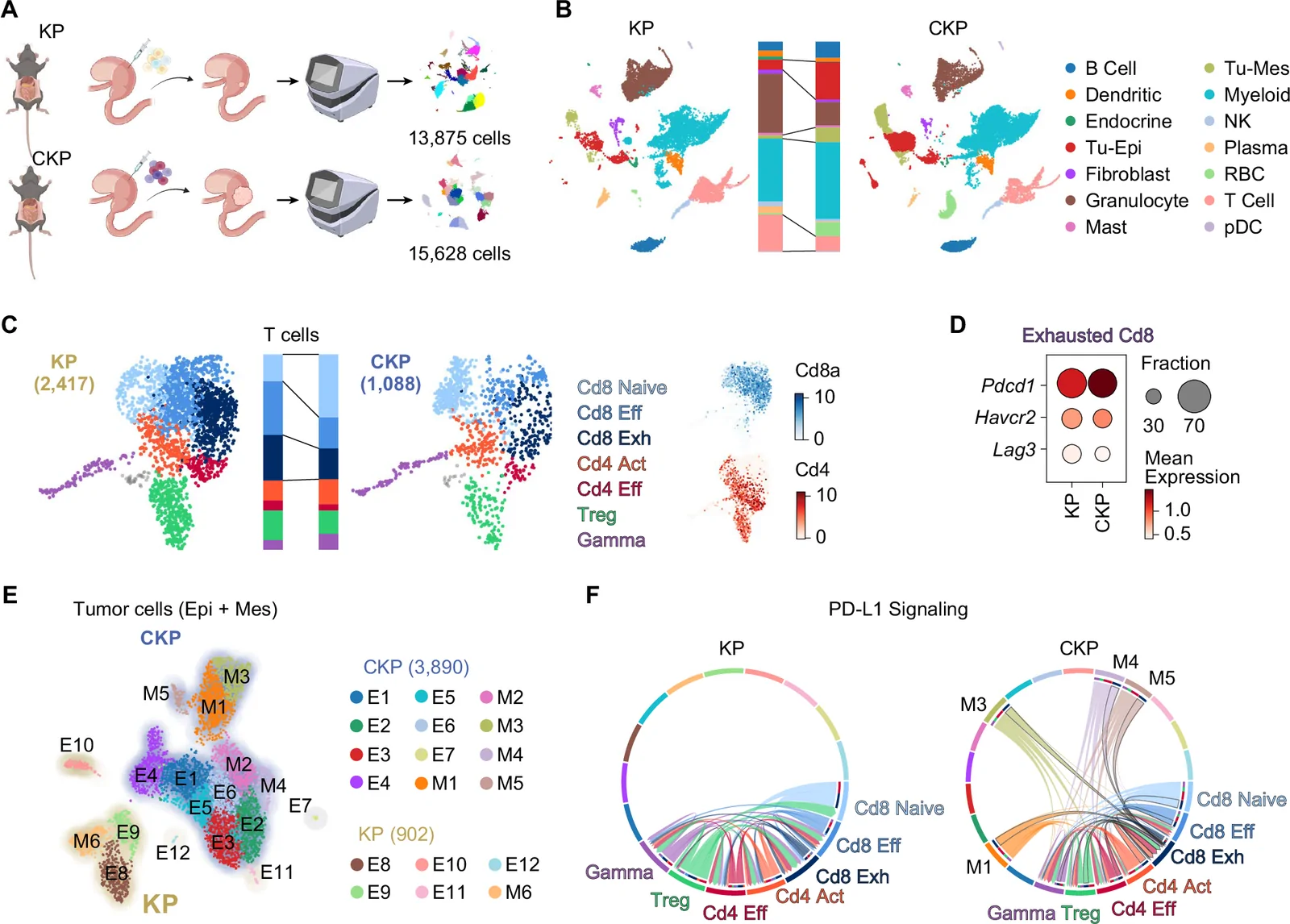
Orthotopic tumor single-cell transcriptomics reveals immune evasion and inflammatory signaling in CRACD-deficient gastric cancer. **A** Schematic of the workflow: tumors derived from orthotopic transplantation models were isolated and subjected to scRNA-seq. The schematic was created with BioRender.com. **B** UMAP visualization of scRNA-seq profiles, with bar plots indicating the relative proportions of annotated cell types. **C** Subclustering of T cells with UMAPs and bar plots showing subtype composition in KP and CKP; feature plots of *Cd4* and *Cd8a* on the right. **D** Dot plots of exhaustion markers (*Pdcd1, Havcr2, Lag3*) in exhausted CD8⁺ T cells from KP and CKP tumors. **E** Subclustering analysis of tumor epithelial and mesenchymal cells to resolve malignant cell heterogeneity. **F** Cellular interaction analysis of PD-L1 signaling between tumor clusters and T cell subsets, shown for KP (left) and CKP (right).

To complement immune profiling data from subcutaneous tumors, we also conducted scRNA-seq analysis of tumors derived from orthotopic gastric injections of KP and CKP cells (Fig. 4A). CKP orthotopic tumors also exhibited profound immune remodeling (Fig. 4B). In orthotopic models, CKP tumors grew substantially larger than KP tumors (Supplementary Fig. 6D). CKP tumors showed a higher proportion of exhausted CD8^+^ T cells compared with KP tumors, accompanied by marked upregulation of checkpoint molecules, including *Pdcd1*, *Havcr2*, and *Lag3*, further supporting an exhausted phenotype (Fig. 4C, D). In addition, cell-cell communication analysis demonstrated that PD-L1-mediated signaling was substantially more pronounced in CKP tumors than in KP tumors (Fig. 4E, F). Moreover, CD8^+^ T cells were abundant within and around KP tumors but largely absent in CKP tumors (Supplementary Fig. 6E, F). To further determine the role of exhausted CD8^+^ T cells in immune evasion of CKP tumors, we also conducted CD8^+^ T cell depletion followed by orthotopic injection of KP or CKP cells. Functional depletion of CD8^+^ T cells promoted KP tumor growth, whereas CKP tumors remained unaffected, validating that CRACD loss confers resistance to CD8^+^ T cell-mediated immunity (Supplementary Fig. 6G–J). These results suggest that CRACD loss not only exhibits increased tumorigenic capacity but also establishes an immunosuppressive microenvironment enriched in exhausted CD8^+^ T cells and PD-L1 signaling, thereby facilitating immune evasion.

### CRACD depletion-elevated ROS induces HIF1α-transactivated *PD-L1*

**Fig. 5:**
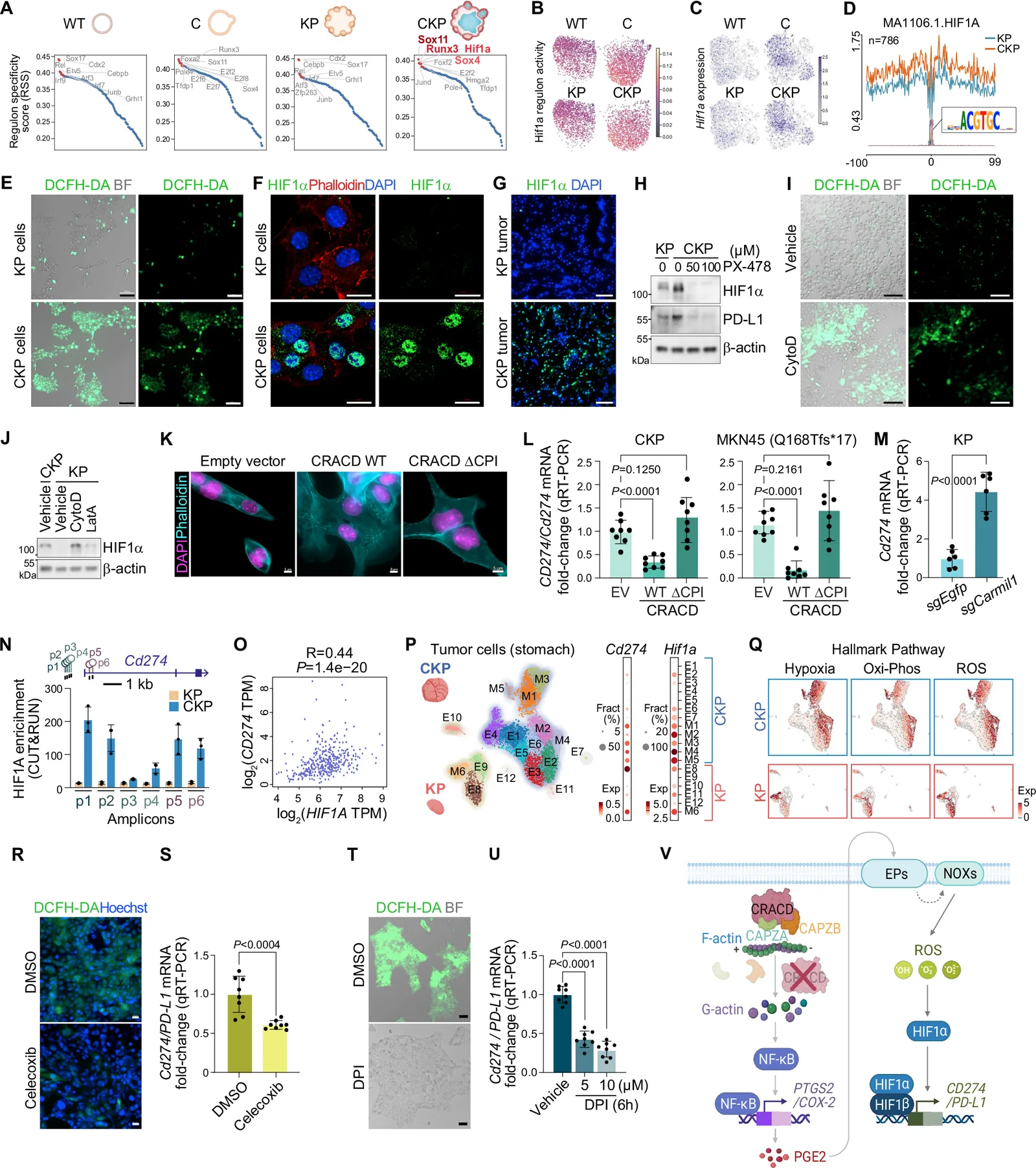
*PD-L1* transactivation by the ROS-HIF1α axis in CRACD-deficient tumor cells. **A** Regulon specificity scores in WT, C, KP, and CKP organoids; schematics created with BioRender.com. **B**,** C** *Hif1α*’s regulon activity (**B**) and mRNA expression (**C**). **D** Motif-predicted HIF1α-binding sites in CKP vs. KP. **E** 2′,7′-Dichlorodihydrofluorescein diacetate (DCFH-DA) imaging. Scale bars: 100 μm; BF, bright-field. **F**,** G** Immunofluorescence staining of KP and CKP cells (**F**) and tumors (**G**) for HIF1α. Scale bars: 20 μm (**F**) and 100 μm (**G**). **H** Immunoblot of PX-478-treated CKP cells. **I** DCFH-DA imaging of KP cells treated with cytochalasin D (CytoD; 100 nM, 48 h). Scale bars, 100 μm. **J** Immunoblot of KP cells treated with CytoD or latrunculin A (LatA; 100 nM, 48 h). **K** Immunofluorescence staining of phalloidin in CKP cells transduced with empty vector (EV), CRACD wild-type (WT), and ΔCPI mutant constructs; scale bars, 5 μm. **L** *Cd274*/PD-L1 mRNA in CKP and MKN45 cells expressing EV, WT or ΔCPI. **M** *Cd274*/PD-L1 mRNA in *Carmil1*-knockout KP cells. **N** HIF1α CUT&RUN-PCR in CKP cells (*n* = 3). **O** Pearson correlation of HIF1A and CD274 in TCGA-STAD samples (*n* = 408). **P** UMAP and dot plots of orthotopic tumors. Dot plots showing *Cd274* (left) and *Hif1α* (right) expression. **Q** Hallmark pathway activity in tumor cells from orthotopic tumors. UMAP plots showing enrichment scores for hypoxia, oxidative phosphorylation, and ROS pathways in CKP (top) and KP (bottom). **R**,** S** DCFH-DA imaging (**R**) and Cd274 mRNA (**S**) in celecoxib-treated CKP cells (10 μM, 48 h). Scale bars, 20 μm. **T**,** U** DCFH-DA imaging (T) and *Cd274* mRNA (U) in DPI-treated CKP cells (5 or 10 μM, 6 h). Scale bars, 50 μm. **V** Proposed models of CRACD-loss-induced ROS-HIF1α-PD-L1 signaling; created with BioRender.com and edited. Experiments were performed independently three times, each with triplicate wells (*n* = 3 biologically independent experiments). Dots denote independent experiments (L, S, and U, *n* = 8; M, *n* = 6). Data are mean ± SD; bars, mean; error bars, SD. Two-tailed Student’s *t* tests were used.

A growing body of evidence has implicated transcriptional reprogramming in cell plasticity^41^. This led us to identify critical transcriptional modules mediating *Cracd* loss-induced cell plasticity. We identified the gene regulatory network affected by CRACD depletion using scRNA-seq-based pySCENIC analysis^42^. The cell-regulon activity heatmap revealed distinct clusters of regulons corresponding to the specific organoid types. Clustering of regulon patterns in the UMAP space identified a dramatic shift in the gene regulatory network in *Cracd* KO cells (Fig. 5A, C [*Cracd* KO] vs. WT, CKP vs. KP). Assessment of regulon activities by Z score and regulon specificity scores both identified the activation of *Hif1a, Runx3, Sox11*, and *Sox4* regulons in *Cracd* KO (CKP) (Fig. 5A). Consistently, their expression levels (Supplementary Fig. 7A) and regulon activity levels were significantly upregulated (*P* < 0.05) in *Cracd* KO compared with WT (C vs. WT, CKP vs. KP) (Fig. 5B, C and Supplementary Fig. 7A). To complement the scRNA-seq-based regulon results, we assessed the effects of *Cracd* KO on chromatin accessibility in KP and CKP cells by using an Assay for Transposase-Accessible Chromatin with sequencing (ATAC-seq) (Fig. 5D and Supplementary Fig. 7B–E). Compared with KP control cells, CKP cells had more enriched DNA fragments binding to the transcriptional start site, increased transcription factor-binding loci, and augmented chromatin accessibility on proximal promoters ( < 1 kb) (Supplementary Fig. 7B, C). Specifically, the number of motif-predicted binding sites for Hif1a was the highest in CKP compared with KP (Fig. 5D), indicating that *Cracd* KO cells had higher *Hif1a* activity.

Next, we determined how CRACD loss activates the HIF1α regulon. Because HIF1α is mainly activated by ROS^43–45^, we hypothesized that actin disruption by CRACD depletion generates ROS for HIF1α activation. ROS of KP and CKP cells were visualized by 2’,7’-dichlorodihydrofluorescein diacetate (DCFH-DA) staining. Although KP cells rarely displayed ROS, CKP cells showed markedly elevated ROS (Fig. 5E). Similarly, unlike KP cells, CKP cells exhibited robust nuclear localization of HIF1α (Fig. 5F). In line with this, CKP allograft tumors displayed strong nuclear HIF1α, whereas KP barely expressed HIF1α protein (Fig. 5G). Several studies have shown that the HIF pathway upregulates PD-L1^46,47^. Given the distinct expression of PD-L1 in *Cracd* KO tumors (Figs. 3E, 4F), we tested whether HIF1α mediates PD-L1 expression by treating CKP cells with PX-478, an HIF1α inhibitor. PX-478 reduced ROS levels and concomitantly downregulated both HIF1α and PD-L1 (Fig. 5H), demonstrating that activation of the ROS–HIF1α axis is a key mechanism by which CRACD loss drives PD-L1 upregulation.

CRACD is a capping protein inhibitor that prevents actin polymerization at the barbed ends by binding to capping proteins (CAPZA or CAPZB)^30^. Thus, we also inquired whether CRACD inhibits PD-L1 expression via actin-dependent mechanisms. We tested whether dysfunction of actin polymerization increases ROS. Cytochalasin D (CytoD, an actin polymerization inhibitor) was found to escalate ROS in KP cells (Fig. 5I). Similarly, CytoD and Latrunculin A (LatA, another actin polymerization inhibitor) treatment increased HIF1α protein in KP cells (Fig. 5J), suggesting that actin dysregulation is sufficient to generate ROS and activate HIF1α. In addition, we performed rescue experiments by introducing CRACD WT or ΔCPI mutant (lacking capping-protein-interacting motifs) into CRACD-inactivated cells. In CKP cells, ectopic CRACD WT restored phalloidin-positive F-actin and reduced both ROS and PD-L1, whereas the ΔCPI mutant failed to rescue (Figs. 5K, L), indicating that CRACD’s capping-protein regulatory function is required for PD-L1 suppression (Supplementary Fig. 8A). Consistent with these findings, we further examined human gastric cancer cell line MKN45, which carries a non-sense mutation in the *CRACD* gene (Q168Tfs*17) (Supplementary Fig. 8B). Rescue experiments in MKN45 cells similarly showed that CRACD WT, but not the ΔCPI mutant, restored filamentous actin structures, reduced ROS and PD-L1 expression (Fig. 5L and Supplementary Fig. 8C). To further validate the impact of actin dysregulation on PD-L1 expression, we depleted another capping protein inhibitor, CARMIL1. In KP cells (*Cracd* WT), *Cramil1* KO induced F-actin disruption together with increased ROS and PD-L1 upregulation (Fig. 5M and Supplementary Fig. 8D, E), corroborating that actin dysregulation indeed enhances PD-L1 expression. In addition to the nonsense mutations, the *CRACD* gene carries missense mutations in the GC tumor cells. Similar to the ΔCPI mutant, the ectopic expression of various CRACD mutants (missense mutations) also failed to reconstitute organized F-actin in MKN45 cells, indicating impaired actin-regulatory capacity (Supplementary Fig. 9).

Next, we determined whether HIF1α directly transactivates *PD-L1* by performing Cleavage Under Targets and Release Using Nuclease (CUT&RUN) assays of KP and CKP cells. In CKP cells, HIF1α was enriched in the proximal promoters of the *Cd274* gene encoding the PD-L1 protein, relative to the KP cells (Fig. 5N). We further examined the pathological relevance of HIF1α and PD-L1 in human GC by analyzing TCGA datasets and found that *HIF1A* mRNA expression was correlated with *Cd274/PD-L1* transcriptional expression in GC (Fig. 5O). These data indicate that CRACD loss promotes ROS generation and PD-L1 expression through actin dysregulation.

To further delineate how actin dysregulation mechanistically activates the HIF pathway, we re-analyzed scRNA-seq datasets of KP and CKP orthotopic tumors (Fig. 5P). CKP tumor cells exhibited higher expression of *Cd274* and *Hif1a* compared with KP tumor cells (Fig. 5P). Moreover, hallmark pathway analysis revealed strong enrichment of hypoxia, oxidative phosphorylation, and ROS signatures in CKP tumor clusters (Fig. 5Q), recapitulating the results from organoids (Fig. 5A–D). Intriguingly, pathway analysis revealed marked upregulation of inflammation-related pathways (IL/STAT, inflammatory, TNF/NF-κB, and IFN-γ) in CKP tumors compared with KP (Supplementary Fig. 10A, B). Given that NF-κB mediates these pathways, we checked the expression of NF-κB in KP and CKP tumors. Immunostaining of orthotopic tumors displayed stronger NF-κB (p65) expression in CKP relative to KP (Supplementary Fig. 10C). It is noteworthy that inhibiting actin polymerization activates NF-κB in myelomonocytic cells^48,49^. NF-κB elevates intracellular ROS by the transactivation of genes encoding NADPH oxidases, xanthine oxidase, inducible nitric oxide synthase, cytochrome P450 enzyme, and oxygenases, including lipoxygenases and cyclooxygenases (COX-2)^43,50,51^.

Since CRACD loss activates the HIF–PD-L1 axis in a cell-autonomous manner (Fig. 5A–F, H–N), we first analyzed scRNA-seq datasets of organoids rather than tumors. Interestingly, the expression of the *Ptgs2* gene, which encodes the COX-2 protein, was specifically expressed in *Cracd* KO organoids (C and CKP) (Supplementary Fig. 10D) as well as CKP tumors (Supplementary Fig. 10E). Unlike typical oxidases, COX-2 indirectly increases ROS. COX-2 converts arachidonic acid into prostaglandin endoperoxide H2 (PGH2), which is changed into prostaglandin E2 (PGE2) by prostaglandin E2 synthase (PGES). Then, PGE2 binds to G-protein coupled receptors, EPs (prostaglandin E2 receptors), leading to activation of NADPH oxidases (NOXs) and subsequent elevation of intracellular ROS^52,53^.

*Cracd* KO-specific *Ptgs2* prompted us to test the effect of the COX-2–NOX2 axis on ROS levels and PD-L1 expression. In CKP cells, celecoxib, a COX-2 inhibitor, decreased both ROS levels and *Cd274/PD-L1* expression (Fig. 5R, S), as did diphenyleneiodonium (DPI), an NADPH oxidase inhibitor (Fig. 5T, U).

To further validate the source of ROS accumulation, we genetically perturbed *Nox1*, a major NADPH oxidase family member induced in CRACD-deficient cells. Consistent with the effects of pharmacologic inhibition, *Nox1* depletion significantly reduced ROS levels and attenuated downstream HIF1α and PD-L1 expression (Supplementary Fig. 10F–H). These findings provide genetic evidence that NOX1-dependent ROS production represents a major source of oxidative stress downstream of CRACD loss and contributes to activation of the HIF1α–PD-L1 signaling axis.

Together, these results suggest that the loss of CRACD disrupts F-actin organization, leading to activation of the NF-κB-COX-2 signaling, which in turn stimulates NOX-dependent ROS production. The resulting oxidative stress stabilizes HIF1α and induces *PD-L1* transactivation (Fig. 5V).

### HIF1α–PD-L1 axis blockade suppresses CKP tumorigenesis

**Fig. 6:**
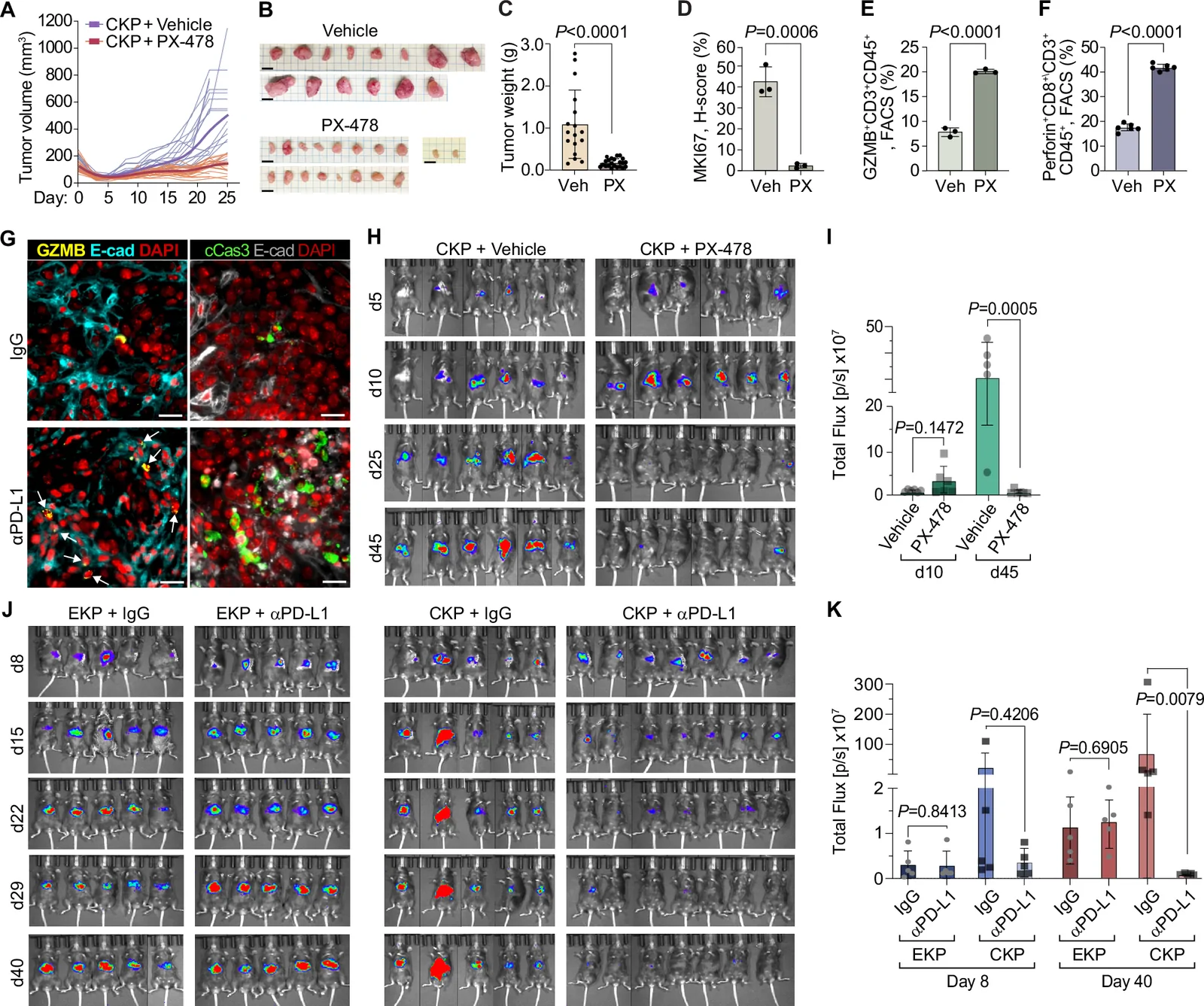
Suppression of *Cracd*^*-*/*-*^*Kras*^G12D^*Trp53*^*-*/*-*^ (CKP) tumorigenesis by PX-478 or anti-PD-L1 antibody. **A** Tumor growth curves of subcutaneously transplanted CKP cells treated with vehicle (Veh) or PX-478 (PX; 40 mg/kg, by intraperitoneal injection) every other day starting on day 7–10 after transplantation (Veh, *n* = 17 mice; PX, *n* = 22 mice). Light lines represent individual tumors, and dark lines represent the median values for each group. **B**,** C** Images (**B**) and weight (**C**) analysis of CKP tumors harvested from mice treated with vehicle or PX-478. Each dot represents one individual tumor. Data are presented as mean ± SD. Statistical significance was determined using a two-tailed Student’s *t* test. **D** Quantification of MKI67-positive cells. H-scores were quantified from three representative microscopic fields per tumor (*n* = 3 tumors per group). Each dot represents one individual tumor. Data are presented as mean ± SD. Statistical significance was determined using a two-tailed Student’s *t* test. **E**,** F** Fluorescence-activated cell sorting analysis of CKP tumors for GZMB^+^ CD3^+^ CD45^+^ (**E**) and Perforin^+^ CD8^+^ CD3^+^ CD45^+^ cells (**F**). Each dot represents one individual tumor. Data are presented as mean ± SD. Statistical significance was determined using a two-tailed Student’s *t* test. **G** Immunofluorescence staining of CKP tumors from mice treated with control IgG or anti-PD-L1 for GZMB, cleaved caspase-3 (cCas3), and E-cadherin (E-cad). Images are representative of three biologically independent tumors per group. **H** Bioluminescence images of C57BL/6 mice orthotopically transplanted with CKP and treated with Vehicle or PX-478. Tumor growth was monitored longitudinally using an in vivo imaging system (IVIS) at the indicated time points. **I** Quantification of total photon flux from orthotopic tumors in each group. Each dot represents one mouse. Data are presented as mean ± SEM, and statistical significance was determined by a two-tailed Mann-Whitney test. **J** Bioluminescence images of C57BL/6 mice orthotopically transplanted with EKP or CKP cells and treated with either control IgG or anti-PD-L1 antibody. Tumor growth was monitored longitudinally using an in vivo imaging system (IVIS) at the indicated time points. **K** Quantification of total photon flux from orthotopic tumors in each group. Each dot represents one mouse. Data are presented as mean ± SEM, and statistical significance was determined by a two-tailed Mann-Whitney test.

Having observed the immune evasion and PD-L1 enrichment in CKP tumor cells, we evaluated the therapeutic potential of targeting the HIF1α–PD-L1 axis in CKP tumors. We first examined whether PX-478 directly affects tumor cell growth in vitro. PX-478 did not elicit measurable growth inhibition of KP or CKP cells in vitro (Supplementary Fig. 11A), suggesting that its therapeutic effect may not be solely attributable to direct suppression of tumor cell proliferation. We next tested the anti-tumorigenic effect of PX-478 in vivo using immunocompetent mice transplanted with CKP cells. Compared with vehicle treatment, PX-478 significantly inhibited the in vivo tumorigenesis of CKP cells (Fig. 6A–C). Consistently, immunostaining confirmed that PX-478 markedly reduced HIF1α-positive tumor cells in CKP tumors, validating effective inhibition of the HIF1α pathway in vivo (Supplementary Fig. 11B, C). PX-478 treatment also led to decreased MKI67 expression and a substantial increase in cleaved caspase-3 (cCAS3) (Fig. 6D and Supplementary Fig. 11E, F).

Given that scRNA-seq analysis revealed reduced T cell populations in CKP tumors compared with KP tumors (Fig. 4), we further assessed whether PX-478 treatment restores anti-tumor immune activity. Fluorescence-activated cell sorting analysis of tumor-infiltrating immune cells revealed marked increases in effector T-cell populations, including Granzyme B [GZMB]- or Perforin-positive T cells, in PX-478-treated CKP tumors compared with vehicle-treated controls (Fig. 6E, F and Supplementary Fig. 11G–J).

Based on our finding that HIF1α transactivated *PD-L1* in CKP tumors (Fig. 5), we also tested whether anti-PD-L1 antibody (αPD-L1) treatment could suppress CKP tumors. Similar to PX-478, administration of anti-PD-L1 antibody markedly inhibited CKP tumorigenesis compared with IgG isotype controls (Supplementary Fig. 12A, B), along with a notable increase in effector T-cell populations and tumor cell death (Fig. 6G and Supplementary Fig. 12C, D).

We extended these findings in more pathologically relevant orthotopic models. Mice orthotopically injected with CKP cells were administered with PX-478 or αPD-L1. Bioluminescence imaging showed that both PX-478 and αPD-L1 treatment significantly reduced tumor burden compared with the control^54^ (Fig. 6H–K). We also included the EKP model (*Cdh1/E-cadherin* KO; *Kras*^G12D^; *Trp53* KO)^55^, which represents an E-cadherin-deficient gastric cancer subtype enriched with exhausted T cells but lacking robust PD-L1 expression (Supplementary Fig. 12E). EKP tumors served as a negative control and did not respond to αPD-L1 treatment, whereas CKP tumors showed sensitivity (Fig. 6J, K). These results strongly suggest that CRACD loss-associated PD-L1 upregulation underlies the therapeutic vulnerability of CKP tumors to inhibition of the HIF1α–PD-L1 axis.

### Clinical validation between CRACD downregulation and PD-L1 upregulation

**Fig. 7:**
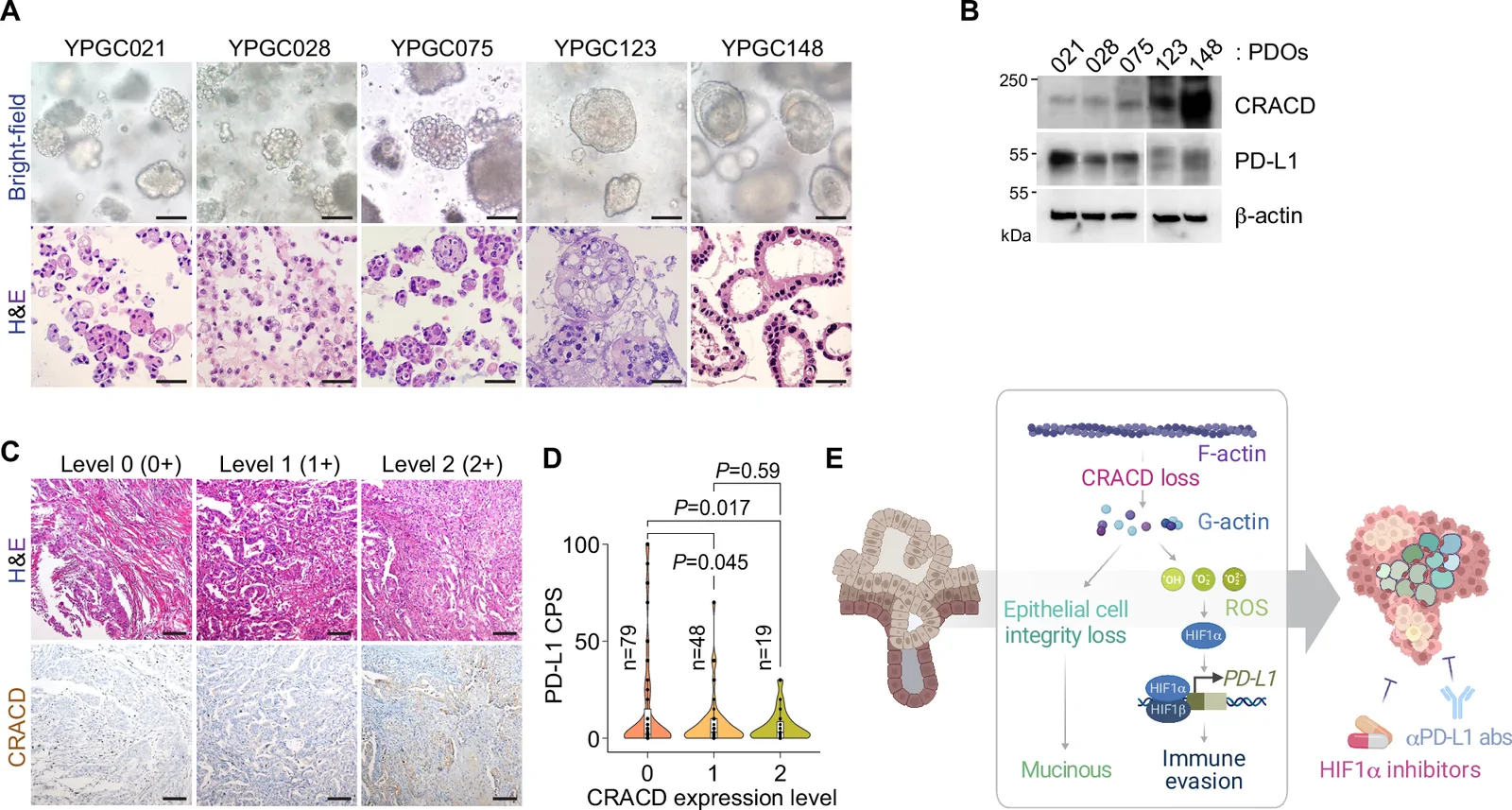
CRACD expression in human GC tissues and its association with PD-L1. **A** Representative brightfield and H&E images of PDOs established. Scale bars: 50 μm. Representative bright-field and H&E images of five PDOs are shown. **B** Immunoblot analysis of CRACD and PD-L1 expression in PDOs. **C** Representative H&E (top) and CRACD IHC (bottom) images of human GC tissues stratified into three groups according to CRACD expression: Level 0 (negative), Level 1 (1 + , low), Level 2 (2 + , high). Scale bars, 100 µm. **D** Violin plots showing PD-L1 combined positive score (CPS) according to CRACD expression levels: 0 (*n* = 79), 1 (*n* = 48), and 2 (*n* = 19). Each dot represents an individual tumor sample. The solid horizontal line represents the median, the lower boundary represents the lower quartile, the upper border represents the upper quartile, and the whiskers represent outliers. Data are presented as mean ± SEM, and statistical significance was determined by Student’s *t* test. **E** Illustration of gastric tumor cell plasticity and immune evasion induced by CRACD inactivation. CRACD loss of function induces cell plasticity, converting intestinal-type gastric tumors into mucinous diffuse gastric tumors. CRACD loss disrupts actin polymerization, which generates ROS. Subsequently, ROS-activated HIF1α transactivates *PD-L1* to allow immune evasion by tumor cells, which could be targeted by HIF1α inhibitors, such as PX-478 or anti-PD-L1 immune checkpoint inhibitors (atezolizumab, pembrolizumab, or duravalumab). The schematic illustration in panel E was created with BioRender.com and edited.

To validate our findings clinically, we examined gastric cancer patient-derived organoids (PDOs) and observed an inverse relationship between CRACD and PD-L1 expression (Fig. 7A, B). We also extended this analysis to a gastric cancer tissue microarray^56^ comprising 182 patient samples. (Fig. 7C). Stratifying tumors based on CRACD expression showed that cases with CRACD loss (score 0) exhibited significantly higher PD-L1 CPS scores (Fig. 7D). These results confirm a clinical link between CRACD inactivation and PD-L1 upregulation in human GC.

## Discussion

MGC is associated with aggressive clinical behavior, chemoresistance, and poor outcomes. Patients with MGC frequently derive limited benefit from immune checkpoint blockade, underscoring the urgent need for biomarkers that guide immunotherapy selection. Our study revealed that CRACD is frequently inactivated in gastric cancer and acts as a driver of mucinous transformation and immune evasion. Genetic ablation of *CRACD* induced hypermucinosis, disrupted epithelial integrity, and enriched PD-L1 expression in preclinical models. These findings were clinically validated by PDOs and TMA, where CRACD-low tumors exhibited significantly higher PD-L1 CPS scores.

Mechanistically, we delineated a stepwise signaling cascade whereby CRACD loss disrupted actin polymerization, leading to NF-κB/COX2 pathway activation and PGE2 induction, which subsequently stimulated NADPH oxidase-dependent ROS generation. Elevated ROS stabilized HIF1α, resulting in transactivation of *CD274/PD-L1* and suppression of CD8⁺ T cell-mediated immune surveillance. This integrated pathway was supported by scRNA-seq of orthotopic tumors, which revealed enrichment of NF-κB/COX-2-related transcriptional signatures (Fig. 5Q and Supplementary Fig. 8G). Notably, pharmacologic inhibition of HIF1α or PD-L1 restored immune surveillance and suppressed tumor progression, highlighting actionable vulnerabilities in CRACD-inactivated gastric cancer.

Beyond immune evasion, CRACD loss also conferred proliferative and angiogenic advantages, promoting metastasis. In *Cracd* KO syngeneic and orthotopic models, we observed increased tumor proliferation, PD-L1-mediated immunosuppression, and enhanced angiogenesis marked by the upregulation of vascular endothelial growth factor (*VEGF*) and diffuse infiltrative growth into the gastric wall and muscular layer (Fig. 3L). Treatment with an HIF1α inhibitor effectively decreased these angiogenic and infiltrative features, supporting HIF1α‘s role as a transcriptional activator of *VEGF*. In addition, in orthotopic models, CKP tumors often developed metastases, reflecting their invasive and angiogenic phenotype (Fig. 2E–G and Supplementary Fig. 5A, B). Importantly, treatment with an HIF1α inhibitor or anti-PD-L1 antibody not only reduced primary tumor growth but also limited metastatic spread, underscoring its potential as a therapy for CRACD-inactivated GC.

In addition, CD8⁺ T cell depletion experiments showed contrasting immune dependencies: depleting CD8^+^ T cells accelerated tumor growth in KP models but had minimal impact on CKP tumors (Supplementary Fig. 6G–J), indicating that CRACD-deficient tumors are intrinsically resistant to CD8⁺ T cell-mediated immune control. These data demonstrate that CRACD loss not only promotes angiogenesis and infiltration via HIF1α-VEGF signaling but also establishes a PD-L1-driven immune-evasive phenotype that renders tumors refractory to T cell depletion, highlighting dual vulnerabilities-angiogenesis and immune evasion that can be therapeutically targeted in CRACD-inactivated gastric cancer.

Clinically, mucinous GC remains an under-recognized yet challenging subtype^8^. Despite frequent PD-L1 expression, MGC patients often fail to achieve durable responses to immunotherapy, indicating that PD-L1 alone is insufficient as a predictive biomarker. Our results identify CRACD status as an additional stratification marker that may capture the unique immunobiological features of this subtype. However, we do not propose CRACD as a replacement for established immunotherapy biomarkers such as PD-L1 CPS, MSI status, or EBV status. Rather, CRACD loss may represent a complementary biomarker candidate whose independent or additive predictive value should be tested in larger clinically annotated cohorts treated with PD-1/PD-L1 blockade.

Notably, our findings suggest that CRACD loss may function beyond a subtype-specific alteration and instead promote a biologic state characterized by increased cellular plasticity, immune evasion, angiogenesis, and metastatic potential. Consistent with previous studies linking CRACD deficiency to lineage reprogramming in other cancer contexts, CRACD loss may weaken epithelial lineage constraints and facilitate adoption of alternative cellular states.

From a translational perspective, these findings support the development of biomarker-guided therapeutic strategies. CRACD-low GC patients might be ideal candidates for combined blockade of the HIF1α–PD-L1 axis, potentially in conjunction with anti-angiogenic therapy. In addition, given the strong metastatic propensity of CRACD-deficient tumors, strategies incorporating HIF1α or PD-L1 inhibitors in the metastatic setting may hold promise. Notably, long-term follow-up revealed tumor recurrence following treatment withdrawal in both anti-PD-L1 and PX-478-treated cohorts. Late liver metastasis emerged after prolonged withdrawal of PX-478 treatment. These findings suggest that although targeting either HIF1α or PD-L1 alone effectively suppresses primary tumor growth, durable disease control may require sustained treatment or combination therapy.

Although our murine models and organoid systems faithfully recapitulate mucinous GC features, translation to human pathology requires validation in larger and independent patient cohorts. The incorporation of CRACD expression analysis into ongoing immunotherapy trials will be essential for assessing its predictive value. Future studies should also explore whether CRACD loss directly contributes to metastatic dissemination pathways and if CRACD testing can be practically implemented as a biomarker in routine diagnostics. F-actin inhibition was shown to activate the NF-κB^48,49^, which is in line with our results. Dissecting the molecular mechanisms by which actin disruption activates NF-κB warrants further studies.

Taken together, our findings unveil that CRACD loss is a key driver of mucinous differentiation, immune evasion, and metastasis in gastric cancer. These results define a CRACD-low mucinous GC as a distinct immunobiological subtype with a poor prognosis and limited response to standard therapies, yet potentially vulnerable to targeted inhibition of the HIF1α–PD-L1 axis. By integrating mechanistic insight with clinical validation, our study provides a strong rationale for further biomarker-integrated therapeutic studies in CRACD-deficient gastric cancer.

## Materials and methods

### Ethics statement

All animal procedures were approved by the Institutional Animal Care and Use Committee of The University of Texas MD Anderson Cancer Center (protocol no. 00002587-RN00) and were performed in accordance with institutional and Assessment and Accreditation of Laboratory Animal Care (AAALAC) international guidelines. Tumors were not permitted to exceed 1.5 cm in diameter or 20% of the animal’s body weight, and neither limit was exceeded in any experiment.

The collection and research use of human gastric cancer specimens, including ascites-derived patient-derived organoids and tumor specimens used to construct the in-house tissue microarray, were approved by the Institutional Review Board of Yonsei University Hospital, Seoul, Republic of Korea (IRB no. 4-2014-0638). Written informed consent for specimen collection and research use was obtained from all participants before inclusion in the study. The study was conducted in accordance with the Declaration of Helsinki. A commercially available gastric cancer tissue microarray (ST821) was purchased from US Biomax.

### Subcutaneous syngeneic models

Male and female C57BL/6 mice (RRID: IMSR_JAX:000664; The Jackson Laboratory, Bar Harbor, ME, USA), 6–8 weeks of age, were housed in the MD Anderson Division of Laboratory Animal Resources under a 12 h light/12 h dark cycle with controlled temperature and humidity and had ad libitum access to food and water. Mice were randomly assigned to experimental groups. For subcutaneous tumor implantation, 3 × 10⁶ KP or CKP cells were injected into each flank. Starting 7–10 days after tumor implantation, mice were treated with either PX-478 (40 mg/kg, intraperitoneally, every other day for 2-3 weeks) or anti-PD-L1 antibody (10 mg/kg, intraperitoneally, twice weekly for 4 weeks). Vehicle and isotype-matched IgG were used as the respective controls. Tumor dimensions were measured with calipers every 2 days, and tumor volume was calculated as (length × width²)/2. At the indicated experimental endpoints, mice were euthanized, and tumors were photographed, excised, weighed, and processed for paraffin embedding and subsequent immunostaining or scRNA-seq, as indicated.

### Gastric orthotopic injection

Male and female C57BL/6 mice (6–8 weeks of age) were used for orthotopic transplantation. To evaluate orthotopic tumor growth of KP and CKP cells, we first generated luciferase-expressing cell lines by transducing them with pLenti-PGK-Venus-AkaLuc(neo) lentivirus. A total of 1 × 10^6^ cells were suspended in 50 μl of 50% Matrigel/phosphate-buffered saline (PBS) and injected into the distal gastric wall of C57BL/6 mice (6–8 weeks). This orthotopic injection technique was optimized to minimize leakage into the peritoneal cavity and thereby reduce false-positive signals. Tumor growth was monitored weekly by in vivo bioluminescence (BLI) imaging using the IVIS Lumina III system. For imaging, mice were injected intraperitoneally with premixed AkaLumine-HCl at the doses used in tumor studies, 10 min prior to image acquisition. Peritoneal carcinomatosis and primary tumor sites were monitored longitudinally by BLI.

### Cell culture

2D organoid-derived gastric cancer cell lines (KP and CKP) and established gastric cancer cell lines SNU-1 (ATCC, RRID:CVCL_0099), SNU-5 (ATCC, RRID:CVCL_1717), SNU-16 (ATCC, RRID:CVCL_0076), and NCI-N87 (ATCC, RRID:CVCL_1603) were maintained in RPMI-1640 + 10 % FBS + 1 % PS at 37 °C in a humidified incubator supplied with 5% CO_2_, as we previously performed^55^. MKN45 cells were kindly provided by Dr. Pawel Mazur, and AGS and KATOIII cells were obtained from Dr. Xiangsheng Zuo (MD Anderson Cancer Center).

### Isolation of cells from ascites of advanced gastric cancer patients

Ascites was collected from patients diagnosed with advanced-stage gastric cancer. The volume of ascites varied between patients. However, to standardize the experimental protocol, only 200 mL of ascites was used to collect cells. Contaminating red blood cells in the cell pellet of ascites were removed by hypotonic lysis in sterile MilliQ H_2_O and added ADF medium and was strained over a 100 μm filter with retained debris. After centrifugation at 500 rcf, the supernatant was removed. Cell pellets were counted under a microscope and centrifuged (500 rcf, 5 min). Isolated cells were resuspended in ice-cold 500 μl matrigel and 20 μl drops of matrigel- cell suspension were seed in pre-warmed 48-well culture plate at a density of ~ 2 × 10^3^ per 20 μL matrigel/well. The matrigel was solidified for 10 min 37 °C and overlaid with 250 μL warm expansion medium (ADF, 50% conditioned Wnt3A-medium, 10 % conditioned R-spondin1 medium supplemented with B27, 1 mM N-Acetylcystein, 10 mM nicotinamide, 50 ng/mL human epidermal growth factor, 100 ng/mL human noggin, 200 ng/mL human fibroblast growth factor, 1 nM gastrin, 2 μM A83-01). 10 μM Y-27632 was added for the first 2days. Culture was kept at 37 °C, 5 % CO2 in a humidified incubator. For the clinical information, see Supplementary Dataset 4.

### Maintenance of gastric cancer organoids

Culture medium was exchanged every 2-3 days, and organoids were passaged every 10–15 days at a ratio of 1:4–1:8. For passaging, culture medium was removed, and organoids together with matrigel. After transfer to a new 1.7 mL tube, organoids were mechanically dissociated by vigorous pipetting. Next, the dissociated organoids were centrifuged at 500 rcf, 5 minutes (min), and the pellets incubated with TrypLE Express Enzyme (37 °C, 2-3 min). TrypLE was inactivated by the addition of ADF, and the sheared organoids were pelleted as above and centrifuged. Surprenant was discarded, the pellet resuspended in ice-matrigel, and re-seeded as described above.

### Drugs and chemical testing on cell lines

PX-478 (Selleckchem, S7612) was obtained commercially. To determine GI_50_ values, cells were digested with Trypsin-EDTA (Corning, #25-052-CI) to prepare a single-cell suspension. The suspension was diluted to a concentration of 30,000 cells per ml, and 100 μl of this suspension was seeded into each well of a 96-well plate, leaving the last row blank without cells. After 24 h (h) of incubation, 200 μl of medium containing drugs at gradient concentrations was added to each well, with triplicates for each concentration. Following a 72 h incubation period, the drug-containing medium was replaced with 100 μl of fresh medium containing 5 μl of CCK-8 reagent from the Cell Counting Kit-8 (Dojindo, CK0413) per well. The plates were incubated at 37 °C for an additional 4 hours, and absorbance at 450 nm was measured using a microplate reader. For analysis, absorbance values from the blank wells were used as 0 % viability, while those from drug-free wells were set as 100 % viability. GI50 values were calculated using GraphPad Prism. Latrunculin A (Cayman, 10010630) and Cytochalasin D (MCE, HY-N6682) were used to inhibit actin polymerization. Diphenyleneiodonium chloride (MCE, HY-100965), N-Acetylcysteine amide (MCE, HY-110256), and mitoTEMPO (MCE, HY-112879) were utilized to inhibit reactive oxygen species (ROS). Celecoxib (MCE, HY-14398) was used to inhibit COX-2.

### CRISPR/Cas9 gene knock-out

Gene knock-out (KO) was performed by the CRISPR/Cas9 genome editing system. Single guide RNAs (sgRNAs) targeting each gene were designed by Shalem et al.^57^ or using CRISPick (portals.broadinstitute.org/gppx/crispick/public) and cloned into a lentiCRISPR v2 plasmid (Addgene, #52961) following Zhang’s protocol^57^. sg*EGFP* targeting the gene encoding EGFP fluorescent protein was used as a negative control^57^. sgRNA sequences used in this study are listed below: *Carmil1* Mouse sg#1 TTTGTTTTTGCAGATGGTTG, Nox1 Mouse sg#1 GAAGACGAGGTACTTACTTG, HIF1a Mouse sg#1 GCAACCTCTTGATTCAGTGC, HIF1a Mouse sg#2 TAGATCATGGTCACAACCTC, Epas1 Mouse sg#1 CGATAAGTACCCGACGACGT.

### Virus production and transduction

HEK293T cells in a 10-cm dish were co-transfected with 5 μg of constructs, 5 μg of plasmid D8.2 (Plasmid #8455, Addgene), and 3 μg of plasmid VSVG (Plasmid #8454, Addgene). Cells were incubated at 37 °C, and the medium was replaced after 12 h. Virus-containing medium was collected 48 h after transfection and supplemented with 8 μg/mL polybrene to infect target cells in 6-well dishes. After 6 h, the medium was changed. After 48 h, the infected cells were selected with 2 μg/mL puromycin.

### Plasmids

Cells were transfected with pcDNA3.1-Flag-CRACD WT, ΔCPI mutant, or empty vector^58^ using FuGENE6 (Promega).

### CUT&RUN assays

CUTANA ChIC/CUT&RUN kit (EpiCypher, Cat. No. 14-1048) was used based on the manufacturer’s protocols. CUT&RUN was performed using *HIF1a* antibodies (Cell Signaling, 14179 and 36169). For controls, IgG and H3K4me3 antibodies provided in the kit were used.

### PCR and qRT-PCR

CUT&RUN was performed using *HIF1a* antibodies, and the resulting DNA was used to evaluate the binding of *HIF1a* to *Cd274*. *Cd274* primers (p1 F: 5’-TGAAGT GTCTGG ATTCTG AAG-3’, R: 5’-TTAGGG TGACCT TTGGGATA-3’; p2 F: 5’-GAGGAA GTCACC AAATCCAC-3’, R: 5’-TCTTGA AAGCCC TTTCTGGA-3’; p3 F: 5-ATCCAC GTATCC AGAAAGGG-3’, R: 5’-TCGGTG GTGGTG ACTACTG-3’; p4 F: 5’-TCTTAT GACTTCAGATAT TTTGCT-3’, R: 5’-AGGAGC AGTGAGCGC TTTAG-3’; p5 F: 5’-ATGACTGGGTCTTTCCACTT-3’, R: 5’-GAGGTCTAGGATGCTGGAGC-3’; p6 F: AAACAGTTCTTAGAT ACAGTG-3’, R: 5’-GGGTTGTCATAAGAGGTGAAA-3’) were designed using the VISTA browser and used for PCR (Genedepot, PCR master mix) under the manufacturer’s recommended conditions. qRT-PCR was subsequently performed, following the methods described earlier.

### Immunoblotting

Cell lysates were prepared by incubating the cell plate or the mashed tumor tissues with RIPA lysis buffer (50 mM Tris-HCl, pH 7.5, 150 mM NaCl, 1.5 mM EDTA, 1% NP-40, 0.5 % Na-deoxycholate, 0.1% SDS) for 30 min, followed by full-speed centrifugation for 10 min at 4 °C. The protein concentration of the supernatant was calculated with a Pierce BCA protein assay kit (Thermo Fisher, #23227). The protein concentrations of loading samples were balanced with 5´ SDS loading buffer (2.5 mL 1 M Tris-HCl pH 6.8, 1 mL water, 1 g SDS powder, 5 mL glycerol, 0.5 mL β-mercaptoethanol, 0.5 % bromophenol blue) and RIPA buffer, followed by denaturation at 95 °C for 5 min. Then, the samples were loaded for SDS-PAGE. After the electrophoresis, the gel was transferred to the nitrocellulose membrane. The membrane was blocked with 1 % BSA in TBST (6.06 g Tris base powder, 8.76 g NaCl, 0.5 mL Tween-20 in 1 L water, pH 7.6) for 30 min at room temperature. Then, it was incubated with the primary antibody diluted in 1 % BSA in TBST overnight at 4 °C. Three times of washes with TBST were conducted. The HRP-conjugated secondary antibody was incubated with the membrane in TBST for 1 h at room temperature. Three times of washes with TBST were conducted. Enhanced chemiluminescence was performed with SuperSignal West Pico PLUS (Thermo Fisher, #34577) or SuperSignal West Femto Maximum Sensitivity Substrate (Thermo Fisher, #34095) reagent. For the antibody information, see Supplementary Dataset 1.

### Histology, immunohistochemistry, and immunofluorescence staining

The GC tumor microarray was purchased from Biomax (ST821). The murine and human tissue/tumor samples were fixed with 10% formalin, followed by paraffin embedding to make FFPE (formalin-fixed paraffin-embedding) blocks. The FFPE samples were immuno-stained following the standard protocol: deparaffinization, antigen retrieval, blocking, and primary antibody incubation. For immunohistochemistry (IHC) staining, HRP-conjugated secondary antibody was used. 3,3’-Diaminobenzidine (DAB) staining was performed with the ImmPACT DAB kit (Vector, SK-4105). Nuclei were counterstained with Hematoxylin QS (Vector, H-3404-100). For immunofluorescence (IF), fluorescence-conjugated secondary antibodies were used. For IF of cells, cells grown on coverslips were fixed in 4% paraformaldehyde, washed in PBS, and mounted with ProLong Gold Antifade Mountant with DAPI (Thermo Fisher, P36935). Imaging was performed under a fluorescence microscope. For the antibody information, see Supplementary Dataset 1. Additional details about TMA are provided in Supplementary Dataset 2.

### In-house tissue microarray construction and Immunohistochemistry

To construct the tissue microarray block, two cores were extracted from a representative tumor area of each case (3-mm diameter). The 4 μm thick sections from each TMA block were prepared for IHC. Hematoxylin and eosin and cytokeratin IHC staining were performed to confirm the presence of tumor cells. The IHC for cytokeratin (AE1/AE3) (1:300; DAKO, Carpinteria, CA, USA).

Tissue sections were cut and placed on slides. Using the Benchmark XT automated immunohistochemistry stainer (Ventana Medical Systems, Inc., Tucson, AZ, USA), slides were stained as follows: Detection was done using the *ultra*View Universal DAB Detection Kit and *ultra*View Universal Alkaline Phosphatase Red Detection Kit (Ventana Medical Systems). Sections were deparaffinized using EZ Prep solution. CC1 standard (9.0 Tris buffer) was used for antigen retrieval. DAB Inhibitor (3% Hydrogen Peroxide Solution) was dispensed and incubated for 4 min at 37 °C. For DAB staining, the Primary antibody was applied and incubated for 32 min, and the HRP Multimer for 8 min at 37 °C. Slides were incubated for 8 min after DAB Chromogen, and DAB H_2_O_2_ was dispensed simultaneously, followed by Cooper sulfate incubation for 4 min. Before the red stain step, slides are incubated at 90 °C for 4 min. For Red staining, the Double Stain Primary antibody was applied and incubated for 36 min, the AP Red Multimer for 12 min, and the Red Enhancer for 4 min at 37 °C. Slides were incubated for 8 min after Fast Red A and Red Naphthol were dispensed simultaneously. Fast Red B was incubated for 8 min, followed by hematoxylin and bluing reagent counterstain at 37 °C. Reaction buffer (pH 7.6 Tris buffer) was used as the washing solution. For the antibody information, see Supplementary Dataset 1. Additional details about TMA are provided in Supplementary Dataset 3.

### AB-PAS and HE staining

Deparaffinized tissue/tumor samples or paraformaldehyde (Fisher, #30525-89-4) fixed cells were incubated for 15 min with alcian blue (Sigma-Aldrich, A5268) solution (pH 2.5), followed by periodic acid-Schiff (PAS) staining (Sigma-Aldrich, 395B) according to the manufacturer’s protocol. The nuclei were counterstained with Hematoxylin QS Counterstain solution or Nuclear Fast Red Counterstain solution (Vector, H-3403-500). For hematoxylin and eosin (HE) staining, the standard procedure was employed using the HE staining kit (Abcam, ab245880).

### PD-L1 staining

PD-L1 IHC was performed on FFPE sections using the Dako Omnis automated staining platform (Agilent) with the EnVision FLEX detection system (Low pH). Sections underwent deparaffinization and antigen retrieval (Low pH buffer, 50 min), followed by peroxidase inhibition. Primary antibody incubation was carried out for 30 min at 32 °C using the Omnis instrument. Hematoxylin was applied for nuclear counterstaining (4 min).

### ROS detection

Cells were stained using the DCFDA - Cellular ROS Assay Kit (Abcam, ab113851) following the manufacturer’s protocol. Briefly, cells were seeded in a 48-well plate and allowed to adhere overnight. The culture medium was removed, and the cells were washed with 1 × PBS. Next, the cells were incubated with a working solution of 25 µM DCFDA in serum-free medium for 30 minutes at 37 °C in the dark. After incubation, the cells were washed with 1 × PBS, and fluorescence was measured using a microplate reader at excitation/emission wavelengths of 485 nm/535 nm to quantify ROS levels. For nuclear counterstaining, Hoechst 33342 (Invitrogen, H1399) was applied prior to imaging.

### CD8^+^ T cell depletion

CD8^+^ T cell depletion was achieved by intraperitoneal injection of 300 μg of anti-CD8 monoclonal antibody (clone 53-6.7, BioxCell) administered twice weekly on days – 2, 0, and 2, with day 0 defined as the day of orthotopic tumor implantation. Mice were euthanized on day 26 after tumor injection.

### Flow cytometry

Tumors from syngeneic models were collected and processed into single-cell suspensions for flow cytometry analysis. Tumor tissues were minced with a blade and transferred into a digestion solution containing collagenase A and DNase I (Sigma). The tissue suspension was incubated at 37 °C for 30 min to facilitate enzymatic digestion. After incubation, the cell suspension was filtered through a 70 μm cell strainer (Falcon) and washed twice with PBS. The cells were then filtered through a FACS tube strainer (Falcon) and washed twice more with FACS buffer (PBS containing 0.5% BSA and 2 mM EDTA). For surface staining, the following antibodies were used: PE anti-mouse CD45 (BioLegend, 1:100 dilution), Pacific Blue anti-mouse CD4 (BioLegend, 1:100 dilution), FITC anti-mouse CD3 (BioLegend, 1:50 dilution), and APC anti-mouse CD8 (BioLegend, 1:50 dilution). Cells were incubated with these antibodies for 30 min at 4 °C in the dark. After staining, cells were washed twice with FACS buffer. For intracellular staining of perforin and Granzyme B, cells were fixed and permeabilized using the Fixation Solution (BioLegend)/Permeabilization Solution (3% Triton-X100 in FACS buffer). Briefly, cells were fixed in fixation buffer for 20 min at 4 °C, followed by washing with Perm/Wash buffer. Cells were then incubated with Pacific Blue anti-mouse perforin (BioLegend, 1:50 dilution) or Pacific Blue anti-mouse Granzyme B (BioLegend, 1:50 dilution) in Perm/Wash buffer for 1 hour at 4 °C in the dark. After staining, cells were washed twice with Perm/Wash buffer and resuspended in FACS buffer. Flow cytometry was performed using an Attune flow cytometer, and data analysis was conducted with the FlowJo software (RRID:SCR_008520).

### Single-cell isolation of tumors

Allograft tumors or orthotopic tumors from mice (KP and CKP) were collected. The method for single-cell isolation of tumors was modified from the previous study^55^. Tumors collected from mice were transferred to a 1.5 mL tube and dissected into small pieces (smaller than 2 mm) with scissors. Tumor pieces were then incubated with 1 mL tumor digestion buffer (2.5 % FBS, 1 mg/mL Collagenase Type IV [STEMCELL, #07436], 250 μg/mL Dispase II [Gibco, #17105041], and 10 μM Y-27632 in advanced DMEM/F12 [advDF] medium [Gibco, #12634010]) at 37 °C in a water bath for 30 min. After incubation, the tumor pieces were completely dissociated by robust pipetting with a p1000 pipette, followed by a resuspension with 4 mL cold PBS. Then, the digested mixture was passed through a 100 μm cell strainer to enrich single cells. The filtrate was pelleted by swing-bucket centrifugation at 300 g for 3 min at 4 °C. Cells were further washed and pelleted twice by cold PBS. The supernatant was discarded. For FACS, the single-cell pellet was resuspended in cold 5% FBS-PBS. For organoid seeding, the pellet was resuspended in an appropriate volume of cold advDF plus medium. Then, the cell suspension was processed immediately for library preparation.

### Library construction

scRNA-seq libraries were prepared using the CellPlex kit and the 10X Genomics Chromium Single Cell Gene Expression 3’ v2 kit. A single-cell suspension was obtained by passing cells through a 35-μm cell strainer. Each group of cells was labeled with two Cell Multiplexing Oligo (CMO) tags from the CellPlex kit (10X Genomics). The tagged cells from different groups were pooled in equal proportions based on cell counts. Complementary DNA (cDNA) libraries were then constructed using the 10 × Genomics 3’ v2 kit. The libraries were sequenced on an Illumina NovaSeq platform (Novogene), and the resulting sequencing reads were aligned to the GRCm38/mm10 genome and demultiplexed using CellRanger (version 7.0.1). The generated count matrices were analyzed using Seurat (version 4.0.3) in R and Scanpy (version 1.8.2) in Python^59^.

### Cell plastic potential analysis

The cell plastic potential was computed following the protocol outlined by Qin et al.^39^. **Single-cell entropies:** Single-cell entropy was determined using the SCENT tool (v1.0.3). The scRNA-seq data, which had been normalized and logarithmized, were processed using Scanpy. Mouse gene symbols were mapped to human Entrez Gene identifiers utilizing the Orthology.eg.db (v3.18.0) and org.Mm.eg.db (v3.18.0) databases. The single-cell entropy was then calculated using the CCAT (Correlation of Connectome and Transcriptome) algorithm (CompCCAT(), ppiA=net17Jan16.m). **RNA velocity lengths:** RNA velocity lengths for single cells were extracted from scVelo’s dynamical modeling as previously described. **Single-cell PHATE coordinates:** The PHATE embedding for single cells was generated using the phateR package (v1.0.7)^38^. The normalized and log-transformed scRNA-seq data were provided as input, and the resulting PHATE coordinates were exported for downstream analyses. **Valley-Ridge (VR) scores:** The VR score was calculated as a weighted sum of two components: Valley and Ridge, with weights of 0.9 and 0.1, respectively. This computation was performed on a per sample and per cluster basis. The Valley component was defined as the median CCAT value for each sample-cluster combination. To compute the Ridge component, the inverse of the RNA velocity length was calculated and then scaled between 0 and 1. The cell centrality distance within each cluster was determined using the single-cell PHATE coordinates, with the Python function compute_distdeg() as defined by Qin et al.^39^. The knn parameter was optimized according to the size of each cluster. The Ridge component for each sample-cluster was then computed as the product of the median scaled inverse velocities and the scaled cell centrality distances. **Waddington-like landscapes:** The Waddington-like landscapes were visualized using Houdini Indie (SideFX, v20.0.533). In these visualizations, the VR scores were plotted along the y-axis, while the single-cell PHATE coordinates were positioned on the xz plane.

### GSVA and GSEA

The bulk RNA-seq data of TCGA (RRID:SCR_003193)-STAD patients were prepared by TCGAbiolinks. Briefly, the count matrix was downloaded by GDCquery(project = “TCGA-STAD”, data.category = “Transcriptome Profiling”, data.type = “Gene Expression Quantification”, workflow.type = “STAR - Counts”, experimental.strategy = ‘RNA-Seq’, sample.type=c(‘Primary Tumor’, ‘Solid Tissue Normal’). The clinical data were downloaded by GDCquery_clinic(project = “TCGA-STAD”, type = “Clinical”). Based on the sample barcodes (11 for ‘Solid Tissue Normal’, 01 for ‘Primary Solid Tumor’) and the ‘histological_type’ (only ‘Stomach, Adenocarcinoma, Diffuse Type’ was included) in the clinical data, the count matrix was sorted in the order of Normal and diffuse type STAD. The human gene Ensembl IDs inside of the count matrix were converted to HUGO gene symbols by biomaRt. GSEA was performed using the fgsea R package (v1.28.0). Genes were ranked according to the differential expression score statistics obtained from the DEG analysis. The ranked gene lists were evaluated against the Hallmark gene sets from the Molecular Signatures Database (MSigDB, v7.5.1).

### Cell-cell communication analysis

For ligand-receptor interaction-based cell-cell communication analysis of scRNA-seq datasets, the ‘CellChat’^60^ package in R was used. The integrated dataset was processed using the Seurat package, then the clustered and annotated datasets were analyzed by CellChat with default parameters (*p*-value threshold = 0.05). Epithelial cells were used as a source group, and immune cells were used as a target group.

### ATAC-seq

ATAC-seq libraries were prepared from 75,000 viable FACS-sorted cells using a modified Omni-ATAC protocol^61^. Briefly, nuclei were isolated in ATAC-resuspension buffer containing 0.1% NP40, 0.1% Tween-20, and 0.01% Digitonin. The isolated nuclei were subjected to transposition with Tn5 transposase (Illumina, #20034197) at 37 °C for 30 min. Transposed DNA was purified and pre-amplified with i7 index primers for 5 cycles. The optimal number of additional PCR cycles was determined by qPCR and applied to the pre-amplified products. Final libraries were purified, quantified, and sequenced on an Illumina platform. The libraries were sequenced using the Illumina NovaSeq platform at Novogene USA, with a read length of 150 paired-end base pairs and a sequencing depth of 50 million read pairs. The original sequencing data from the NovaSeq platform was transformed into raw reads by base calling. Raw reads were stored in fastq format files. Then, the fastq files were processed for the bioinformatic analyses.

### Motif-predicted binding sites

Bioinformatic analyses of ATAC-seq, including quality control of raw reads, adapter trimming, alignment with the reference genome, duplicate marking, reads filtering, peak calling, peak annotation, PCA, and replicates merging, were performed by nf-core/atacseq pipeline (v2.1.2)^62^. The insert size distribution plot, the heatmap of peaks binding to TSS regions, the Euclidean distance matrix, and the peak annotation bar plot were generated by this pipeline. The merged bigwig file per group containing tracks and peaks was loaded in the integrative genomics viewer for visualization.

### Statistical analyses

GraphPad Prism 9.4 (RRID:SCR_002798, Dogmatics) was used for statistical analyses. Student’s *t* test was used to compare two samples. *P-*values < 0.05 were considered statistically significant. Error bars indicate the standard deviation (s.d.), otherwise described in the Figure legends.

### Reporting summary

Further information on research design is available in the Nature Portfolio Reporting Summary linked to this article.

## Supplementary information

supplementary information

Description of Additional Supplementary Files

Supplementary dataset 1-4

Reporting Summary

Transparent Peer Review file

## Source data

Source data

## Data Availability

The single-cell RNA sequencing data generated in this study have been deposited in the Gene Expression Omnibus (GEO; RRID: SCR_007303) under accession code GSE284638. The ATAC-seq BigWig files generated in this study have been deposited in Zenodo under DOI 10.5281/zenodo. 18271217 (https://doi.org/10.5281/zenodo.18271217). All TCGA cancer patient sequencing data referenced in this study were obtained from the TCGA database through cBioPortal Cancer Genomics (http://www.cbioportal.org). Cancer cell line sequencing data from the Cancer Cell Line Encyclopedia (CCLE) were obtained through cBioPortal Cancer Genomics (http://www.cbioportal.org). Correlation analysis between *HIF1A* and *CD274* expression was performed using GEPIA2. Additional information and requests for resources and reagents should be directed to and will be fulfilled by the lead contact, Jae-Il Park (jaeil@mdanderson.org). All data relevant to the study are included in the article or described in the methods. Source data are provided in this paper.

## Code availability

The custom code used in this study has been archived in Zenodo (https://doi.org/10.5281/zenodo.21826128) and is also available on GitHub (https:// github.com/jaeilparklab/CRACD_DGAC).
