# Supplementary material for: CRACD loss drives hypoxia-induced immune escape in gastric cancer
Source: Nat Commun. 2026 Sep 1;17:10418. doi: 10.1038/s41467-026-77348-9 (PMC13631292; doi:10.1038/s41467-026-77348-9)
Supplement: Supplementary file 1 [file 41467_2026_77348_MOESM1_ESM.pdf]

## Supplementary Information

## List of Supplementary Materials

## Supplementary Figures 1-13

### Supplementary Figures

Supplementary Figure 1. Genetic alterations of *CRACD* in gastric cancer
Supplementary Figure 2. Analysis of *Cracd*-depleted gastric epithelia and organoids
Supplementary Figure 3. CRACD depletion induces cell plasticity of the gastric epithelia
Supplementary Figure 4. Single-cell transcriptomic analysis of GOs
Supplementary Figure 5. CRACD loss disrupts the actin cytoskeleton and promotes metastasis
Supplementary Figure 6. Immune profiling reveals reduced $CD8^+$ T cell infiltration in CRACD loss tumors
Supplementary Figure 7. Comparative analyses of gene regulatory networks
Supplementary Figure 8. Actin dysregulation by CRACD loss induces ROS accumulation
Supplementary Figure 9. Assessing the impact of CRACD point mutations on actin polymerization
Supplementary Figure 10. NF-κB–COX-2–NOX1 mediates CRACD loss-induces ROS accumulation
Supplementary Figure 11. Impact of HIF1α inhibition on CKP tumorigenesis and immune modulation
Supplementary Figure 12. Impact of anti-PD-L1 antibody on CKP tumorigenesis and immune modulation

## Supplementary References

## Supplementary Figures

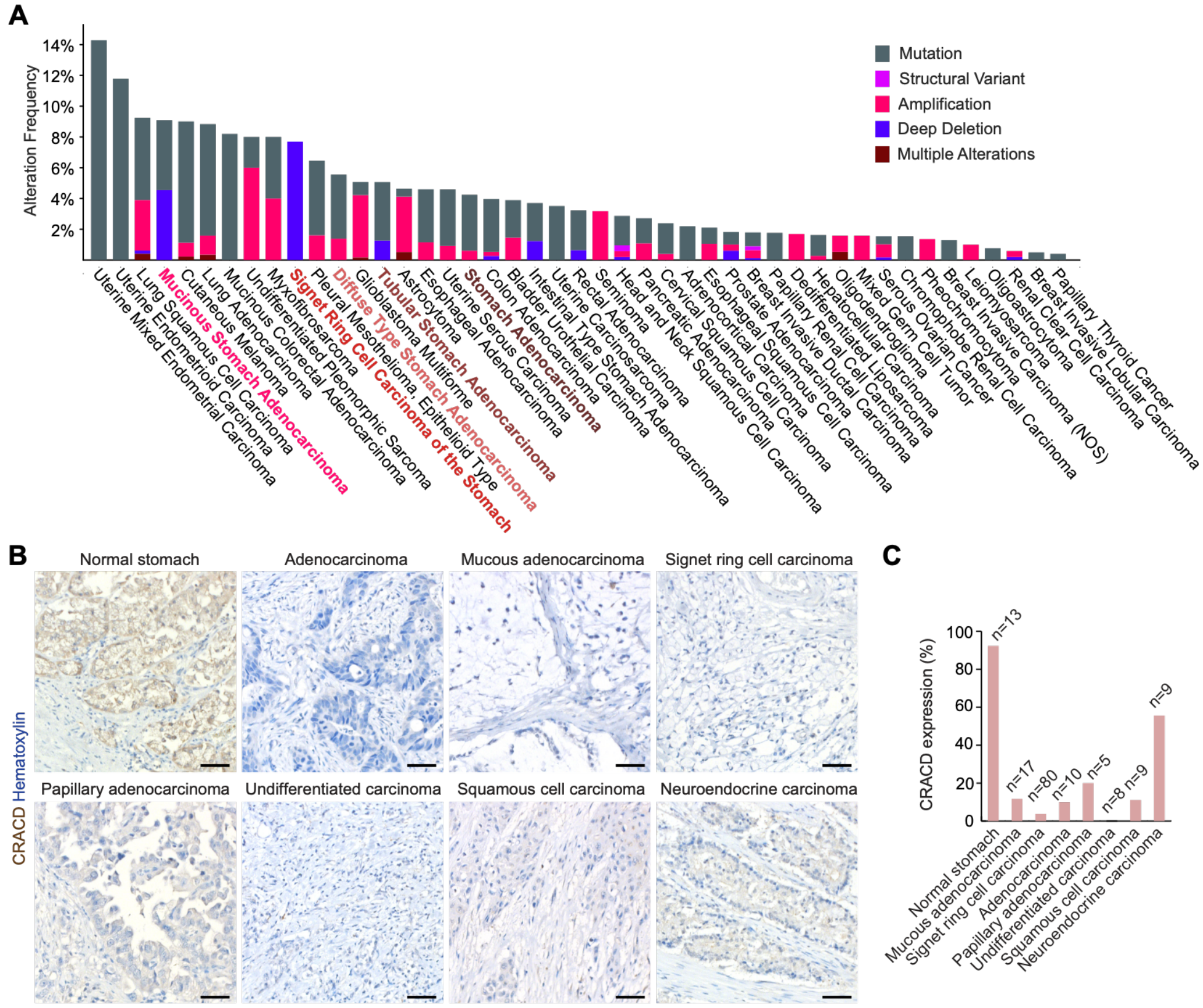


**Supplementary Figure 1. Genetic alterations of *CRACD* in gastric cancer**

**A.** cBioPortal analysis of TCGA Pan-Cancer datasets. 10,967 samples; the plot from cancer type detailed. **B, C.** Immunohistochemistry (IHC) of gastric cancer tissue microarray for CRACD protein; hematoxylin for nuclear counterstaining. Scale bars, 100 μm. Representative images are shown (B) with quantitative analysis (C).

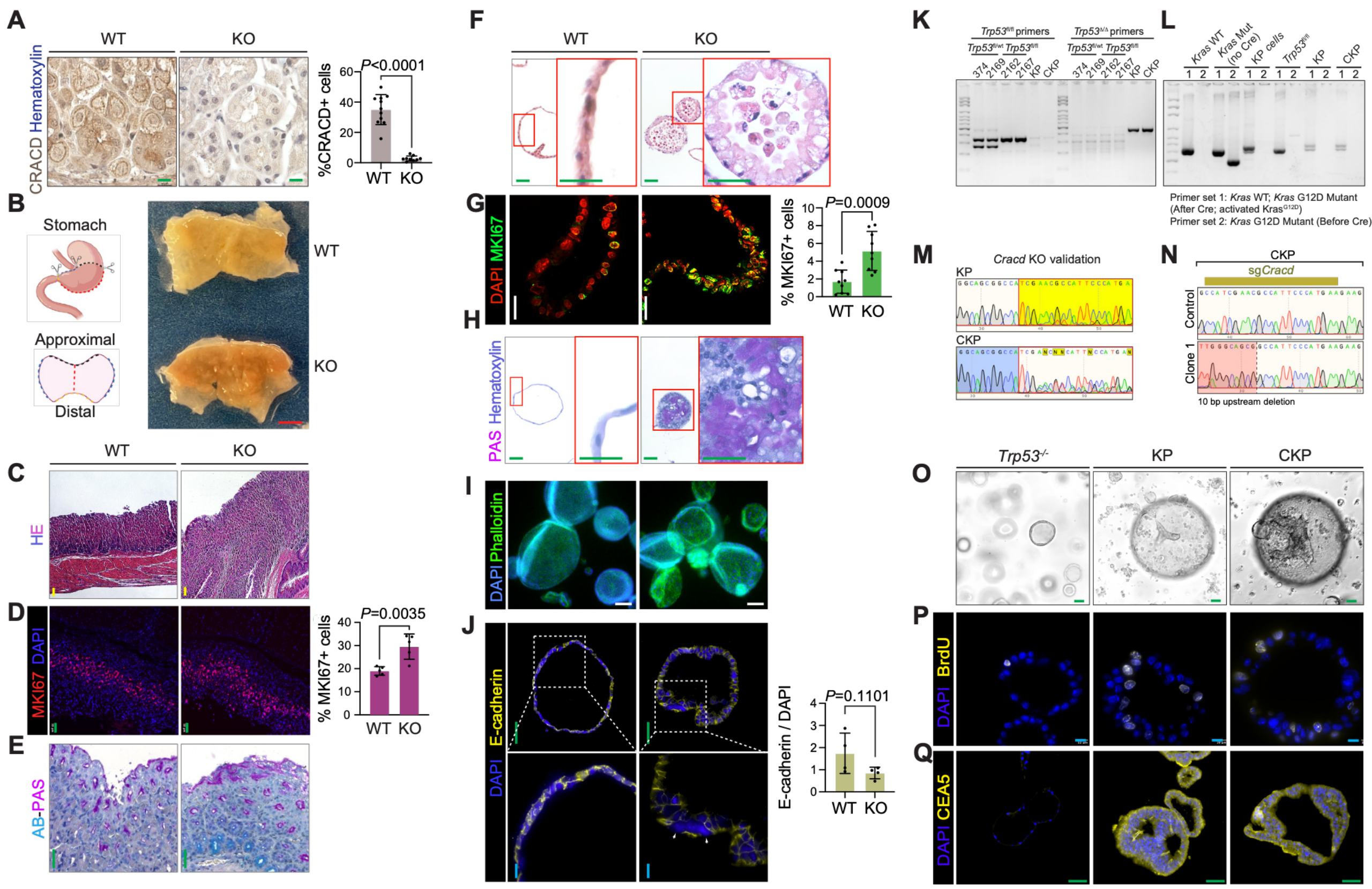


**Supplementary Figure 2. Analysis of Cracd-depleted gastric epithelia and organoids**

**A.** IHC of the mouse stomach tissues (*Cracd* WT or KO) for CRACD protein expression. **B.** Bright-field images of the stomach tissues isolated from *Cracd* WT or KO mice (7-month-old). Hyperplasia of the gastric epithelia was observed in *Cracd* KO mice. **C-E.** Histological analysis of the stomach tissues isolated from *Cracd* WT or KO mice. Hematoxylin and eosin (HE) (C), immunostaining for MKI67, a marker for proliferative cells (D), Alcian Blue-Periodic Acid-Schiff (AB-PAS), a marker for mucinous cells (E). Quantitation was performed using Student's *t*-test. **F-J.** Histological analysis of gastric organoids (GOs) derived from the gastric epithelial cells isolated from *Cracd* WT or KO mice. HE (F), MKI67 (G), Periodic Acid–Schiff (PAS) (H), and wholemount staining for phalloidin (I), E-cadherin (J). Quantitation was performed using Student's *t*-test and MKI67+ cells of WT, C, KP, and CKP GOs with one-way ANOVA. **K.** Genotyping of *Trp53*$^{fl/fl}$, KP, and CKP GOs for PCR with *Trp53*$^{fl/fl}$ and *Trp53*$^{del/del}$ primers. **L.** Genotyping of *Trp53*$^{fl/fl}$, KP, and CKP GOs with *Kras* WT and *Kras*$^{G12D}$ primers. **M.** Sanger sequencing results of KP and CKP GOs with *Cracd* primers. **N.** Sanger sequencing results of KP and CKP (clone 1) GOs with *Cracd* primers. **O.** Bright-field images of *Trp53*$^{fl/fl}$, KP, and CKP GOs. **P.** Bromodeoxyuridine (BrdU) incorporation assay of *Trp53*$^{fl/fl}$, KP, and CKP GOs. **Q.** Immunofluorescent (IF) staining of *Trp53*$^{fl/fl}$, KP, and CKP GOs for CEA5. Scale bars: green = 50 μm, blue = 10 μm; Student's *t*-test (unless specified); error bars: standard deviation; n>3; Representative images are shown; ns: not significant.

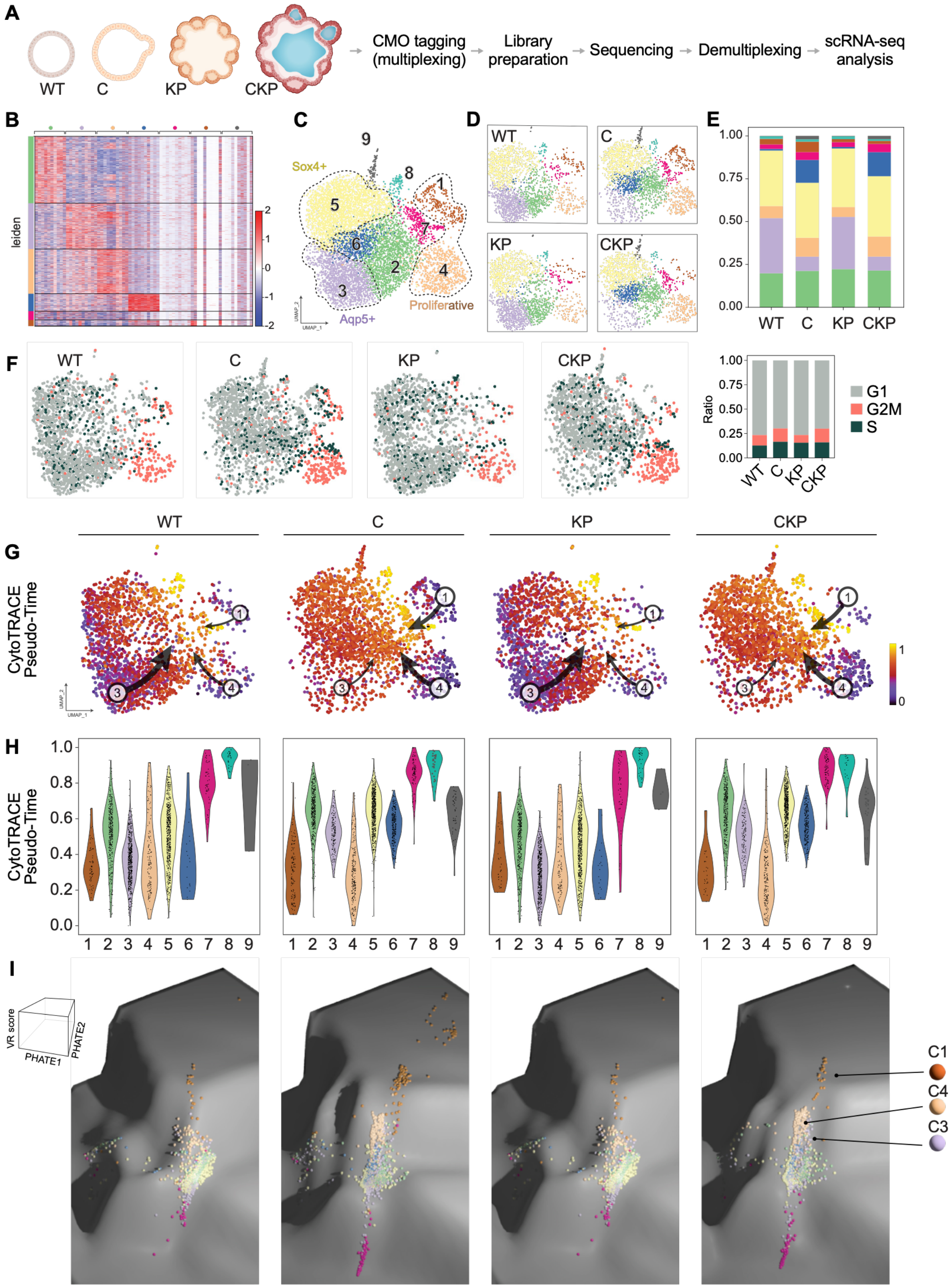


**Supplementary Figure 3. CRACD depletion induces cell plasticity of the gastric epithelia.**

**A.** Workflow for single-cell RNA sequencing (scRNA-seq). **B.** Heatmap displaying gene expression of markers distinguishing each cell cluster. **C.** Uniform manifold approximation and projection (UMAP) plot of cell clustering for whole cells from wildtype (WT), *Cracd* WT, *Cracd*$^{-/-}$ (C), *Kras*$^{G12D}$ *Trp53*$^{-/-}$ (KP), and *Cracd*$^{-/-}$ *Kras*$^{G12D}$ *Trp53*$^{-/-}$ (CKP) gastric organoids (GOs). **D.** UMAP plots showing cell clustering for WT, C, KP, and CKP GOs separately. **E.** Bar plot showing the enrichment patterns of clusters in each WT, C, KP, and CKP GO. **F.** UMAP plots depicting the cell cycle states of individual cells from WT, C, KP, and CKP GOs. **G.** CytoTRACE pseudotime inference analysis of WT, C, KP, and CKP GOs. Lower values indicate less differentiated states. **H.** Violin plots illustrating the distribution of CytoTRACE pseudotime values across each cluster for WT, C, KP, and CKP GOs. **I.** Waddington-like embedding of epithelial cells from WT, C, KP, and CKP GOs. The landscape was derived from PHATE coordinates and VR scores.

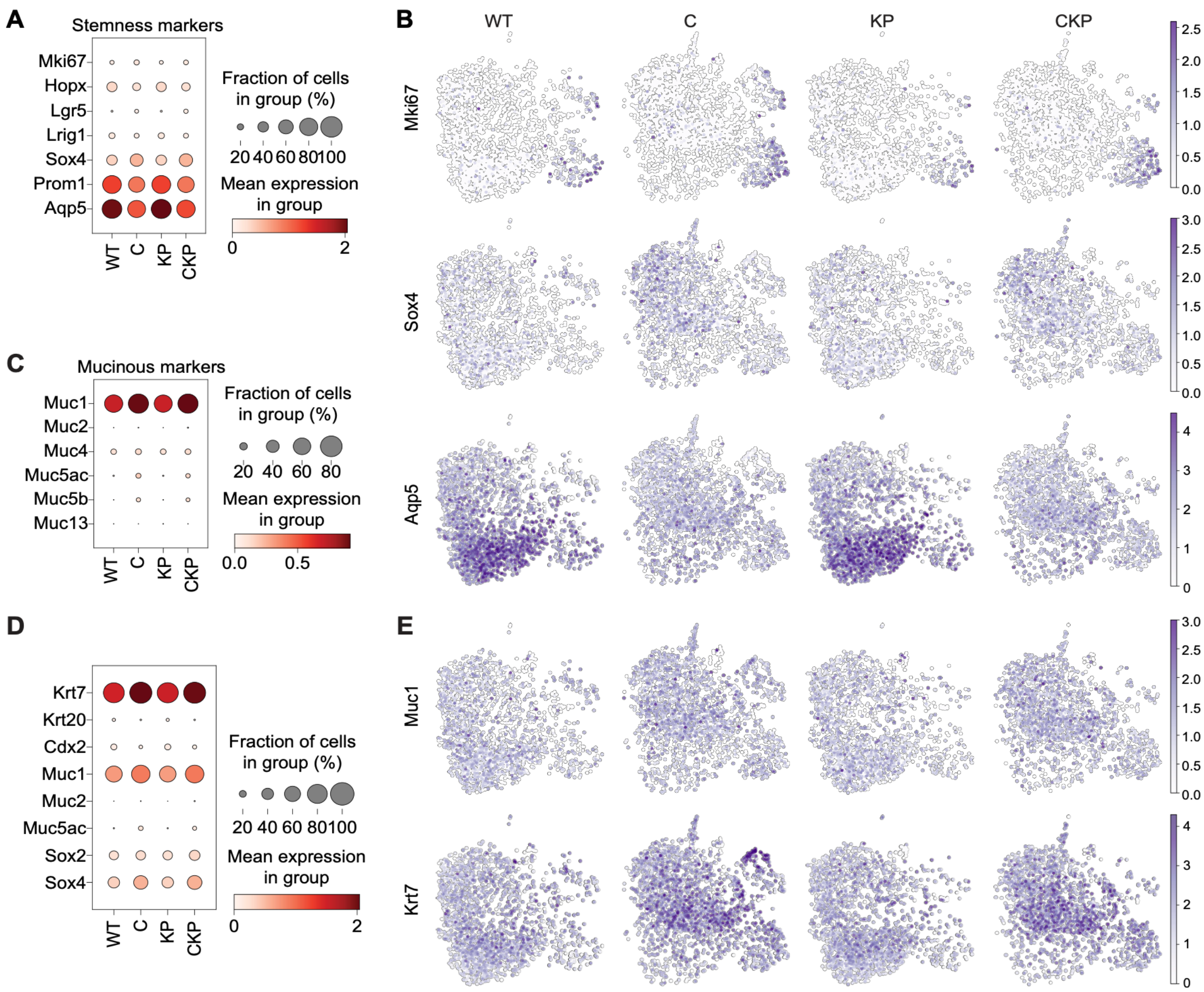


**Supplementary Figure 4. Single-cell transcriptomic analysis of GOs**

**A.** Dot plot showing the expression of genes related to gastric epithelium stemness in each type of GOs (WT, C, KP, and CKP). **B.** Feature plots of *Mki67*, *Sox4*, and *Aqp5* of WT, C, KP, and CKP GOs. **C, D.** Dot plot showing the expression of mucinous (C) and clinical (D) markers in each type of GOs. **E.** Feature plots displaying the expression of *Muc1* and *Krt7* of WT, C, KP, and CKP GOs.

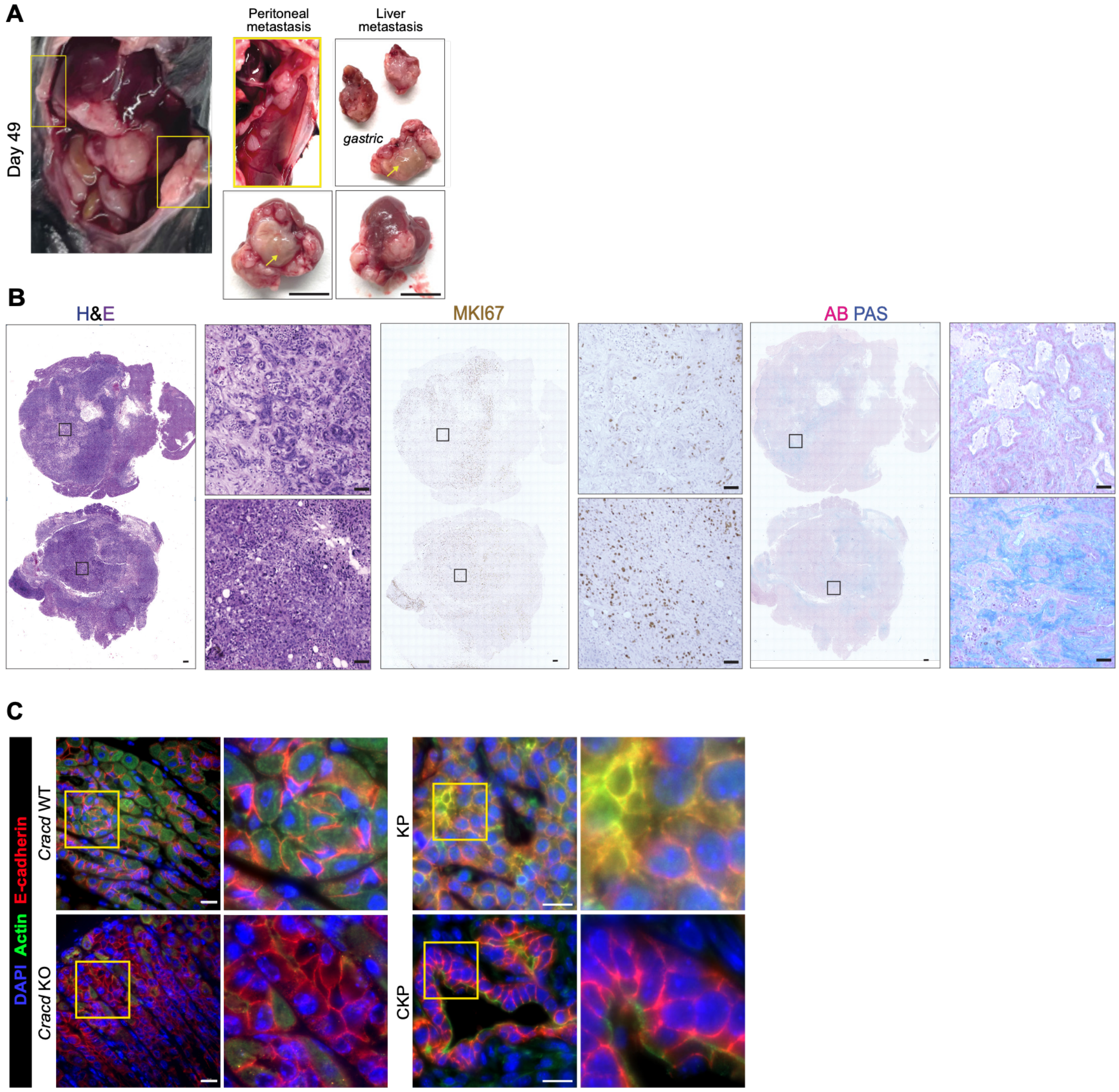


**Supplementary Figure 5. CRACD loss disrupts the actin cytoskeleton and promotes metastasis**
**A.** Representative gross images showing peritoneal, liver metastasis, and gastric metastatic nodules (yellow arrows). Scale bars: 1 cm (gross images). **B.** H&E, MKI67, and AB-PAS staining of metastatic lesions, revealing poorly differentiated histology. 500 μm (low magnification), 50 μm (high magnification). **C**. Immunofluorescence staining of actin (green), E-cadherin (red), and DAPI (blue) in (left) gastric tissues from *Cracd* WT and KO mice, and (right) orthotopic KP and CKP tumors. Scale bars: 20 μm.

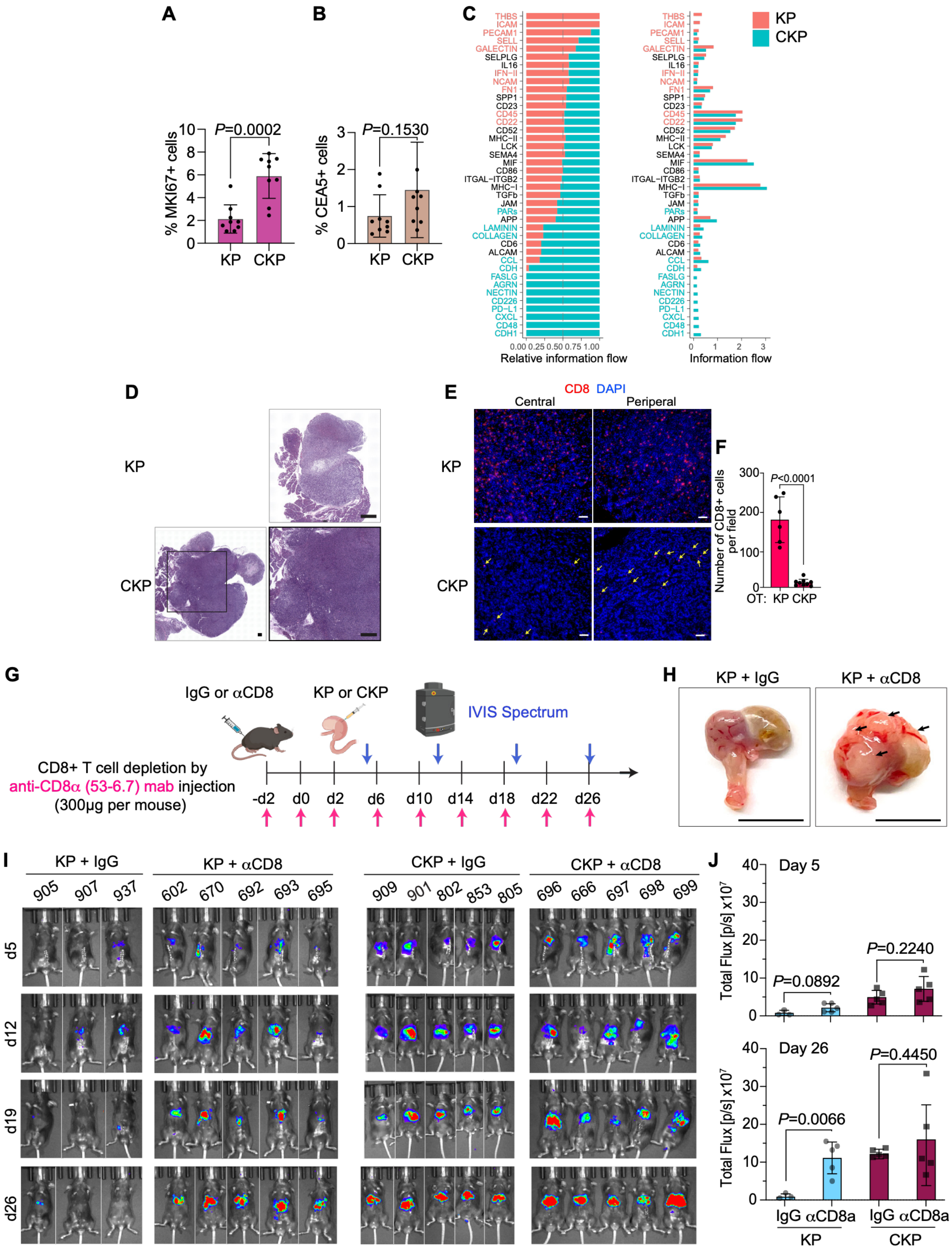

**Supplementary Figure 6. Immune profiling reveals reduced CD8$^{+}$ T cell infiltration in CRACD loss tumors**
**A, B.** Statistical analysis of MKI67- (A) and CEA5- (B) positive cells in allograft tumors (KP vs. CKP). **C.** Cell-cell interaction analysis of allograft tumors (KP vs. CKP). Relative and absolute information flow of ligand-receptor interactions in KP and CKP tumor cells; analyzed using the CellChat package of scRNA-seq datasets of whole tumors (KP vs. CKP). **D**. Representative H&E images of KP and CKP orthotopic tumors (scale bars: low magnification, 500 μm; high magnification, 100 μm). **E**. Immunofluorescence staining for CD8 (red) and DAPI (blue) in KP and CKP tumors. Yellow arrows indicate CD8$^{+}$ T cells localized in CKP tumors. **F**. Quantification of CD8$^{+}$ T cell density in KP and CKP orthotopic tumors. Data are presented as mean ± SEM, and statistical

significance was determined by Student's *t*-test. **G**. Schematic diagram of the experimental design. C57BL/6 mice orthotopically transplanted with KP and CKP cells were treated intraperitoneally with anti-CD8α (clone 53-6.7) antibody or control IgG at the indicated time points. The schematic was created with BioRender.com and subsequently edited. **H**. Representative gross images of orthotopic tumors from KP control IgG and anti-CD8α groups (Scale bars: 1cm). **I.** Bioluminescence images showing tumor growth dynamics in each treatment group over the experimental period. **J**. Quantification of photon flux. Data are presented as mean ± SEM, and statistical significance was determined by Student's *t*-test.

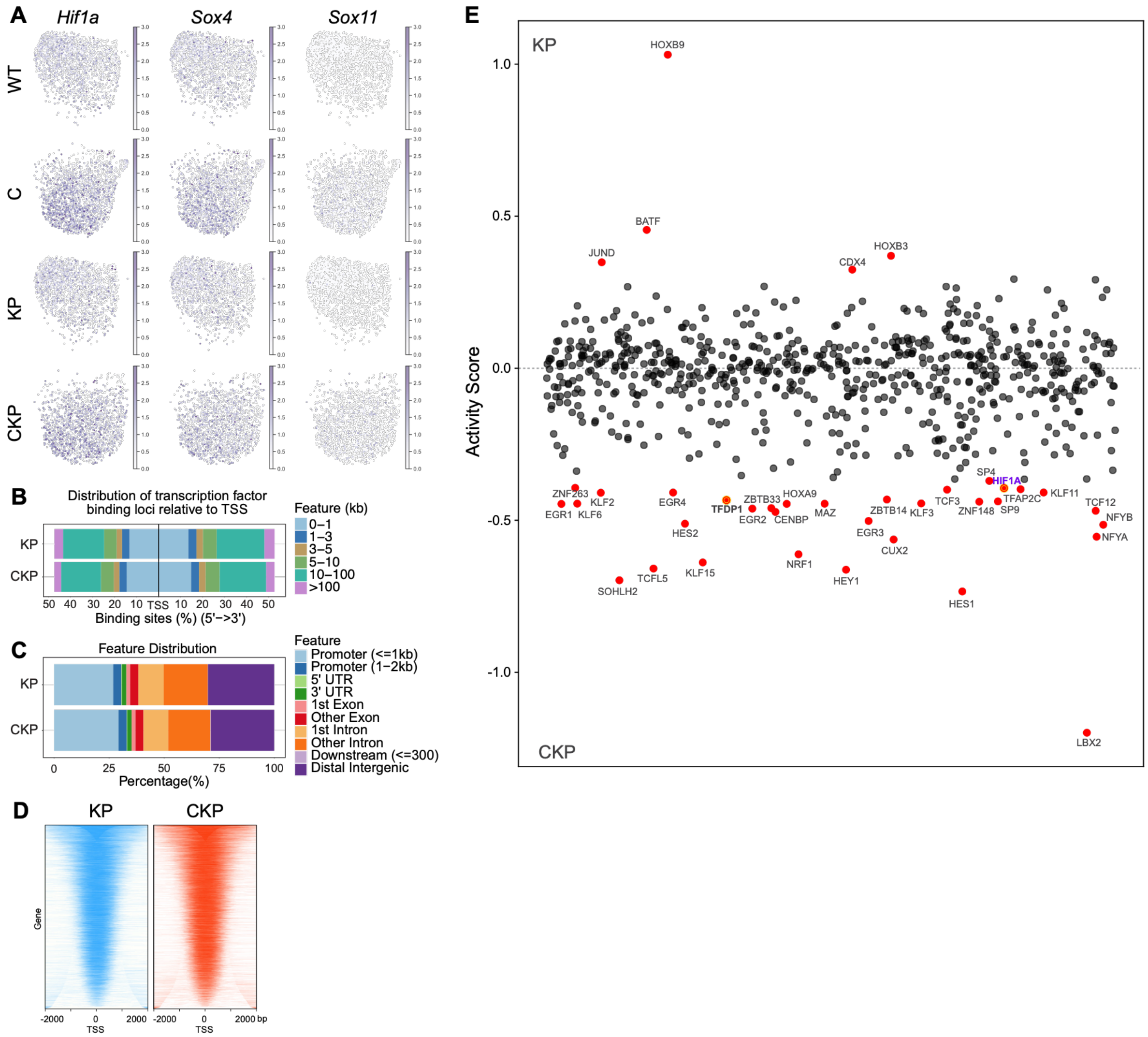


**Supplementary Figure 7. Comparative analyses of gene regulatory networks**

**A.** Feature plots visualizing the expression of transcription factor [1], *Hif1a*, *Sox4*, and *Sox11* on the UMAPs of each GOs (WT, C, KP, and CKP). **B-D.** ATAC-seq analysis of KP and CKP tumor cells. Stacked bar plots displaying the distribution of TFs' binding loci relative to transcription start sites (TSS) (B) and features (C). Heatmap of ATAC-seq peaks around transcription start site regions identifying differences in gene regulation between KP and CKP (D). **E.** Activity scores of transcription factors in KP and CKP tumors, showing differential regulatory networks. Highlighted transcription factors, including *Hif1a*, *Sox4*, and *Sox11*, exhibit higher activity in CKP cells. Red dots represent transcription factors with significant alterations between the two groups (KP and CKP).

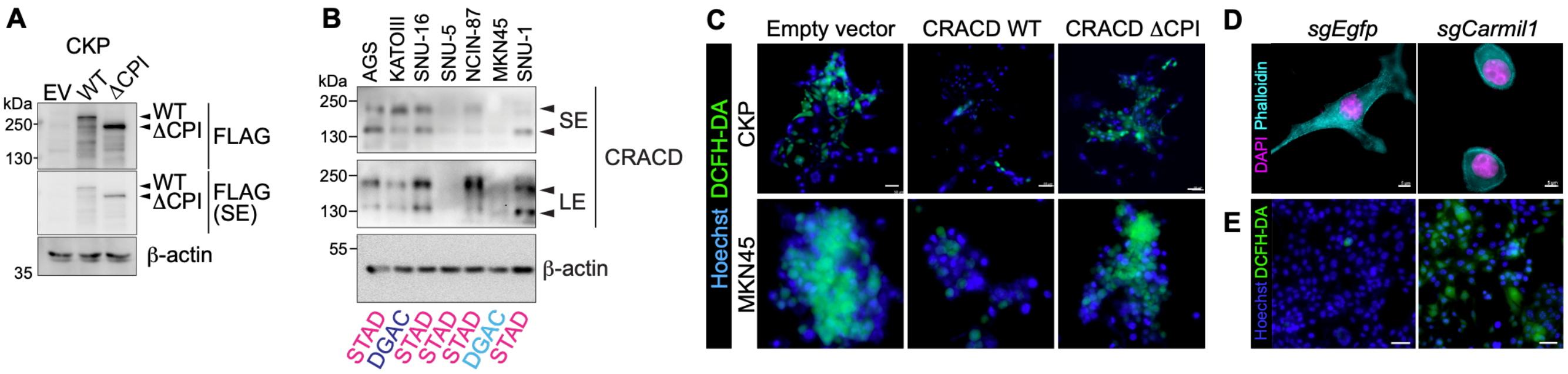


**Supplementary Figure 8. Actin dysregulation by CRACD loss induces ROS accumulation**

**A.** Immunoblot analysis confirming expression of CRACD WT and ΔCPI mutant constructs in CKP cells. **B.** Immunoblots of human gastric cancer cell lines. STAD: stomach adenocarcinoma, MKN45 cells carry nonsense mutations (Q186Tfs), the most frequent frame-shift mutations in *CRACD*. **C.** Representative fluorescence images of CKP cells transduced with empty vector, CRACD WT, or ΔCPI mutant, stained with DCFH-DA (green, ROS) and Hoechst (blue, nuclei). Scale bars: 50 μm. **D, E.** Representative immunofluorescence staining of F-actin (phalloidin) in *Carmil1* KO cells, showing loss of organized actin filaments. Scale bars: 5 μm (upper), 50 μm (lower).

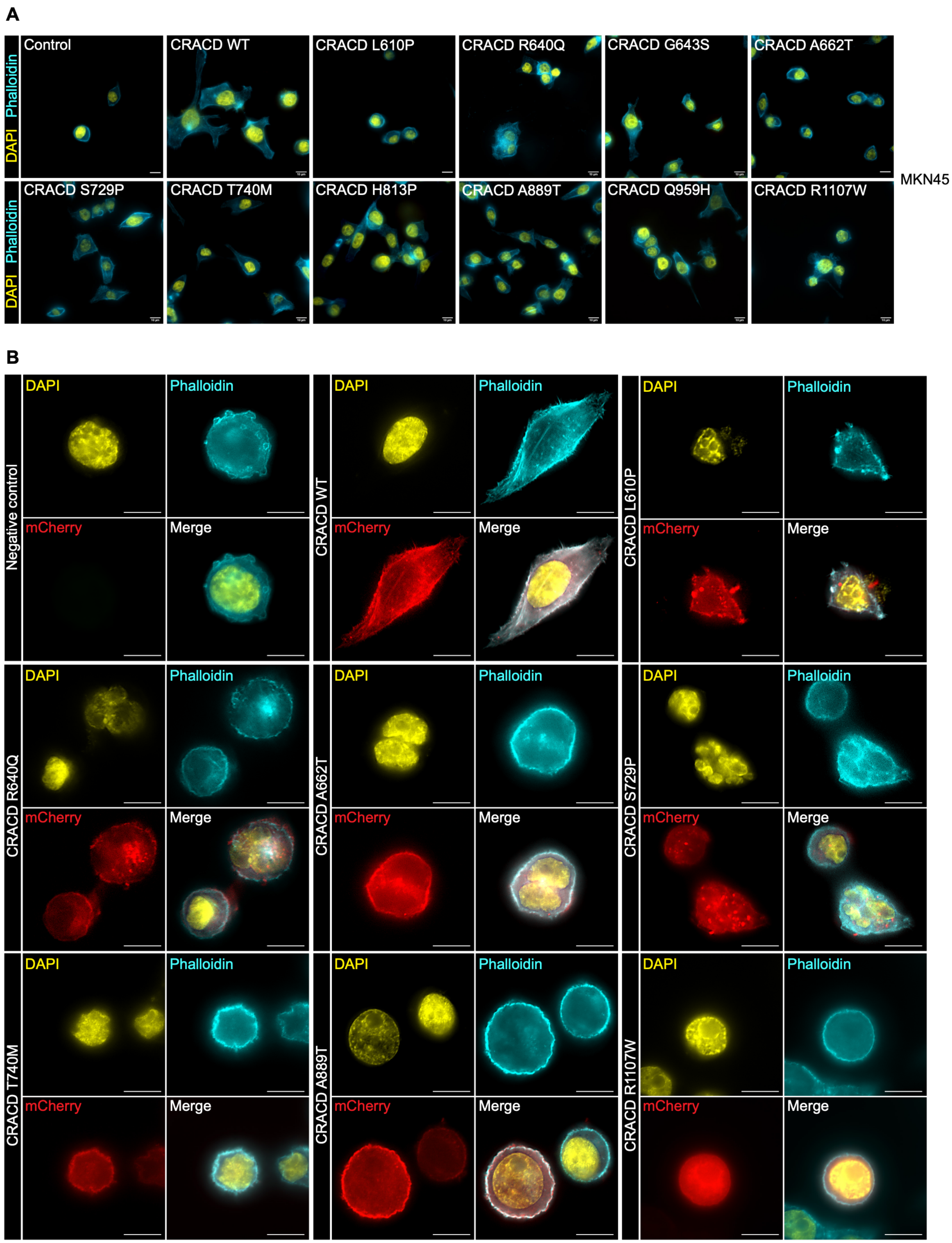


**Supplementary Figure 9. Assessing the impact of CRACD point mutations on actin polymerization**
**A, B.** MKN45 cells carrying a nonsense mutation in the CRACD alleles were transduced with lentiviruses encoding mCherry-tagged CRACD wild type (WT) or other point mutations. F-actin was visualized by phalloidin staining. Scale bars: 10 μm (A); 100 μm (B).

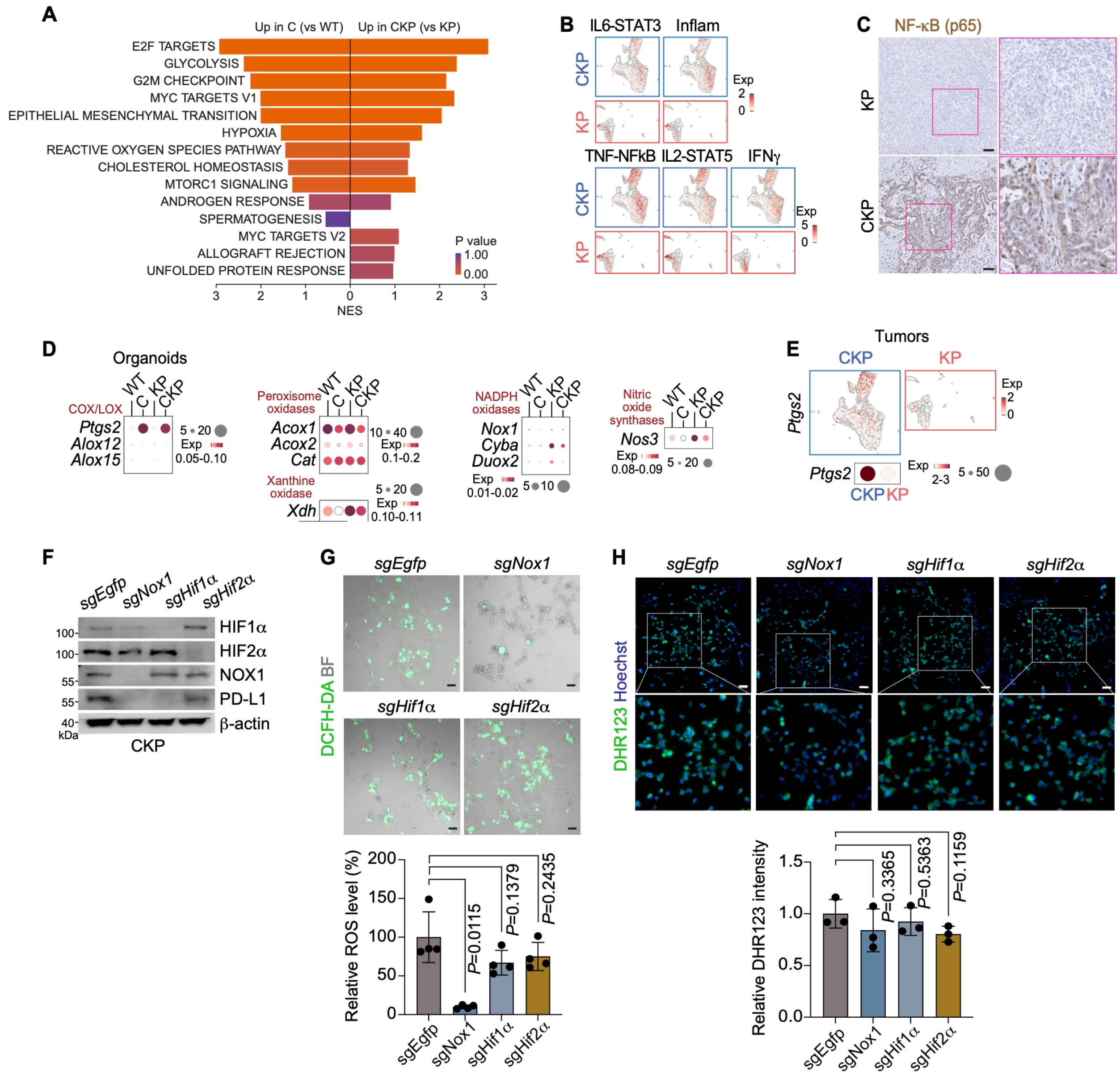

**Supplementary Figure 10. NF-κB–COX-2–NOX1 mediates CRACD loss-induces ROS accumulation**

**A**. Hallmark pathways that were upregulated in C organoids compared to WT (left) and in CKP organoids compared to KP (right) are shown. Each bar represents the normalized enrichment score (NES) of a pathway, and the color of the bar corresponds to its p-value. **B.** Hallmark pathway activity in tumor cells from orthotopic tumors. UMAP plots showing enrichment scores for IL6-STAT3, inflammatory response, TNF-NFκB, IL2-STAT5, and IFNγ pathways in CKP (top) and KP (bottom). **C**. Immunohistochemistry for NF-kB (p65) in orthotopic KP and CKP tumors; scale bars, 50 μm. **D.** Expression of oxidative enzyme genes in tumor organoids. Dot plots showing the relative expression of COX/LOX (*Ptgs2*, *Alox12*, and *Alox15*), peroxisome oxidases (*Acox1*, *Acox2*, and *Cat*), xanthine oxidase (*Xdh*), NADPH oxidases (*Nox1*, *Cyba*, and *Duox2*), and nitric oxide synthases (*Nos3*) across WT, C, KP, and CKP organoids. **E.** Expression of *Ptgs2* in tumor cells from orthotopic tumors. UMAP feature plots show *Ptgs2* expression in CKP and KP tumors (top), with corresponding dot plot summarizing expression level and fraction of expressing cells (bottom). **F.** Immunoblot analysis of HIF1α, HIF2α, NOX1, PD-L1 and β-actin in CKP cells. **G.** Representative fluorescence images of DCFH-DA staining (green) in CKP cells. Quantification of DCFH-DA/ROS levels is shown below. Fluorescence intensity was normalized to the sg*Egfp* control group. **H.** Representative images of DHR123 staining (green) and Hoechst nuclear staining (blue) in CKP cells. Quantification of DHR123 fluorescence intensity normalized to Hoechst-positive cells is shown below.

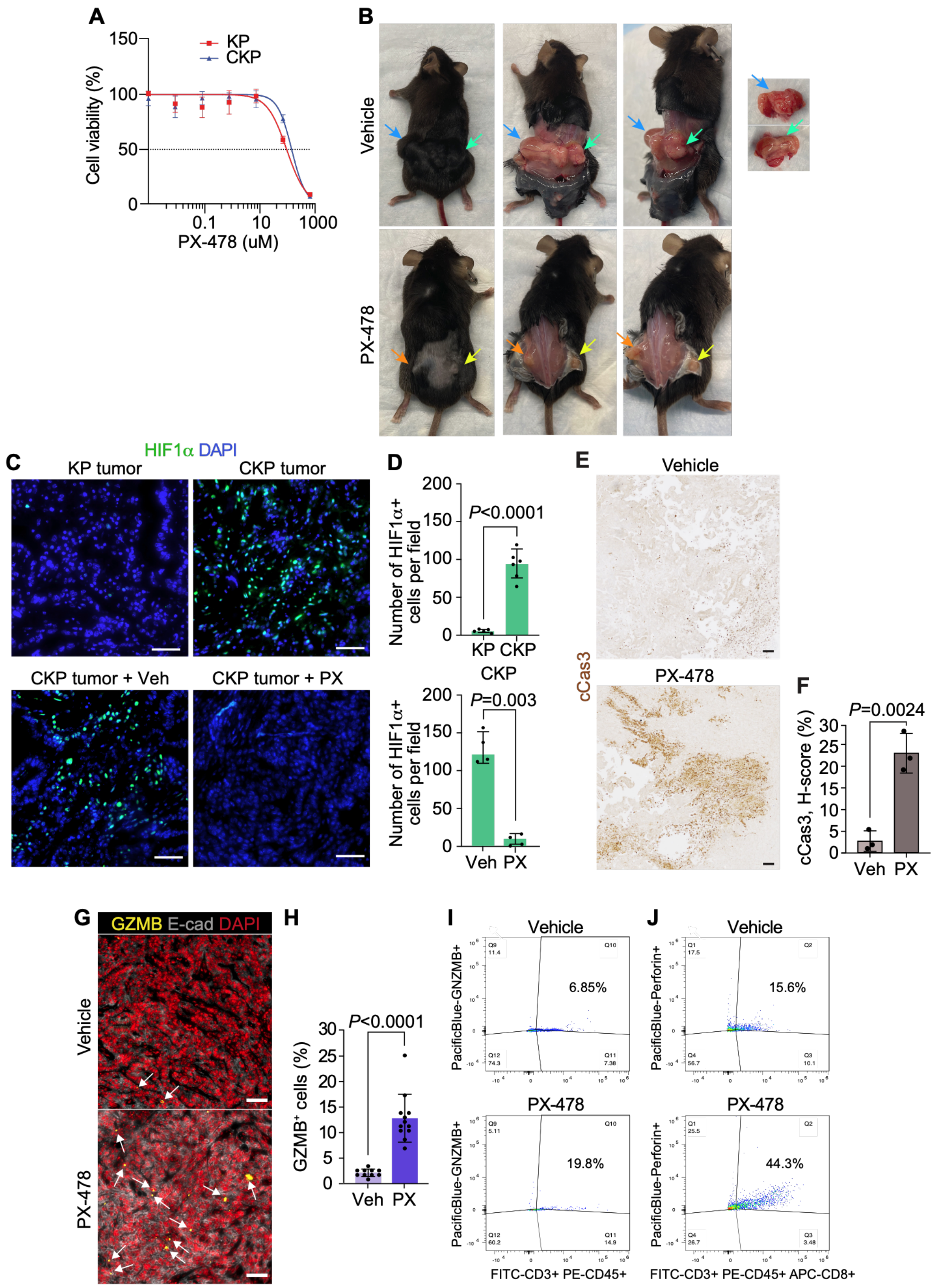


**Supplementary Figure 11. Impact of HIF1α inhibition on CKP tumorigenesis and immune modulation**
**A.** Cell viability assay of KP and CKP 2D cell lines treated with a *HIF1α* inhibitor (PX-478). **B.** Photos of immunocompetent mice (C57BL/6; vehicle vs. PX-478) bearing CKP allograft tumors. Tumors display invasive growth, penetrating the muscularis propria, and diffuse tumor formation. **C, D.** Representative immunofluorescence HIF1α (green) staining (C). Scale bars: 50 µm. Quantification of HIF1α$^+$ cells (D). Data are presented as mean ± SEM, and statistical significance was determined by Student's *t*-test. **E, F.** Immunohistochemical images (E) and quantification (F) of CKP tumors for cleaved Caspase-3 (cCas3) and MKI67. Scale bars, 100 µm. **G, H.** IF staining images (G) and quantification (H) of CKP allograft tumors post-*HIF1α* inhibitor treatment for Granzyme B (GZMB) and E-cadherin. Scale bars: 50 µm; arrows: Granzyme B and E-cadherin double-positive cells. **I, J.** Flow cytometric analysis of whole cells in CKP allograft tumors from mice treated with vehicle (control) or PX-478 for Granzyme B-positive (I) or Perforin-positive (J) T cells, a *HIF1α* inhibitor. Student's *t*-test; error bars: standard deviation; n>3; Representative images and plots are shown.

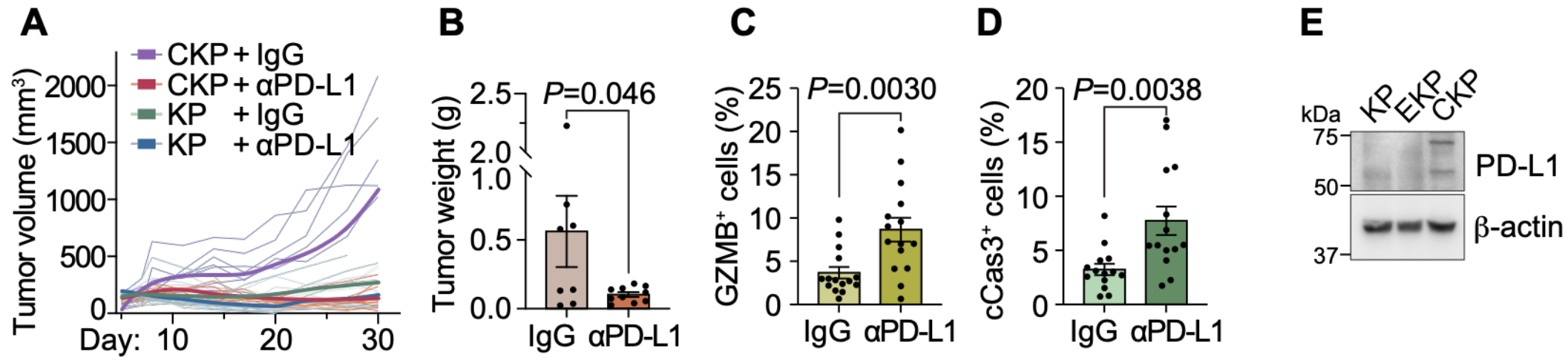


**Supplementary Figure 12. Impact of anti-PD-L1 antibody on CKP tumorigenesis and immune modulation**
**A, B.** Tumor growth curves (A) and weight assessment (B) of subcutaneously transplanted *Kras$^{G12D}$ Trp53$^{-/-}$* (KP) and CKP cells treated with IgG or anti-PD-L1 antibody (10 mg/kg, by intraperitoneal injection). Darker lines, median values of each group. Representative images are shown (n > 3); Student's t-test; error bars, SD. **C, D.** Quantification of GZMB- (C) and cleaved caspase-3 (cCas3)- (D) positive signals from CKP tumor immunofluorescence staining shown in the Figure. 6G. Representative images are shown (n>3); Student's t-test; error bars, SD. **E.** Immunoblot analysis of PD-L1 expression.

## References


1 Hammerman, P. S. *et al.* Comprehensive genomic characterization of squamous cell lung cancers. *Nature* **489**, 519-525 (2012). https://doi.org/10.1038/nature11404
