# Supplementary material for: CRACD loss drives hypoxia-induced immune escape in gastric cancer
Source: Nat Commun. 2026 Sep 1;17:10418. doi: 10.1038/s41467-026-77348-9 (PMC13631292; doi:10.1038/s41467-026-77348-9)
Supplement: Supplementary file 2 [file 41467_2026_77348_MOESM2_ESM.pdf]

Description of Additional Supplementary Files

Supplementary Dataset 1: Reagent information

Supplementary Dataset 2: Tissue microarray information (ST821)

Supplementary Dataset 3: In-house tissue microarray information

Supplementary Dataset 4: Clinical annotation for patient-derived organoids
