# Supplementary material for: CRACD loss drives hypoxia-induced immune escape in gastric cancer
Source: Nat Commun. 2026 Sep 1;17:10418. doi: 10.1038/s41467-026-77348-9 (PMC13631292; doi:10.1038/s41467-026-77348-9)
Supplement: Supplementary file 3 [file 41467_2026_77348_MOESM4_ESM.pdf]

nature portfolio

Corresponding author(s): Hyunki Kim, Jae-Il Park

Last updated by author(s): 08/06/2026

Reporting Summary

Nature Portfolio wishes to improve the reproducibility of the work that we publish. This form provides structure for consistency and transparency in reporting. For further information on Nature Portfolio policies, see our Editorial Policies and the Editorial Policy Checklist.

Statistics

For all statistical analyses, confirm that the following items are present in the figure legend, table legend, main text, or Methods section.

n/a	Confirmed	
☐	☒	The exact sample size (*n*) for each experimental group/condition, given as a discrete number and unit of measurement
☐	☒	A statement on whether measurements were taken from distinct samples or whether the same sample was measured repeatedly
☐	☒	The statistical test(s) used AND whether they are one- or two-sided<br>*Only common tests should be described solely by name; describe more complex techniques in the Methods section.*
☒	☐	A description of all covariates tested
☐	☒	A description of any assumptions or corrections, such as tests of normality and adjustment for multiple comparisons
☐	☒	A full description of the statistical parameters including central tendency (e.g. means) or other basic estimates (e.g. regression coefficient) AND variation (e.g. standard deviation) or associated estimates of uncertainty (e.g. confidence intervals)
☐	☒	For null hypothesis testing, the test statistic (e.g. *F*, *t*, *r*) with confidence intervals, effect sizes, degrees of freedom and *P* value noted<br>*Give P values as exact values whenever suitable.*
☒	☐	For Bayesian analysis, information on the choice of priors and Markov chain Monte Carlo settings
☒	☐	For hierarchical and complex designs, identification of the appropriate level for tests and full reporting of outcomes
☐	☒	Estimates of effect sizes (e.g. Cohen's *d*, Pearson's *r*), indicating how they were calculated

Our web collection on statistics for biologists contains articles on many of the points above.

Software and code

Policy information about availability of computer code

Data collection	Flow cytometry data were collected using Life Technologies Attune software. Images were captured using Axio Observer Z1M (Zeiss), Nikon confocal microscope, ECHO Revolve (ECHO), and Olympus IX73 (Olympus) microscopes. Image adjustment was performed using ZEN software (v3.0, blue edition, Zeiss). Sequencing data were generated using Illumina NovaSeq 6000 software (v1.5).
Data analysis	Bulk RNA-seq data were analyzed using STAR (v2.7.10a), featureCounts (v2.0.6), and DESeq2 (v1.42.0). Single-cell RNA-seq data were processed and analyzed using Cell Ranger (v7.0.1, 10x Genomics), Seurat (v4.4.0), Scanpy (v1.9.3), scVelo (v0.3.2), and Dynamo (v1.3.0). Cell–cell communication was analyzed using CellChat (v1.6.1). Gene set enrichment analyses were performed with fgsea (v1.28.0). Additional analyses were performed with pandas (v2.0.3), infercnvpy (v0.1.0), and PHATE (v1.0.11). Statistical analyses were performed using GraphPad Prism (v9.4.0, Dotmatics). No custom code was developed beyond the tools cited above.<br><br>The custom code generated in this study has been deposited in Zenodo under DOI 10.5281/zenodo.21826128. The code is also available on GitHub at github.com/jaeilparklab/CRACD_DGAC.

For manuscripts utilizing custom algorithms or software that are central to the research but not yet described in published literature, software must be made available to editors and reviewers. We strongly encourage code deposition in a community repository (e.g. GitHub). See the Nature Portfolio guidelines for submitting code & software for further information.

Data

Policy information about availability of data

All manuscripts must include a data availability statement. This statement should provide the following information, where applicable:

- Accession codes, unique identifiers, or web links for publicly available datasets
- A description of any restrictions on data availability
- For clinical datasets or third party data, please ensure that the statement adheres to our policy

The single-cell RNA sequencing data generated in this study have been deposited in the Gene Expression Omnibus (GEO; RRID: SCR_007303) under accession code GSE284638. The reviewer access token is qdermiqsflmnlep.

Research involving human participants, their data, or biological material

Policy information about studies with human participants or human data. See also policy information about sex, gender (identity/presentation), and sexual orientation and race, ethnicity and racism.

Reporting on sex and gender	Sex was recorded as a biological variable based on clinical records. No sex-based stratified analyses were performed, as the study was not designed or powered to detect sex-specific differences in gene expression.
Reporting on race, ethnicity, or other socially relevant groupings	Race and ethnicity were not analyzed in this study, as the cohort consisted of patients treated at a single institution and race/ethnicity was not considered relevant to the biological questions addressed.
Population characteristics	The study included resected tumor specimens from patients with advanced gastric cancer (pathological stage ≥T2).<br>A total of 200 consecutive surgical cases from 2024 were screened, and tissue microarrays (TMAs) were constructed using 3-mm cores (two cores per case).<br>Immunohistochemical analyses were performed on 184 evaluable tumor samples.<br>Available clinicopathological variables included TNM stage, WHO classification, Lauren classification, EBV status, MSI status, PD-L1 expression (22C3; TPS and CPS), and HER2 status.<br>Approximately 40–50% of tumors were classified as diffuse-type or intestinal-type gastric cancer, enabling comparative analyses between histological subtypes.<br>Patient-derived organoids (PDOs) were established from a subset of diffuse-type (n=3) and intestinal-type (n=2) gastric cancer samples and used for exploratory protein expression analyses by immunoblotting.
Recruitment	Tumor specimens were retrospectively identified from consecutive surgical cases at a single institution. No active recruitment or patient contact was involved.
Ethics oversight	The study was approved by the Institutional Review Board (IRB) of the participating institution.<br>All human tissue samples were obtained from surgical specimens, and the requirement for informed consent was waived due to the retrospective nature of the study.

Note that full information on the approval of the study protocol must also be provided in the manuscript.

Field-specific reporting

Please select the one below that is the best fit for your research. If you are not sure, read the appropriate sections before making your selection.

☒ Life sciences ☐ Behavioural & social sciences ☐ Ecological, evolutionary & environmental sciences

For a reference copy of the document with all sections, see nature.com/documents/nr-reporting-summary-flat.pdf

Life sciences study design

All studies must disclose on these points even when the disclosure is negative.

Sample size	For all in vitro experiment, the number of samples sizes are three or above. For animal studies, sample sizes were estimated by power calculation.
Data exclusions	No data were excluded from the analyses. All collected data that passed quality control were included in the study.
Replication	All experiments presented in this study were performed using at least three biological replicates, unless otherwise stated. All presented findings were reproducible across biological replicates.
Randomization	Mice were randomly assigned to experimental groups to minimize selection bias. For in vitro assays, randomization was not relevant to the study design.
Blinding	Investigators were not blinded during data collection or analysis. Given the objective and quantitative nature of the data, blinding was not deemed necessary.

Reporting for specific materials, systems and methods

We require information from authors about some types of materials, experimental systems and methods used in many studies. Here, indicate whether each material, system or method listed is relevant to your study. If you are not sure if a list item applies to your research, read the appropriate section before selecting a response.

Materials & experimental systems

n/a	Involved in the study	
☐	☒	Antibodies
☐	☒	Eukaryotic cell lines
☒	☐	Palaeontology and archaeology
☐	☒	Animals and other organisms
☐	☒	Clinical data
☒	☐	Dual use research of concern
☒	☐	Plants

Methods

n/a	Involved in the study	
☒	☐	ChIP-seq
☐	☒	Flow cytometry
☒	☐	MRI-based neuroimaging

Antibodies

Antibodies used

Antibodies Source Cat # Dilution Application
KIAA1211/CRACD Atlas Antibodies HPA043249 1:50 IHC
CRACD Cell Signaling Technology 39862 1:2000 WB
MKi67 abcam ab16667 1:100 IHC, IF
Phalloidin Thermo O7466 1:100 IHC, IF
Phalloidin Cell Signaling Technology 13054 1:100 IHC, IF
MUC1 Proteintech 23614-1-AP 1:200 IHC, IF
MUC2 Proteintech 27675-1-AP 1:200 IHC, IF
MUC4 ThermoFisher 35-4900 1:200 IHC, IF
EpCAM Cell Signaling Technology 93790 1:200 IHC, IF
E-cadherin BD 610182 1:100 IHC, IF
E-cadherin Cell Signaling Technology 3195 1:100 IHC, IF
BrdU abcam ab6326 1:200 IHC, IF
CEACAM5 Invitrogen PA5-88533 1:200 IHC, IF
Beta-catenin Cell Signaling Technology 8480 1:100 IHC, IF
Cleaved caspase-3 Cell Signaling Technology 9664 1:100 IHC, IF
HIF1A Cell Signaling Technology 14179 1:100 IHC, IF
HIF1A Cell Signaling Technology 36169 1:1000 WB
CD8 Cell Signaling Technology 98941 1:100 IHC, IF
NF-kB p65 Cell Signaling Technology 8242 1:100 IHC, IF
Actin abcam ab50591 1:100 IHC, IF
PDL1 Agilent 22C3 1:1000 WB
PDL1 Cell Signaling Technology 60475 1:1000 WB
HIF2A Proteintech 26422-1-AP 1:1000 WB
NOX1 abcam ab131088 1:1000 WB
b-actin Proteintech 66009-1-Ig 1:5000 WB
Tubulin Cell Signaling Technology 2144 1:5000 WB
Pacific Blue anti-mouse Perforin Biolegend 154407 1:50 FACS
Pacific Blue anti-human/mouse Granzyme B Biolegend 515407 1:50 FACS, IF
PE anti-mouse CD45 Biolegend 103106 1:100 FACS
FITC anti-mouse CD3 Biolegend 100306 1:100 FACS
APC anti-mouse CD8a Biolegend 100712 1:50 FACS
Hoechst Invitogen H1399
Alexa Fluor 647 goat anti-rabbit IgG Invitrogen A-21245
Alexa Fluor 555 goat anti-rabbit IgG Invitrogen A-21434
Alexa Fluor® 488 goat anti-rabbit IgG (H+L) Invitrogen A-11008
Goat anti-rat IgG HRP-linked ThermoFisher 31470
Goat anti-rabbit IgG HRP-linked Invitrogen A-11034
Goat anti-mouse IgG HRP-linked Fisher Scientific 31430
InVivoMAb anti-mouse CD8α BioXCell BE0004-1

Validation

All antibodies are well-recognized clones in the field and validated by the manufacturers.

Eukaryotic cell lines

Policy information about cell lines and Sex and Gender in Research

Cell line source(s)

Mouse GC cell lines (KP and CKP; Mus musculus, male and female) were established from genetically engineered mouse

Cell line source(s)	models (GEMMs) as described in the Methods section. Human GC cell line (AGS; Homo sapiens, female, ATCC CRL-1739, KATOII; Homo sapiens, male, ATCC HTB-103; SNU-16; Homo sapiens, female, ATCC CRL-5974; SNU-5; Homo sapiens, female, ATCC CRL-5973; NCI-N87; Homo sapiens, male, ATCC CRL-5822; MKN45; Homo sapiens; female, provided from Dr. Mazur at MDACC; SNU-1; Homo sapiens, male, ATCC CRL-5971) and embryonic kidney cell line (HEK293T; Homo sapiens, female; ATCC CRL-3216) were obtained from the American Type Culture Collection (ATCC).
Authentication	Human cell lines were authenticated by short tandem repeat (STR) profiling by ATCC prior to use. Murine lines were confirmed by genotyping and morphological verification.
Mycoplasma contamination	Cell lines have been routinely tested and confirmed negative for mycoplasma contamination
Commonly misidentified lines (See ICLAC register)	No commonly misidentified cell lines listed in the ICLAC register were used in this study.

Animals and other research organisms

Policy information about studies involving animals; ARRIVE guidelines recommended for reporting animal research, and Sex and Gender in Research

Laboratory animals	C57BL/6 mice were purchased from The Jackson Laboratory.<br>For in vivo tumor studies, orthotopic gastric injections were performed, followed by pharmacological treatment to assess therapeutic efficacy.
Wild animals	Not applicable.
Reporting on sex	Both male and female mice were used.
Field-collected samples	This study did not involve any field-collected samples.
Ethics oversight	All mice were maintained in compliance with the guidelines of the Institutional Animal Care and Use Committee of the University of Texas MD Anderson Cancer Center. All animal procedures were performed based on the guidelines of the Association for the Assessment and Accreditation of Laboratory Animal Care and institutional (MD Anderson) approved protocols (protocol number 00002587-RN00). This study was compliant with all relevant ethical regulations regarding animal research.

Note that full information on the approval of the study protocol must also be provided in the manuscript.

Clinical data

Policy information about clinical studies

All manuscripts should comply with the ICMJE guidelines for publication of clinical research and a completed CONSORT checklist must be included with all submissions.

Clinical trial registration	Not applicable. This study did not involve a prospective clinical trial.
Study protocol	This study was a retrospective, observational analysis of archived surgical specimens and clinicopathological data, conducted under institutional IRB approval (IRB No. 4-2014-0638).
Data collection	Data collection included clinicopathological variables such as TNM stage, WHO and Lauren classifications, EBV and MSI status, PD-L1 expression (22C3; TPS and CPS), and HER2 status.
Outcomes	The primary outcomes were the expression levels of CRACD and PD-L1 in tumor tissues as assessed by immunohistochemistry, and their associations with histological subtype and clinicopathological features.

Plants

Seed stocks	Not applicable. This study did not involve plants or plant-derived materials.
Novel plant genotypes	Not applicable.
Authentication	Not applicable.

Flow Cytometry

Plots

Confirm that:

☒ The axis labels state the marker and fluorochrome used (e.g. CD4-FITC).

☒ The axis scales are clearly visible. Include numbers along axes only for bottom left plot of group (a 'group' is an analysis of identical markers).

☒ All plots are contour plots with outliers or pseudocolor plots.

☒ A numerical value for number of cells or percentage (with statistics) is provided.

Methodology

Sample preparation	Details are descried in the method section.
Instrument	Flow cytometry data were acquired using an Attune NxT Flow Cytometer (Thermo Fisher Scientific).
Software	Data were collected using Attune NxT software (Thermo Fisher Scientific) and analyzed using FlowJo software (version 10, BD Biosciences).
Cell population abundance	Cell populations were gated based on CD45+ expression.
Gating strategy	Sequential gating was applied using FSC/SSC to exclude debris and doublets, followed by $CD45^{+}$ selection for immune populations. $CD3^{+}$ T cells were subsequently separated into $CD4^{+}$ and $CD8^{+}$ subsets. Representative gating strategy and cut-off values are shown in Supplementary Figure 8.

☒ Tick this box to confirm that a figure exemplifying the gating strategy is provided in the Supplementary Information.
