# Supplementary material for: CRACD loss drives hypoxia-induced immune escape in gastric cancer
Source: Nat Commun. 2026 Sep 1;17:10418. doi: 10.1038/s41467-026-77348-9 (PMC13631292; doi:10.1038/s41467-026-77348-9)
Supplement: Supplementary file 4 [file 41467_2026_77348_MOESM5_ESM.pdf]

nature portfolio

Peer Review File

CRACD loss drives hypoxia-induced immune escape in gastric cancer

Corresponding Author: Dr Jae-Il Park

This file contains all reviewer reports in order by version, followed by all author rebuttals in order by version.

Attachments originally included by the reviewers as part of their assessment can be found at the end of this file.

Version 0:

Reviewer comments:

Reviewer #1

(Remarks to the Author)
This study presents an interesting mechanism by which CRACD loss promotes mucinous differentiation and immune evasion in gastric cancer through activation of a HIF1α–PD-L1 signaling axis. The combination of organoid models, orthotopic tumor models, and single-cell transcriptomic analyses provides a strong framework for investigating this process. In particular, the clinical validation using patient-derived organoids and tissue microarrays enhances the translational relevance of the findings. Despite these strengths, several mechanistic aspects of the study require further clarification and experimental support.

Comments :
1. The source and mechanism of ROS accumulation remain insufficiently defined.
The authors propose that CRACD loss leads to actin dysregulation, which subsequently induces ROS accumulation and activates HIF1α signaling. However, intracellular ROS can arise from multiple sources, including mitochondrial dysfunction, NADPH oxidases, and inflammatory signaling pathways. The current study mainly demonstrates the presence of ROS but does not clearly define its origin. A more detailed investigation of the mechanism leading to ROS accumulation would strengthen the proposed signaling cascade.

2. The validation of the COX-NOX signaling axis appears relatively limited.
The manuscript suggests that CRACD loss activates NF-κB/COX2 signaling, which in turn stimulates NADPH oxidase–dependent ROS production. However, the evidence supporting this pathway is primarily based on pharmacological inhibition. Additional experimental validation, such as genetic perturbation or more direct functional assays targeting key components of the COX-NOX pathway, would help substantiate this mechanistic link.

3. The causal relationship between HIF1α and PD-L1 requires more definitive evidence.
The authors demonstrate that pharmacological inhibition of HIF1α reduces PD-L1 expression. While this finding is suggestive, the use of chemical inhibitors alone may not fully establish a direct regulatory relationship. Incorporating a genetic approach, such as HIF1α knockout or knockdown models, would provide stronger evidence that HIF1α directly regulates PD-L1 expression in this context.

4. The therapeutic experiments raise the possibility of combinational treatment strategies.
In the current study, both HIF1α inhibition and anti-PD-L1 treatment individually showed strong anti-tumor effects. Given that these interventions target different points within the same pathway, it would be interesting to determine whether a combinational treatment strategy might produce additive or synergistic therapeutic benefits. Exploring this possibility could further enhance the translational significance of the study.

Reviewer #2

(Remarks to the Author)
In this study, Seo et. al. examine the function of CRACD and the consequences of its inactivation in gastric cancer. The

authors conduct a detailed analysis on the cell intrinsic and extrinsic effects of CRACD loss including actin dysregulation, ROS generation through activated COX2 signaling, increased PDL1 expression and consequent immunosuppression. They also describe an association between mucinous differentiation and CRACD loss. The work is extensive and integrates findings across organoid models, in vivo studies and investigating patient samples. Molecular mechanisms driving gastric cancer progression are critical to study and this work is an important advance. However, several major issues reduce my enthusiasm about the manuscript in its current form.
Major:
1. The manuscript is extremely dense. For instance, figures 4 and 6 have panels from A to R and Fig. 5 from A-Z. Rearranging this material by keeping only what is critical to the story in the main figures and using the rest in supplementary figures will be helpful to the readers. This will also refine the focus of the manuscript, which right now extends too broadly.
2. There is confusion in the clinical associations made. Several times in the text, a major claim is that CRACD loss is related to the mucinous subtype of gastric cancer. However, Tp53 and KRAS loss models intestinal type of gastric cancer. Scissor correlations are made with diffuse gastric cancer. In Supplementary figure 1, some gastric cancer subtypes show amplifications in CRACD. Presumably, this would be an opposite phenotype compared to the loss of function effects described in the manuscript. The TMA studies also seem to study gastric cancer as a whole and not in a mucinous subtype. Given the rarity of and controversies in mucinous gastric cancer identification, prevalence and prognosis (see for example doi 10.1007/s11605-023-05853-z), I am not sure if finding a clean line of evidence between CRACD loss and mucinous GC is possible or even required. These attempts at linking a potentially strong pre-clinical story to clinical relevance weaken rather than strengthen the manuscript.
3. scRNA-seq analysis has several issues. This again weakens a potentially strong mechanistic story.
• Please clarify the actual experimental replicates used in these experiments from both organoids and mice.
• There are no statistical comparisons made anywhere for cell proportions (cannot be done with one replicate), gene expression, regulons, etc. The current results are often described using terms like 'significant', 'enriched', 'relatively', 'increased', 'decreased' but no evidence is provided for this, with results presented as feature plots or dot plots.
• Given the above point, it is difficult to assess the majority of these results- but from the plots it does not seem like there are major differences in WT and KO organoids.
• Since you identified a proliferative cluster, it is not surprising that this is the cluster that has an association with bulk RNA-seq data using Scissor. It is unclear why diffuse GC was chosen.
• I do not see how "These results suggest that CRACD depletion induces cell plasticity that drives the transcriptional signature of gastric neoplasia" is a conclusion that can be reached with the current analysis including the Phate maps.
Minor:
1. The authors mention prior studies linking CRACD loss with neuroendocrine differentiation. This would be interesting to discuss if regulating plasticity is a core function of this gene, how it is achieved (dependent on the actin mechanism?) and if it differs between organs. Similarly, is the link between mucinous differentiation also seen in these other organs? In the germline KO mouse, does CRACD loss result in similar mucinous/cell plasticity phenotypes elsewhere?
2. Fig. 3E seems to be missing.
3. Fig. 3J-L compares CKP tumors to KP but I could not find the data for KP.

Reviewer #3

(Remarks to the Author)
In this manuscript, the authors demonstrate that loss of the actin regulator CRACD drives mucinous plasticity and immune evasion in gastric cancer. Using genetically engineered murine gastric organoids with CRACD ablation combined with Kras mutation and Trp53 loss, they show that CRACD deficiency disrupts epithelial integrity and promotes the development of mucinous gastric adenocarcinoma features. Mechanistically, the authors reveal that CRACD loss leads to actin dysregulation, which activates NF-κB/COX-2 signaling and NADPH oxidase–dependent ROS production. The resulting oxidative stress stabilizes HIF1α and induces PD-L1 transcription, thereby suppressing anti-tumor immunity. Furthermore, pharmacologic inhibition of HIF1α or PD-L1 restores immune surveillance and reduces tumorigenesis. Consistent with these findings, analyses of patient-derived organoids and tissue microarrays show an inverse correlation between CRACD and PD-L1 expression. These results suggest that CRACD inactivation is a key driver of mucinous plasticity and immune escape in gastric cancer and highlight the HIF1α–PD-L1 axis as a potential therapeutic target in CRACD-deficient tumors.

The concept of this study is interesting and potentially impactful. The authors provide a solid mechanistic characterization of CRACD loss in gastric tumorigenesis. However, the clinical significance of these findings requires further clarification, and additional studies will be needed to determine whether these findings can translate into meaningful therapeutic advances.

The specific comments are listed below.

Comments

1. How does the effect of PX-478 compare with anti-PD-L1 therapy alone? Based on the presented data, CRACD-negative (CRACD-loss) gastric cancers appear to respond relatively well to anti-PD-L1 monotherapy. Therefore, it would be important to clarify whether the use of the HIF-1α inhibitor PX-478 provides additional benefit compared with the currently available anti-PD-L1 therapy. The potential therapeutic advantage of introducing PX-478 in this context should be discussed.

2. Is there any association between CRACD-loss and established molecular subtypes of gastric cancer? Gastric cancer is known to consist of distinct molecular subtypes, which is strongly related to immune microenvironment and response to immunotherapy. If CRACD is proposed as a biomarker-guided candidate for PD-L1 blockade, it is important to determine

whether its predictive value is independent of established biomarkers. At least this point should be discussed.

3. It would be informative to examine whether CRACD-loss is associated with clinical outcomes, such as progression-free survival or overall survival, in gastric cancer cohorts. Such analysis would help clarify the clinical relevance of CRACD inactivation.

4. In Figure 7B, the stratification into diffuse and intestinal types may potentially mislead readers. Based on Supplementary Figure 1, CRACD inactivation appears to be more frequent in the intestinal type rather than the diffuse type. In addition, diffuse-type gastric cancer is generally considered less responsive to immune checkpoint inhibitors. If the main purpose of this figure is to demonstrate the relationship between CRACD status and PD-L1 signaling, it is unclear whether presenting the histological subtype classification is necessary.

Version 1:

Reviewer comments:

Reviewer #1

(Remarks to the Author)
The authors have responded adequately to the concerns from reviewer.

Reviewer #2

(Remarks to the Author)
The authors have addressed my previous comments and concerns to a reasonable extent.

Reviewer #3

(Remarks to the Author)
The authors have addressed my concerns in their response and through additional discussion and analysis, making the data more robust.

Open Access This Peer Review File is licensed under a Creative Commons Attribution 4.0 International License, which permits use, sharing, adaptation, distribution and reproduction in any medium or format, as long as you give appropriate credit to the original author(s) and the source, provide a link to the Creative Commons license, and indicate if changes were made.
In cases where reviewers are anonymous, credit should be given to 'Anonymous Referee' and the source.
The images or other third party material in this Peer Review File are included in the article’s Creative Commons license, unless indicated otherwise in a credit line to the material. If material is not included in the article’s Creative Commons license and your intended use is not permitted by statutory regulation or exceeds the permitted use, you will need to obtain

permission directly from the copyright holder.
To view a copy of this license, visit https://creativecommons.org/licenses/by/4.0/

Responses to Comments

Reviewer #1

"This study presents an interesting mechanism by which CRACD loss promotes mucinous differentiation and immune evasion in gastric cancer through activation of a HIF1α–PD-L1 signaling axis. The combination of organoid models, orthotopic tumor models, and single-cell transcriptomic analyses provides a strong framework for investigating this process. In particular, the clinical validation using patient-derived organoids and tissue microarrays enhances the translational relevance of the findings. Despite these strengths, several mechanistic aspects of the study require further clarification and experimental support."

Major points

A1. "*The source and mechanism of ROS accumulation remain insufficiently defined. The authors propose that CRACD loss leads to actin dysregulation, which subsequently induces ROS accumulation and activates HIF1α signaling. However, intracellular ROS can arise from multiple sources, including mitochondrial dysfunction, NADPH oxidases, and inflammatory signaling pathways. The current study mainly demonstrates the presence of ROS but does not clearly define its origin. A more detailed investigation of the mechanism leading to ROS accumulation would strengthen the proposed signaling cascade."*

We appreciate your important comments. We agree that defining the source and mechanism of ROS accumulation is essential for strengthening the proposed CRACD-ROS-HIF1α signaling cascade. To address the comment, we performed additional experiments: (i) Determine whether CRACD-mediated actin dysregulation is sufficient to induce ROS accumulation, (ii) Identify the predominant source of ROS, and (iii) Further validate the involvement of NOX1 in this process. We focused on NOX1 because our scRNA-seq dataset identified *Nox1* as the only detectable NADPH oxidase gene expressed in the gastric epithelial tumor cells (**Supplementary Fig. 10D**).

a. **Actin dysregulation**. To determine whether actin dysregulation is sufficient to promote ROS accumulation, we compared ROS levels in cells expressing wild-type CRACD versus the ΔCPI mutant, which is defective in capping protein interaction and fails to properly regulate actin dynamics. Consistent with our model, disruption of the CPI domain was associated with increased ROS accumulation, supporting that impaired actin regulation is an upstream driver of oxidative stress (**Supplementary Fig. 8A-C**). The functional importance of the CPI domain in regulating actin dynamics has been previously established in our earlier study (Nature Cell Biology 2018) [1], where CRACD was shown to control actin polymerization through its interaction with capping proteins. Building on this, we extended our analysis to additional cellular contexts, including CKP cells and the diffuse-type gastric cancer cell line MKN45, which exhibits low endogenous CRACD expression and carries a nonsense mutation in the *CRACD* allele (**Supplementary Fig. 8B**). In both systems, expression of wild-type CRACD did not induce ROS accumulation, whereas expression of the ΔCPI mutant led to a marked increase in ROS levels, further supporting that disruption of CPI-mediated actin regulation promotes oxidative stress (**Figure 5K, Supplementary Fig. 8C**). Furthermore, we additionally perturbed actin/capping protein function and observed a similar increase in ROS levels. Moreover, to determine whether this effect reflects a broader mechanism of capping protein regulation rather than being specific to CRACD alone, we genetically depleted CARMIL1, a key regulator of capping protein activity. Notably, *Carmil1* depletion resulted in reduced F-actin formation, as evidenced by decreased phalloidin staining, and was accompanied by increased ROS accumulation (**Supplementary Fig. 8D-E**). Collectively, these findings demonstrate that cytoskeletal dysregulation caused by impaired capping protein regulation is sufficient to drive ROS accumulation.

b. **Mitochondrial ROS.** To address the comment, we further investigated the potential source of ROS accumulation. Dihydrohodamine 123 (DHR123), a cell-permeable fluorescent probe that is oxidized by intracellular ROS to rhodamine 123, is commonly used to assess cellular oxidative stress. DHR123 staining revealed no apparent difference between KP and CKP cells (**Fig. R1A, B**).

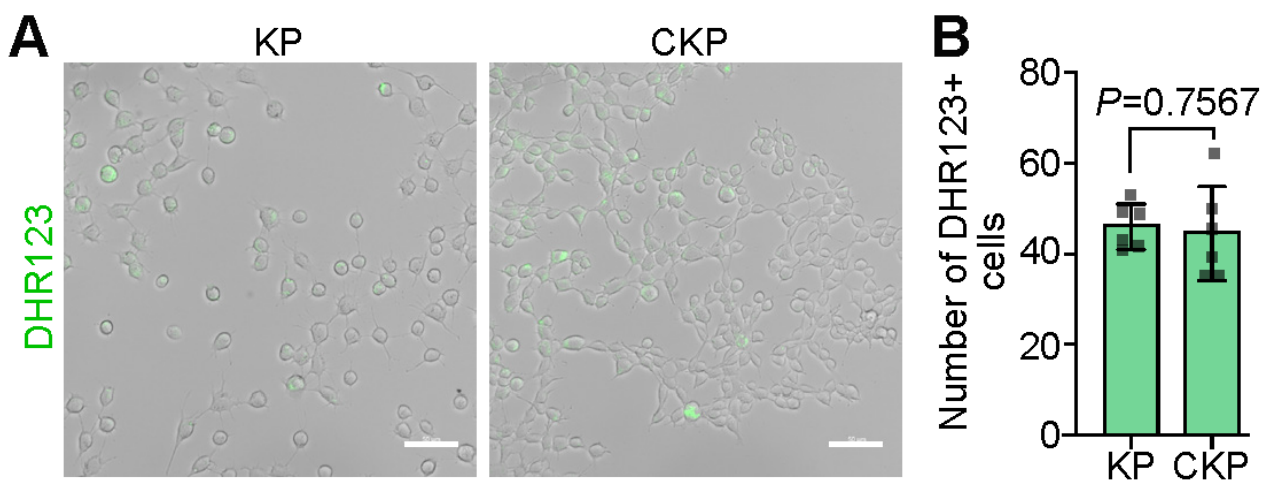

Figure R1. DHR123 staining. (A) Representative fluorescence images of KP and CKP cells stained with DHR123 (green). Scale bars: 50 µm (B) Quantification of DHR123+ fluorescence intensity. Data are presented as mean ± SEM, and statistical significance was determined by Student's *t*-test.

Consistent with this observation, treatment with MitoTEMPO, a mitochondrial ROS scavenger, produced minimal effects on HIF1α expression compared with the marked suppression observed following treatment with diphenyleneiodonium (DPI), an NADPH oxidase inhibitor, or N-acetyl-L-cysteine (NACA), a general ROS scavenger (**Fig. R2**). These findings support a predominantly non-mitochondrial source of ROS in CRACD-deficient cells.

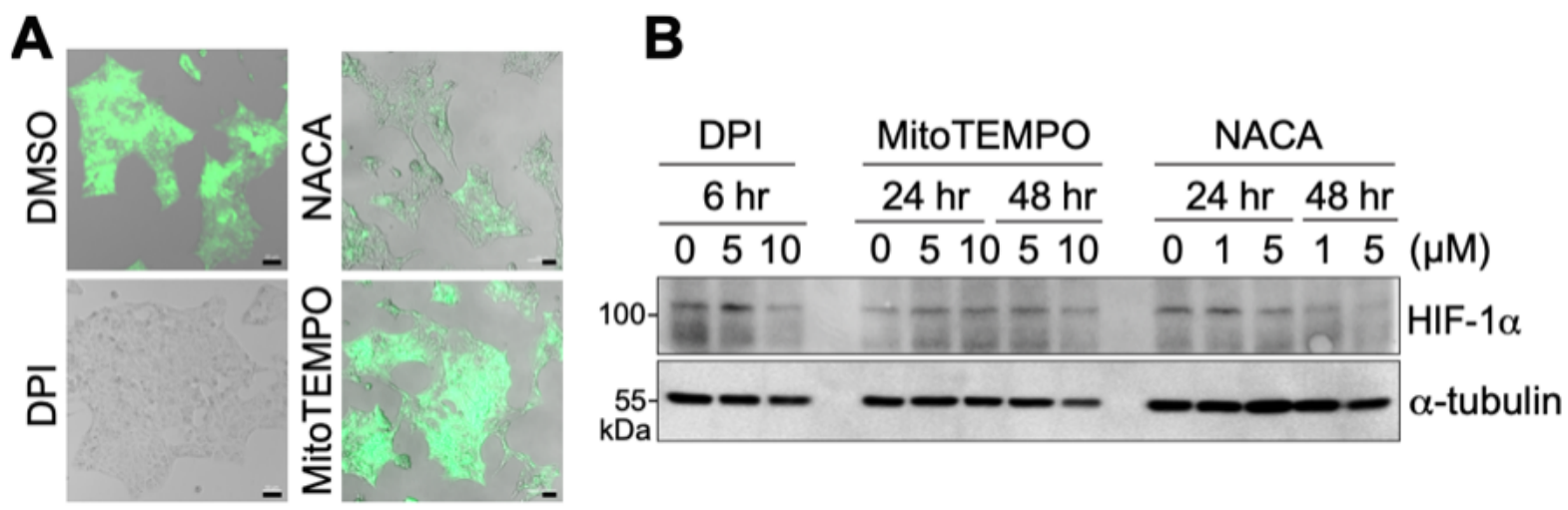

Figure R2. Non-mitochondrial ROS contributes to HIF1α stabilization. (A) Representative ROS staining DCFH-DA images (green) of CKP cells treated with DMSO, DPI (10 µM), NACA (5 µM), or MitoTEMPO (10 µM). Scale bars: 50 µm (B) Immunoblot analysis of HIF1α expression.

c. **NOX1 NADPH oxidase**. To further determine the source of ROS downstream of CRACD loss, we investigated the involvement of the NADPH oxidase pathway using both pharmacological and genetic approaches. As shown in Figure 5, treatment with DPI, an NADPH oxidase inhibitor, markedly reduced intracellular ROS levels and PD-L1 expression in CKP cells, indicating that NADPH oxidase activity contributes to the CRACD-loss phenotype. As kindly suggested, we performed genetic validation of these findings using Nox1-deficient CKP cells, since our scRNA-seq analysis identified Nox1 as a major NADPH oxidase expressed in gastric tumor epithelial cells. Either *Nox1* knock-out (KO) or *Hif1a* KO abolished the expression of PD-L1, while *Hif2a* KO failed to do so (**Fig. R3A**). In line with this, *Nox1* KO significantly reduced intracellular ROS levels as measured by DCFH-DA fluorescence, whereas *Hif1α* or *Hif2α* deletion had minimal effects (**Fig. R3B**), indicating that NOX1 functions upstream of HIF signaling. Notably, DHR-123 fluorescence remained largely unchanged in all three knockout conditions, with only a minor reduction observed following *Nox1* deletion (**Fig. R3C**). These data suggest that the elevated **ROS induced by CRACD loss is primarily derived from NOX1-dependent non-mitochondrial sources rather than mitochondrial ROS production**.

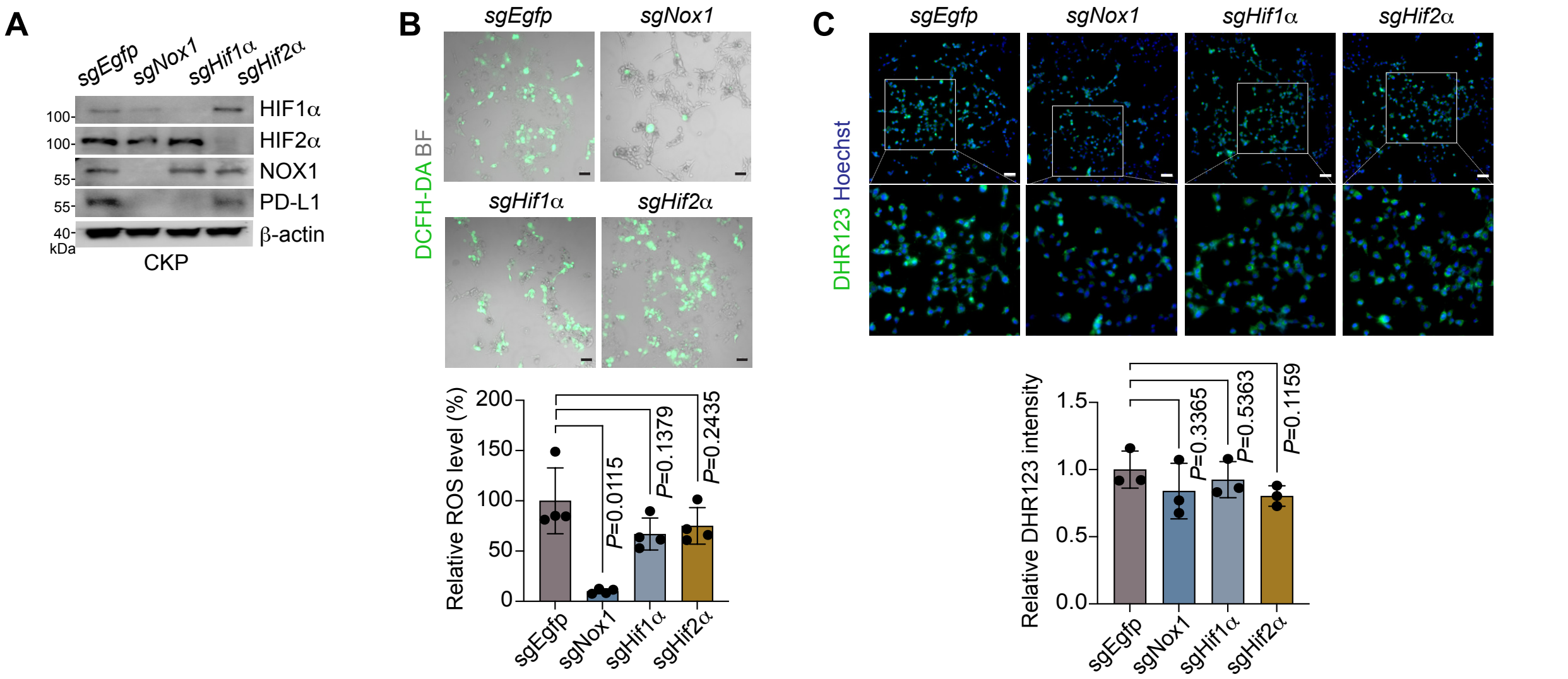

Figure R3. Nox1-dependent ROS production. (A) Immunoblot analysis of HIF1α, HIF2α, NOX1, PD-L1 and β-actin in CKP cells. (B) Representative fluorescence images of DCFH-DA staining (green) in CKP cells. Quantification of DCFH-DA/ROS levels is shown below. Fluorescence intensity was normalized to the sg*Egfp* control group. (C) Representative images of DHR123 staining (green) and Hoechst nuclear staining (blue) in CKP cells. Quantification of DHR123 fluorescence intensity normalized to Hoechst-positive cells is shown below.

We have incorporated these new data into the revised results section (**Supplementary Fig. 10F-H**) and updated the discussion accordingly.

A2. "*The validation of the COX-NOX signaling axis appears relatively limited. The manuscript suggests that CRACD loss activates NF-κB/COX2 signaling, which in turn stimulates NADPH oxidase–dependent ROS production. However, the evidence supporting this pathway is primarily based on pharmacological inhibition. Additional experimental validation, such as genetic perturbation or more direct functional assays targeting key components of the COX-NOX pathway, would help substantiate this mechanistic link.*"

We agree with the reviewer that additional validation of the COX/NOX signaling axis (upstream) would strengthen the mechanistic link. To address this point, we performed genetic perturbation of *Nox1* in CKP cells. As shown in **Figure R3** (above), ***Nox1* depletion significantly reduced ROS accumulation and attenuated downstream HIF1α and PD-L1 expression.**

A3. "*The causal relationship between HIF1α and PD-L1 requires more definitive evidence. The authors demonstrate that pharmacological inhibition of HIF1α reduces PD-L1 expression. While this finding is suggestive, the use of chemical inhibitors alone may not fully establish a direct regulatory relationship. Incorporating a genetic approach, such as HIF1α knockout or knockdown models, would provide stronger evidence that HIF1α directly regulates PD-L1 expression in this context.*"

We thank the reviewer for this important comment. We agree that additional evidence is required to support a direct regulatory relationship between HIF1α and PD-L1. To address this point, we added genetic perturbation experiments targeting *Hif1α* and *Hif2α/Epas1* genes (**Fig. R4**). Notably, **Hif1α depletion markedly reduced PD-L1 protein expression, whereas Hif2α depletion had minimal effects** on PD-L1 levels, indicating that HIF1α rather than HIF2α regulates PD-L1 expression in CKP cells. This result was added to the revised manuscript (**Supplementary Fig. 10F**).

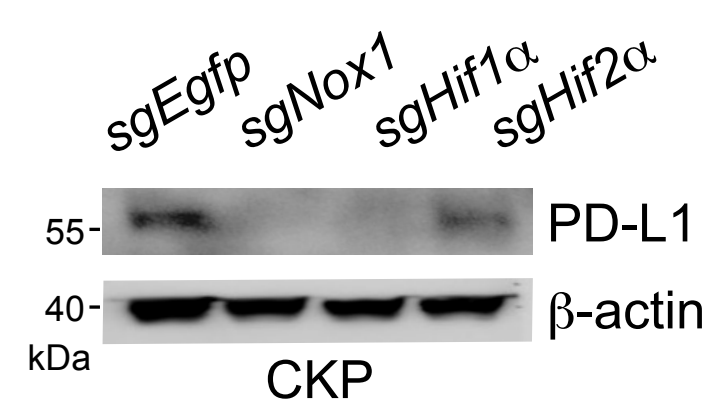

Figure R4. Immunoblot analysis of PD-L1. PD-L1 expression in CKP cells following deletion of *Nox1*, *HIf1α*, or *HIf2α*.

A4. "*The therapeutic experiments raise the possibility of combinational treatment strategies. In the current study, both HIF1α inhibition and anti-PD-L1 treatment individually showed strong anti-tumor effects. Given that these*

interventions target different points within the same pathway, it would be interesting to determine whether a combinational treatment strategy might produce additive or synergistic therapeutic benefits. Exploring this possibility could further enhance the translational significance of the study."

We thank the reviewer for the insightful suggestion. We agree that determining whether the combination of PX-478 and an anti-PD-L1 antibody targeting the same pathway has an additive or synergistic effect would be valuable for therapeutic applications, given the frequent failure of monotherapy in clinical applications and the limitations of our syngeneic murine models. Unfortunately, **either PX-478 or the anti-PD-L1 antibody alone was sufficient to eliminate tumors** (**Fig. 6H-K**). Notably, this finding was somewhat unexpected, as diffuse-type and mucinous GCs are generally considered less responsive to immune checkpoint blockade. **Therefore, experimentally determining their additive or synergistic effect in our preclinical models was not feasible**. Nevertheless, anti-PD-L1 treatment alone elicited substantial tumor suppression in our CRACD-deficient model, suggesting that **CRACD loss may identify a subset of GCs with increased susceptibility to immune-targeted intervention**.

Importantly, our clinical analyses demonstrated that patients with low or absent CRACD expression tended to exhibit higher PD-L1 CPS scores (**Fig. 7**), indicating that caution is warranted when extrapolating these preclinical findings directly to clinical practice. Therefore, as insightfully pointed out by the reviewer, while the strong efficacy of anti-PD-L1 monotherapy observed in our mouse model suggests that combination treatment may not be required to achieve initial tumor control, clinical management would likely benefit from combination or multimodal therapeutic strategies rather than immune checkpoint blockade alone.

Notably, long-term follow-up revealed tumor relapse after treatment discontinuation. Anti-PD-L1-treated tumors recurred relatively shortly after treatment withdrawal, whereas relapse following PX-478 treatment was observed after a prolonged interval of approximately **one year** and was accompanied by liver metastasis (**Fig. R5**). These observations suggest that **both HIF1α inhibition and PD-L1 blockade effectively suppress primary tumor growth, but neither approach alone is sufficient to completely prevent disease recurrence**.

Therefore, rather than improving initial tumor regression, combined or sequential targeting of HIF1α and PD-L1 may be particularly valuable for achieving durable disease control and preventing relapse or metastatic progression. While combination studies were beyond the scope of the current work, our findings provide a rationale for future investigations focused on long-term treatment durability and recurrence prevention in CRACD-deficient gastric cancer. As suggested, we have added additional discussion to clarify this point in the revised manuscript.

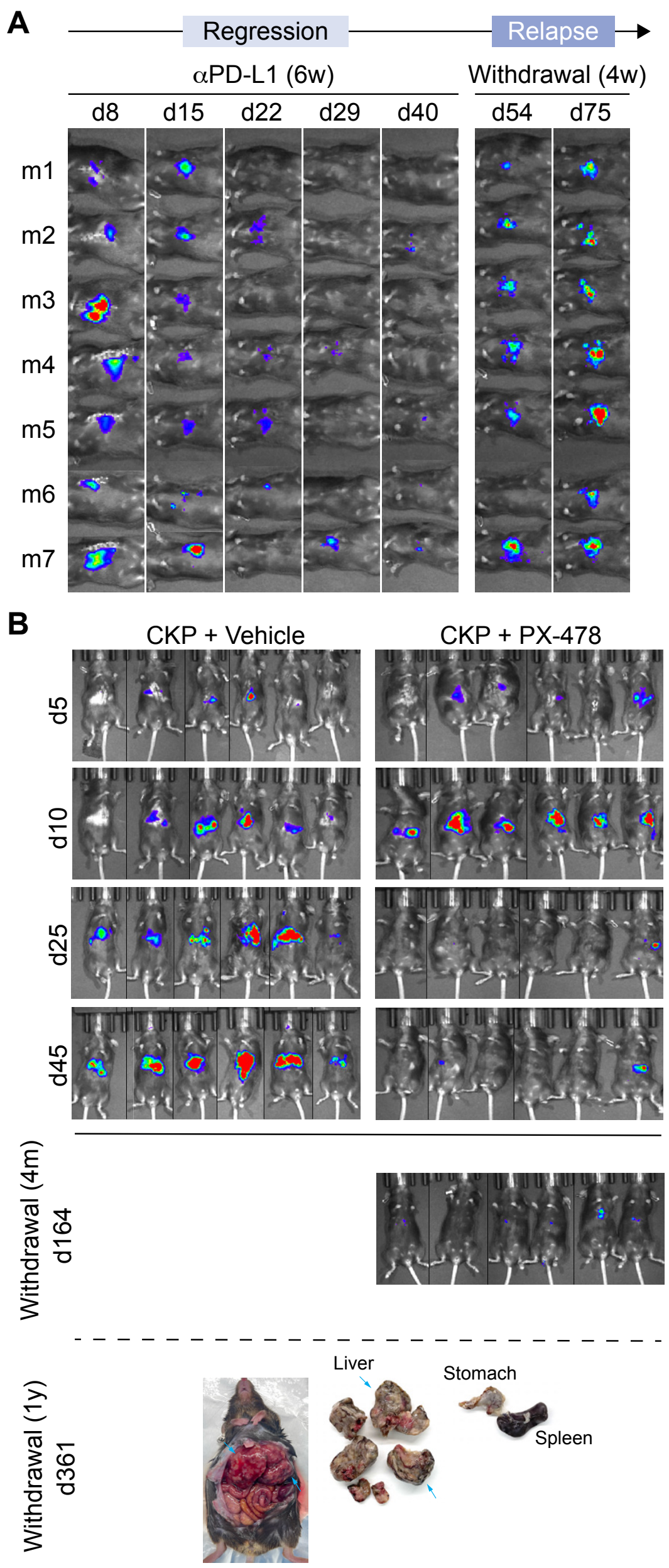

Figure R5. Anti-PD-L1 and HIF1a inhibition (PX-478) suppresses tumor growth but do not prevent tumor recurrence. (A) Longitudinal IVIS imaging of CKP orthotopic tumors treated with anti-PD-L1 for 6 weeks, followed by treatment withdrawal. Tumor regression was observed during treatment, whereas recurrent tumor growth emerged after treatment discontinuation. (B) IVIS imaging with PX-478 or vehicle. PX-478 suppressed primary tumor growth; however, late recurrence and liver metastasis were observed (day 361) following prolonged treatment withdrawal. Representative images are shown.

Again, we appreciate all constructive and insightful comments.

Reviewer #2

"In this study, Seo et. al. examine the function of CRACD and the consequences of its inactivation in gastric cancer. The authors conduct a detailed analysis on the cell intrinsic and extrinsic effects of CRACD loss including actin dysregulation, ROS generation through activated COX2 signaling, increased PDL1 expression and consequent immunosuppression. They also describe an association between mucinous differentiation and CRACD loss. The work is extensive and integrates findings across organoid models, in vivo studies and investigating patient samples. Molecular mechanisms driving gastric cancer progression are critical to study and this work is an important advance. However, several major issues reduce my enthusiasm about the manuscript in its current form."

Major points

B1. "*The manuscript is extremely dense. For instance, figures 4 and 6 have panels from A to R and Fig. 5 from A-Z. Rearranging this material by keeping only what is critical to the story in the main figures and using the rest in supplementary figures will be helpful to the readers. This will also refine the focus of the manuscript, which right now extends too broadly."*

As suggested, in the revised version, we have carefully restructured the figures to enhance readability and to better focus the main narrative.

B2. "*There is confusion in the clinical associations made. Several times in the text, a major claim is that CRACD loss is related to the mucinous subtype of gastric cancer. However, Tp53 and KRAS loss models intestinal type of gastric cancer. Scissor correlations are made with diffuse gastric cancer. In Supplementary figure 1, some gastric cancer subtypes show amplifications in CRACD. Presumably, this would be an opposite phenotype compared to the loss of function effects described in the manuscript. The TMA studies also seem to study gastric cancer as a whole and not in a mucinous subtype. Given the rarity of and controversies in mucinous gastric cancer identification, prevalence and prognosis (see for example doi 10.1007/s11605-023-05853-z), I am not sure if finding a clean line of evidence between CRACD loss and mucinous GC is possible or even required. These attempts at linking a potentially strong pre-clinical story to clinical relevance weaken rather than strengthen the manuscript."*

Thank you for your crucial comments. We agree with the limitations of our syngeneic model and the KP genetic foundation.

First, we would like to clarify that the association between CRACD loss and gastric cancer subtypes is not restricted to a single histological category. In our genetically engineered mouse models, CRACD-deficient tumors (CKP) **do not exclusively exhibit a homogenous mucinous/diffuse-type phenotype**, but rather display **mixed** histological features, including both diffuse and intestinal characteristics. This is consistent with the known plasticity and heterogeneity of gastric cancer subtypes.

Second, in the Scissor analysis, KP tumors exhibit limited enrichment for DGAC-like features, whereas CRACD-deficient tumors (C and CKP) show significantly **greater enrichment of DGAC-associated signatures**. Specifically, the DGAC-like transcriptional signature is more prominently enriched in C and CKP tumors, whereas KP tumors exhibit only a modest representation of this phenotype. These results suggest that CRACD loss enhances the diffuse-like transcriptional program rather than exclusively defining it.

Third, analysis of human gastric adenocarcinoma datasets revealed that **CRACD alterations are present across multiple gastric cancer subtypes, including diffuse-type tumors**. Consistent with our mouse studies, CRACD-low tumors exhibited gene expression programs associated with cellular plasticity and aggressive tumor behavior.

Fourth, CRACD gene amplification is also observed in DGAC (< 2%) (Supplementary Figure 1), which might be inconsistent with our working model. However, given that actin dynamics should be tightly controlled and maintained at a steady state, both loss- and gain-of-function of CRACD or actin regulators are highly likely to lead to deregulated cell plasticity and signaling pathways.

Together, our data suggest that CRACD loss contributes to the activation of diffuse/mucinous-like transcriptional and phenotypic programs but does not define a strict subtype-specific identity. **We added such clarification in the revised manuscript**.

As also pointed out, K (*KRAS/Kras*) is known to be associated with intestinal-type gastric cancer (IGC), which may imply that RAS/MAPK signaling hyperactivation is specific to IGC but not to other gastric cancer types, including DGC or mucinous. However, we recently observed that IGC driven by KP becomes diffuse-type gastric cancer (DGC) with additional E-cadherin/Cdh1 loss [2] (JEM, 2024), which suggests that non-IGC subtypes might also be linked to RTK-RAS/MAPK signaling hyperactivation without *KRAS* or *BRAF* oncogenic mutations.

To further address this, we analyzed genomic alterations in RAS/MAPK pathway-associated genes in mucinous gastric cancer and IGC using publicly available datasets (**Fig. R6**). We found that oncogenic mutations affecting multiple components of the RAS/MAPK pathway were frequently observed in both subtypes. These genes include *ERBB* family members, *EGFR*, *KRAS*, *NF1*, *RAF* family genes, and MAPK pathway regulators. Importantly, mucinous gastric cancers exhibited an overall RAS/MAPK mutational burden comparable to that observed in IGC, indicating that **activation of the RTK-RAS/MAPK pathway is a common molecular feature across gastric cancer subtypes**. While the specific mutational spectrum varied between subtypes, these findings support the biological relevance of RAS/MAPK pathway activation in mucinous gastric cancer. Collectively, these data support the use of a KRAS-driven model as a biologically relevant platform to investigate how CRACD loss cooperates with RAS/MAPK signaling to promote tumor progression and lineage plasticity.

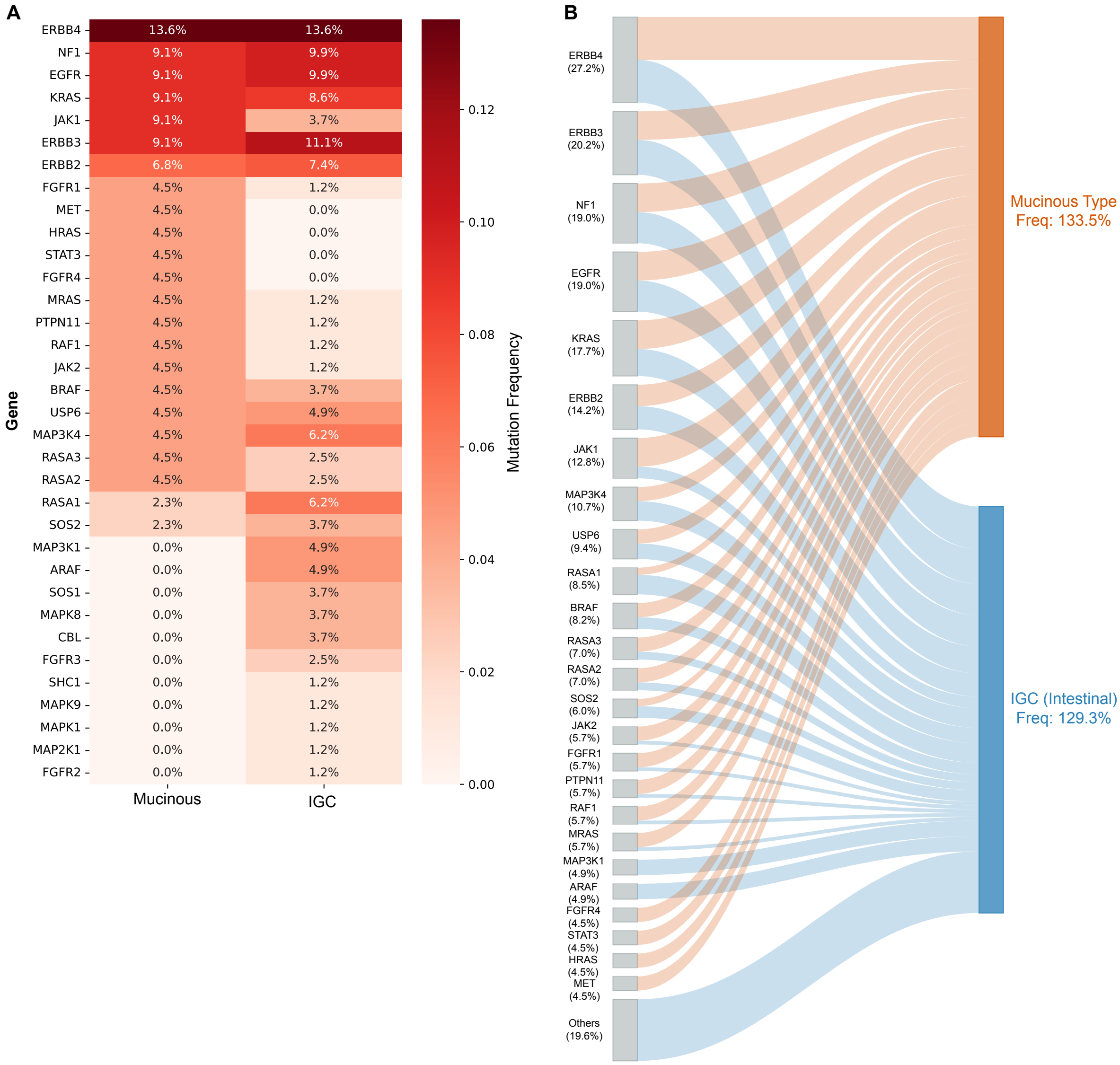

Figure R6. Comparative analysis of RAS/MAPK pathway-associated gene alterations in mucinous gastric cancer (Mucinous) and intestinal-type gastric cancer (IGC). (A) Frequency of genomic alterations in RAS/MAPK pathway-associated genes in diffuse gastric adenocarcinoma (DGA; red) and intestinal gastric cancer (IGC; blue). Genes are ranked according to alteration frequency within each subtype. Among 8 gastric cancer datasets deposited in cBioPortal, DGA (171 samples; including signet-ring cell carcinoma) and IGC (81 samples) were examined for gene mutation analysis. (B) Sankey plot illustrating the contribution of individual RAS/MAPK pathway-associated genes to the cumulative alteration frequency in DGA (red) and IGC (blue). Percentages indicate the alteration frequency for each gene and the total cumulative alteration frequency for each subtype. Genes encoding RAS/MAPK signaling components are mutated in both DGA and IGC, indicating that RAS/MAPK signaling hyperactivation serves as a universal driver of tumorigenesis. Mutations in downstream molecules (MAPK) are common, whereas upstream mutations are subtype specific. For instance, *NRAS* and *MET* mutations are exclusive to DGA, whereas ERBBs are more dominantly mutated in IGC than in DGA.

B3. "*scRNA-seq analysis has several issues. This again weakens a potentially strong mechanistic story.*
- *Please clarify the actual experimental replicates used in these experiments from both organoids and mice.*
- *There are no statistical comparisons made anywhere for cell proportions (cannot be done with one replicate), gene expression, regulons, etc. The current results are often described using terms like 'significant', 'enriched', 'relatively', 'increased', 'decreased' but no evidence is provided for this, with results presented as feature plots or dot plots.*
- *Given the above point, it is difficult to assess the majority of these results- but from the plots it does not seem like there are major differences in WT and KO organoids.*
- *Since you identified a proliferative cluster, it is not surprising that this is the cluster that has an association with bulk RNA-seq data using Scissor. It is unclear why diffuse GC was chosen.*
- *I do not see how "These results suggest that CRACD depletion induces cell plasticity that drives the transcriptional signature of gastric neoplasia" is a conclusion that can be reached with the current analysis including the Phate maps."*

We appreciate your critical comments regarding the scRNA-seq analysis and agree that clarification and improved presentation were needed. We have addressed each concern point-by-point below.

- *"Please clarify the actual experimental replicates used in these experiments from both organoids and mice."*

We have now explicitly clarified the experimental design and biological replicates used for the scRNA-seq experiments in the revised manuscript. Both the organoid- and mouse-derived scRNA-seq datasets were generated from pooled biological samples. Pooling was necessary because individual organoids and, particularly, individual KP tumors yielded insufficient numbers of viable cells for robust single-cell library preparation. This experimental design has now been clearly described in the Methods and figure legends to avoid ambiguity, as we recently performed [2-6].

- *"There are no statistical comparisons made anywhere for cell proportions (cannot be done with one replicate), gene expression, regulons, etc. The current results are often described using terms like 'significant', 'enriched', 'relatively', 'increased', 'decreased' but no evidence is provided for this, with results presented as feature plots or dot plots."*

We agree that pooled scRNA-seq datasets do not permit comparisons between biological replicates. Accordingly, we have revised the manuscript to present these analyses as descriptive rather than inferential. Throughout the manuscript, terms such as "significant," "enriched," or "increased" have been replaced with more appropriate descriptive language. We have also added a discussion of these statistical limitations in the Discussion, including the limited sample size and cohort heterogeneity.

- *"Given the above point, it is difficult to assess the majority of these results- but from the plots it does not seem like there are major differences in WT and KO organoids."*

We agree that the observed transcriptional differences are modest and should not be overinterpreted. Therefore, we have revised the manuscript to present the scRNA-seq data as supportive evidence rather than primary mechanistic evidence. To improve readability, the previous Figure 2, containing the extensive scRNA-seq and bulk-RNA seq analyses, has been moved to the **Supplementary Figure 3**.

- *"Since you identified a proliferative cluster, it is not surprising that this is the cluster that has an association with bulk RNA-seq data using Scissor. It is unclear why diffuse GC was chosen."*

We thank the reviewer for pointing this out. We agree that the association between the proliferative cluster and the bulk RNA-seq phenotype is not specific to diffuse GC. In response to this comment, we re-ran the Scissor analysis using the entire TCGA-STAD cohort rather than the diffuse GC subset. We observed a similar enrichment of matched cells in the proliferative cell cluster (**Fig. R7**). This result indicates that the previous Scissor-based association was largely driven by the proliferative state of the cell cluster rather than by a diffuse GC-specific feature. We therefore agree with the reviewer's concern and have removed this analysis and the related interpretation from the revised manuscript. We appreciate the reviewer's careful assessment, which helped us avoid overinterpreting this result.

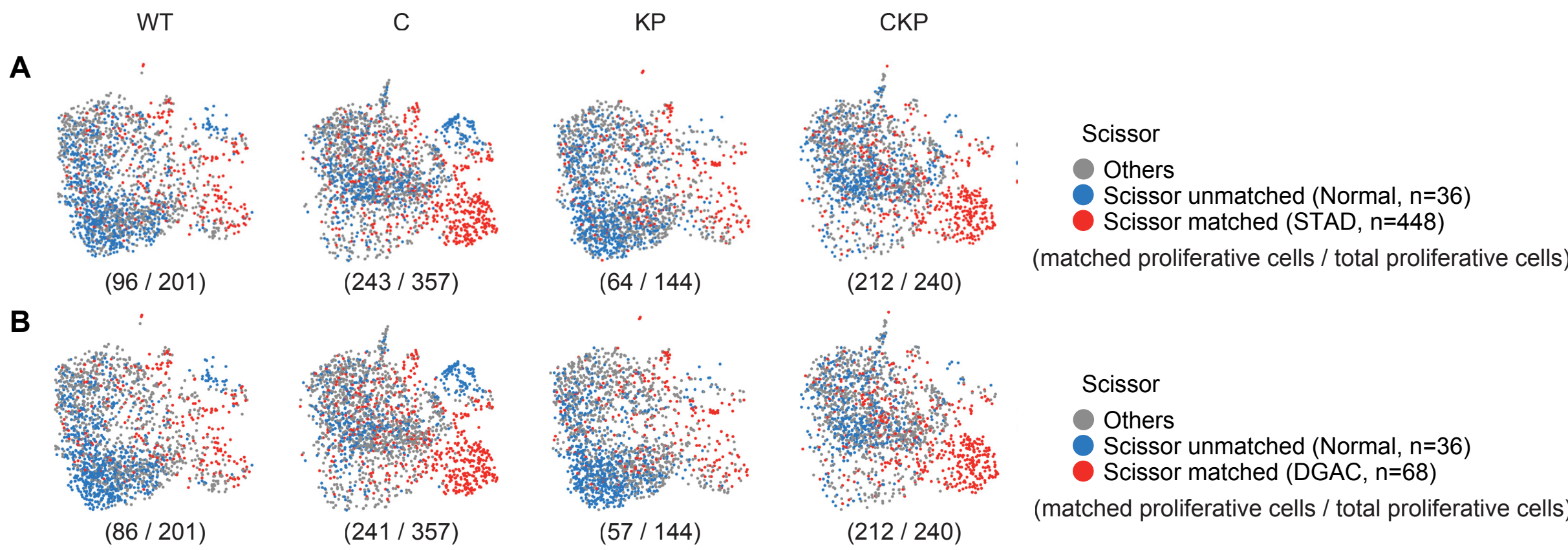

Fig. R7. Scissor mapping of TCGA gastric cancer bulk RNA-seq signatures. (A) Scissor mapping of bulk RNA-seq signatures derived from TCGA stomach adenocarcinoma (STAD) tumors compared with normal gastric tissues onto epithelial/tumor-cell populations from WT, C, KP, and CKP gastric tissues. (B) Scissor mapping of bulk RNA-seq signatures derived from diffuse-type tumors (DGAC) within TCGA STAD compared with normal gastric tissues onto epithelial/tumor-cell populations from WT, C, KP, and CKP gastric tissues. Red, blue, and gray dots indicate Scissor-positive, Scissor-negative, and non-significant cells, respectively. Numbers below each plot indicate Scissor-matched proliferative cells among total proliferative cells.

• *"I do not see how "These results suggest that CRACD depletion induces cell plasticity that drives the transcriptional signature of gastric neoplasia" is a conclusion that can be reached with the current analysis including the Phate maps."*

We agree that scRNA-seq alone cannot establish cellular plasticity. Accordingly, we have moderated our interpretation throughout the manuscript. We now state that the observed transcriptional changes are consistent with increased cellular plasticity and, together with our findings, support this conclusion.

Minor points

B4. *"The authors mention prior studies linking CRACD loss with neuroendocrine differentiation. This would be interesting to discuss if regulating plasticity is a core function of this gene, how it is achieved (dependent on the actin mechanism?) and if it differs between organs. Similarly, is the link between mucinous differentiation also seen in these other organs? In the germline KO mouse, does CRACD loss result in similar mucinous/cell plasticity phenotypes elsewhere?"*

In our previous study [1] (Nature Cell Biology, 2018), CRACD loss was shown to disrupt actin cytoskeletal regulation and promote the Wnt/β-catenin pathway and tumorigenesis in colorectal cancer. Consistent with this, we observe in this study that CRACD loss promotes transcriptional programs associated with mucin-related gene expression.

Mechanistically, our data support a model in which CRACD regulates actin dynamics, and its loss leads to cytoskeletal dysregulation that can influence key signaling pathways involved in cell fate determination, including YAP and NOTCH signaling. Briefly, disruption of actin polymerization by CRACD loss activates the HIPPO signaling, a mechanotransduction pathway. Subsequently, activation of the HIPPO pathway suppresses YAP and NOTCH signaling, which are required to inhibit secretory cell lineages, including mucinous and neuroendocrine lineages [3,4] (Nat Comms, Cell Reports, and unpublished manuscript). These pathways are well-known regulators of lineage plasticity and may provide a common mechanistic framework linking CRACD loss to diverse differentiation phenotypes.

Regarding neuroendocrine differentiation, we agree that this represents an additional manifestation of cellular plasticity. Rather than reflecting a single lineage-specific effect, **CRACD loss may broadly enhance cellular plasticity**, allowing tumor cells to adopt distinct lineage programs depending on the tissue context and signaling environment.

While a comprehensive analysis of tissue-specific effects is beyond the scope of the current study, we have expanded the *Discussion to more clearly address the role of CRACD in regulating actin-dependent plasticity and to integrate findings across different cancer types.*

B5. *"Fig. 3E seems to be missing."*

Fig. 3E was included in the original figure but may have been difficult to locate due to the dense panel layout. This panel corresponds to the tumor incidence graph. We have now revised the figure layout and increased the panel size to improve visibility.

B6. *"Fig. 3J-L compares CKP tumors to KP but I could not find the data for KP."*

Figures 3J-L were designed to emphasize the aggressive phenotype of CKP tumors, including their robust growth in immunocompetent C57BL/6 mice, metastatic features, and occasional signet ring cell-like morphology. In contrast, KP tumors show markedly limited tumor formation and do not generate comparable tumor burden.

For this reason, representative KP tumor images were not included in the main panels. However, we agree that the availability of these data should be clearly indicated. The corresponding KP tumor data are provided in **Supplementary Fig. 6D**, where KP tumors are visibly smaller and less developed than CKP tumors. We have now revised the manuscript to explicitly reference these data and to improve clarity in the figure presentation.

We appreciate all constructive comments that improve the manuscript.

Reviewer #3

"In this manuscript, the authors demonstrate that loss of the actin regulator CRACD drives mucinous plasticity and immune evasion in gastric cancer. Using genetically engineered murine gastric organoids with CRACD ablation combined with Kras mutation and Trp53 loss, they show that CRACD deficiency disrupts epithelial integrity and promotes the development of mucinous gastric adenocarcinoma features. Mechanistically, the authors reveal that CRACD loss leads to actin dysregulation, which activates NF-κB/COX-2 signaling and NADPH oxidase–dependent ROS production. The resulting oxidative stress stabilizes HIF1α and induces PD-L1 transcription, thereby suppressing anti-tumor immunity. Furthermore, pharmacologic inhibition of HIF1α or PD-L1 restores immune surveillance and reduces tumorigenesis. Consistent with these findings, analyses of patient-derived organoids and tissue microarrays show an inverse correlation between CRACD and PD-L1 expression. These results suggest that CRACD inactivation is a key driver of mucinous plasticity and immune escape in gastric cancer and highlight the HIF1α–PD-L1 axis as a potential therapeutic target in CRACD-deficient tumors.

The concept of this study is interesting and potentially impactful. The authors provide a solid mechanistic characterization of CRACD loss in gastric tumorigenesis. However, the clinical significance of these findings requires further clarification, and additional studies will be needed to determine whether these findings can translate into meaningful therapeutic advances. The specific comments are listed below."

Major points

C1. *"How does the effect of PX-478 compare with anti-PD-L1 therapy alone? Based on the presented data, CRACD-negative (CRACD-loss) gastric cancers appear to respond relatively well to anti-PD-L1 monotherapy. Therefore, it would be important to clarify whether the use of the HIF-1α inhibitor PX-478 provides additional benefit compared with the currently available anti-PD-L1 therapy. The potential therapeutic advantage of introducing PX-478 in this context should be discussed."*

Thank you for the critical comments. Our data suggest that HIF1α inhibition and PD-L1 blockade act at distinct levels of the same signaling cascade. While PD-L1 blockade targets tumor-immune interactions at the immune checkpoint level, HIF1α inhibition acts upstream by suppressing tumor-intrinsic hypoxia signaling and reducing PD-L1 expression itself.

Importantly, our results indicate that PX-478 not only impacts PD-L1 expression but also broadly suppresses hypoxia-driven transcriptional programs, suggesting a more comprehensive effect on tumor biology. Therefore, **PX-478 may provide additional therapeutic benefit beyond PD-L1 blockade alone**, particularly by targeting tumor-intrinsic mechanisms of immune evasion. However, there are no FDA-approved HIF1α inhibitors yet.

We have expanded the discussion to clarify this distinction and to highlight the potential complementary roles of HIF1α inhibition and PD-L1 blockade.

C2. "*Is there any association between CRACD-loss and established molecular subtypes of gastric cancer? Gastric cancer is known to consist of distinct molecular subtypes, which is strongly related to immune microenvironment and response to immunotherapy. If CRACD is proposed as a biomarker-guided candidate for PD-L1 blockade, it is important to determine whether its predictive value is independent of established biomarkers. At least this point should be discussed."*

We thank the reviewer for this important comment. In our analysis, CRACD loss did not map exclusively to a single established molecular subtype of gastric cancer. Rather, CRACD-deficient tumors exhibited overlapping features across multiple subtype contexts, including enrichment of diffuse gastric cancer-associated transcriptional signatures while also retaining histological features that overlap with intestinal-type or gland-forming gastric cancer. These findings suggest that CRACD loss may enhance a diffuse/mucinous-like cellular state rather than define a single molecular subtype.

We agree that the potential value of CRACD as a biomarker for PD-1/PD-L1 blockade should be interpreted in the context of established immunotherapy biomarkers, including PD-L1 CPS, MSI status, EBV status, and

other immune-related molecular features. Our current study does not establish that CRACD loss is superior to, or fully independent of, these established biomarkers. Instead, our data suggest that CRACD loss may identify a biologically distinct tumor state characterized by increased ROS/HIF1α signaling and altered immune-related programs, which could potentially complement existing biomarkers. *We have now clarified this point in the Discussion and acknowledge* that larger clinically annotated cohorts with matched CRACD status, PD-L1 CPS, MSI/EBV status, molecular subtype, treatment history, and response to immune checkpoint blockade will be required to determine whether CRACD provides independent or additive predictive value.

C3. *"It would be informative to examine whether CRACD-loss is associated with clinical outcomes, such as progression-free survival or overall survival, in gastric cancer cohorts. Such analysis would help clarify the clinical relevance of CRACD inactivation."*

We thank the reviewer for this important suggestion. To address this point, we examined the association between CRACD expression and clinical outcomes using an advanced gastric cancer TMA cohort consisting of 290 cases collected between 2000 and 2003. The TMA cohort consisted of a single 3-mm tissue core representing each tumor. Among these, 225 tumor cases were evaluable for CRACD immunostaining and were included in the clinicopathologic association analysis. Cases with non-evaluable tumor cores or missing CRACD staining were excluded from this analysis. For survival analysis, only cases with both evaluable CRACD staining and available overall survival data were included, resulting in 222 cases.

Overall, CRACD expression was generally low in this gastric cancer cohort, which most evaluable tumors showing either negative or weak staining. Only one case showed moderate CRACD expression, and no case showed strong expression. Therefore, given the near absence of moderate-to-strong CRACD staining, we classified cases as CRACD-negative versus CRACD-positive rather than using an intensity-based high/low cutoff. Using this classification, CRACD-negative status was not significantly associated with major clinicopathologic variables, including Lauren type, tumor stage, lymphovascular invasion, lymph node metastasis, MSI-H status, or EBV status (**Table 1**). Kaplan-Meier analysis also showed no significant association between CRACD expression and overall survival (**Fig. R8**).

For clarification, our study is not intended to establish CRACD loss as a standalone survival biomarker. Rather, our data support a functional role for CRACD loss in promoting aggressive mucinous-type gastric cancer biology, including enhanced cellular plasticity, invasive/metastatic behavior, and activation of the ROS-HIF1α-PD-L1 axis.

The lack of a significant overall survival association in this cohort may be explained by several factors, including the limited dynamic range of CRACD immunostaining, potential intratumoral heterogeneity that may not be fully captured by a single 3-mm TMA core, and the use of a heterogeneous advanced gastric cancer cohort not specifically enriched for mucinous or diffuse-type tumors. This limitation is particularly relevant for mucinous gastric cancer, which is generally defined by a substantial mucinous component within the tumor. Therefore, a single TMA core may not fully represent the mucinous histology or spatially heterogeneous CRACD expression across the whole tumor. In addition, this analysis was based on CRACD protein expression assessed by IHC and did not incorporate other potential mechanisms of CRACD inactivation, such as mutations, copy-number alterations, or epigenetic silencing. Therefore, larger independent cohorts enriched for mucinous or diffuse-type gastric cancers, ideally with matched genomic, metastatic-status, immune-marker, and treatment-response annotations, will be needed to determine the clinical relevance of CRACD loss in the disease context most relevant to our study.

Variable	Category	CRACD negative	CRACD positive	*P* value
Lauren type	Intestinal	68 (45.3)	27 (40.3)	0.554
	Diffuse	82 (54.7)	40 (59.7)	
Stage	I-II	61 (38.9)	26 (38.2)	1.000
	III-IV	96 (61.1)	42 (61.8)	
Lymphovascular invasion	Absent	106 (67.5)	48 (70.6)	0.755
	Present	51 (32.5)	20 (29.4)	
Lymph node metastasis	Absent (N0)	40 (25.5)	21 (30.9)	0.418
	Present (N+)	117 (74.5)	47 (69.1)	
MSI-H status	No	135 (87.7)	57 (83.8)	0.523
	Yes	19 (12.3)	11 (16.2)	
EBV status	Negative	147 (95.5)	62 (91.2)	0.225
	Positive	7 (4.5)	6 (8.8)	

Table 1. Association between CRACD expression and clinicopathologic variables. Data are presented as n (%). *P* values were calculated using Fisher's exact test. For Lauren type, only intestinal and diffuse types were compared; mixed and missing cases were excluded from this specific comparison. Percentages are calculated within each variable-specific valid denominator.

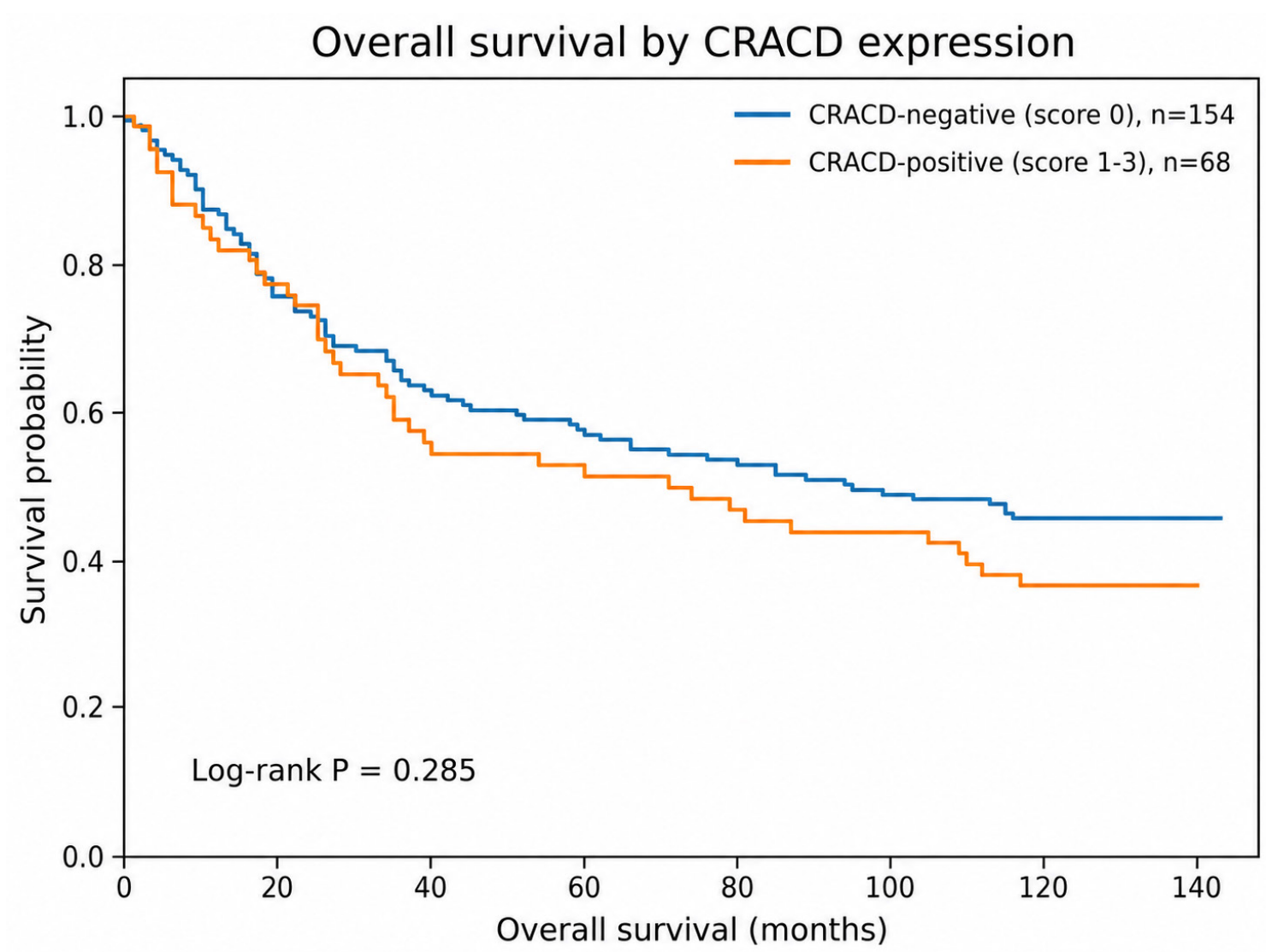

Fig. R8. Survival probability according to CRACD expression. Kaplan-Meier curve for overall survival according to CRACD expression. Log-rank *P* = 0.2850.

C4. *"In Figure 7B, the stratification into diffuse and intestinal types may potentially mislead readers. Based on Supplementary Figure 1, CRACD inactivation appears to be more frequent in the intestinal type rather than the diffuse type. In addition, diffuse-type gastric cancer is generally considered less responsive to immune checkpoint inhibitors. If the main purpose of this figure is to demonstrate the relationship between CRACD status and PD-L1 signaling, it is unclear whether presenting the histological subtype classification is necessary."*

We thank you for your thoughtful comment. While our data suggest that CRACD expression and PD-L1 signaling are associated with tumor state and differentiation features, we agree that including histological subtype stratification in Fig. 7B may introduce unnecessary complexity and could lead to over-interpretation.

We note that the primary purpose of this figure is to illustrate the relationship between CRACD status and PD-L1 signaling, rather than to establish subtype-specific associations. In this context, we agree that emphasizing histological classification is not essential.

Therefore, in response to your suggestion, we have removed the diffuse versus intestinal subtype stratification from Fig. 7B and revised the corresponding text to avoid potential confusion throughout the manuscript. Our main conclusion regarding the inverse relationship between CRACD expression and PD-L1 signaling remains unchanged.

We greatly appreciate insightful and constructive comments.

References

1 Jung, Y. S. *et al.* Deregulation of CRAD-controlled cytoskeleton initiates mucinous colorectal cancer via β-catenin. *Nat Cell Biol* **20**, 1303-1314 (2018). https://doi.org/10.1038/s41556-018-0215-z

2 Zou, G. *et al.* E-cadherin loss drives diffuse-type gastric tumorigenesis via EZH2-mediated reprogramming. *J Exp Med* **221** (2024). https://doi.org/10.1084/jem.20230561

3 Kim, B. *et al.* CRACD loss induces neuroendocrine cell plasticity of lung adenocarcinoma. *Cell reports* **43** (2024).

4 Seo, Y. *et al.* Actin dysregulation induces neuroendocrine plasticity and immune evasion: a vulnerability of small cell lung cancer. *Nature Communications* **17**, 386 (2025). https://doi.org/10.1038/s41467-025-67078-9

5 Dhar, S. S. *et al.* KMT2D temporally activates neuronal transcriptional factor genes to mediate cerebellar granule cell differentiation. *Sci Adv* **11**, eadu7174 (2025). https://doi.org/10.1126/sciadv.adu7174

6 Jang, J., Ko, K. P., Zhang, J., Jun, S. & Park, J. I. Deciphering precursor cell dynamics in esophageal preneoplasia via genetic barcoding and single-cell transcriptomics. *Proc Natl Acad Sci U S A* **122**, e2509534122 (2025). https://doi.org/10.1073/pnas.2509534122

Title: CRACD Loss Primes Gastric Cancer for Immune Escape by the Hypoxic Pathway
Recommendation: *Minor Revision*

<u>*Comments to Editor*</u>
In this manuscript, the authors demonstrate that loss of the actin regulator CRACD drives mucinous plasticity and immune evasion in gastric cancer. Using genetically engineered murine gastric organoids with CRACD ablation combined with Kras mutation and Trp53 loss, they show that CRACD deficiency disrupts epithelial integrity and promotes the development of mucinous gastric adenocarcinoma features. Mechanistically, the authors reveal that CRACD loss leads to actin dysregulation, which activates NF-κB/COX-2 signaling and NADPH oxidase–dependent ROS production. The resulting oxidative stress stabilizes HIF1α and induces PD-L1 transcription, thereby suppressing anti-tumor immunity. Furthermore, pharmacologic inhibition of HIF1α or PD-L1 restores immune surveillance and reduces tumorigenesis. Consistent with these findings, analyses of patient-derived organoids and tissue microarrays show an inverse correlation between CRACD and PD-L1 expression. These results suggest that CRACD inactivation is a key driver of mucinous plasticity and immune escape in gastric cancer and highlight the HIF1α–PD-L1 axis as a potential therapeutic target in CRACD-deficient tumors.

The concept of this study is interesting and potentially impactful. The authors provide a solid mechanistic characterization of CRACD loss in gastric tumorigenesis. However, the clinical significance of these findings requires further clarification, and additional studies will be needed to determine whether these findings can translate into meaningful therapeutic advances. Overall, considering the high scientific standards of *Nature Communications*, this reviewer believes that several minor revisions would further strengthen the manuscript and improve its clarity.

<u>*Comments to Author*</u>
In this manuscript, the authors demonstrate that loss of the actin regulator CRACD drives mucinous plasticity and immune evasion in gastric cancer. Using genetically engineered murine gastric organoids with CRACD ablation combined with Kras mutation and Trp53 loss, they show that CRACD deficiency disrupts epithelial integrity and promotes the development of mucinous gastric adenocarcinoma features. Mechanistically, the authors reveal that CRACD loss leads to actin dysregulation, which activates NF-κB/COX-2 signaling and NADPH oxidase–dependent ROS production. The resulting oxidative stress stabilizes HIF1α and induces PD-L1 transcription, thereby suppressing anti-tumor immunity. Furthermore, pharmacologic inhibition of HIF1α or PD-L1 restores immune surveillance and reduces tumorigenesis. Consistent with these findings, analyses of patient-derived organoids and tissue microarrays show an inverse correlation between

CRACD and PD-L1 expression. These results suggest that CRACD inactivation is a key driver of mucinous plasticity and immune escape in gastric cancer and highlight the HIF1α–PD-L1 axis as a potential therapeutic target in CRACD-deficient tumors.

The concept of this study is interesting and potentially impactful. The authors provide a solid mechanistic characterization of CRACD loss in gastric tumorigenesis. However, the clinical significance of these findings requires further clarification, and additional studies will be needed to determine whether these findings can translate into meaningful therapeutic advances.

The specific comments are listed below.

Comments

1. How does the effect of PX-478 compare with anti-PD-L1 therapy alone? Based on the presented data, CRACD-negative (CRACD-loss) gastric cancers appear to respond relatively well to anti-PD-L1 monotherapy. Therefore, it would be important to clarify whether the use of the HIF-1α inhibitor PX-478 provides additional benefit compared with the currently available anti-PD-L1 therapy. The potential therapeutic advantage of introducing PX-478 in this context should be discussed.

2. Is there any association between CRACD-loss and established molecular subtypes of gastric cancer? Gastric cancer is known to consist of distinct molecular subtypes, which is strongly related to immune microenvironment and response to immunotherapy. If CRACD is proposed as a biomarker-guided candidate for PD-L1 blockade, it is important to determine whether its predictive value is independent of established biomarkers. At least this point should be discussed.

3. It would be informative to examine whether CRACD-loss is associated with clinical outcomes, such as progression-free survival or overall survival, in gastric cancer cohorts. Such analysis would help clarify the clinical relevance of CRACD inactivation.

4. In Figure 7B, the stratification into diffuse and intestinal types may potentially mislead readers. Based on Supplementary Figure 1, CRACD inactivation appears to be more frequent in the intestinal type rather than the diffuse type. In addition, diffuse-type gastric cancer is generally considered less responsive to immune checkpoint inhibitors. If the main purpose of this figure is to demonstrate the

relationship between CRACD status and PD-L1 signaling, it is unclear whether presenting the histological subtype classification is necessary.
